# Integration of linkage maps for the Amphidiploid *Brassica napus *and comparative mapping with *Arabidopsis *and *Brassica rapa*

**DOI:** 10.1186/1471-2164-12-101

**Published:** 2011-02-09

**Authors:** Jun Wang, Derek J Lydiate, Isobel AP Parkin, Cyril Falentin, Régine Delourme, Pierre WC Carion, Graham J King

**Affiliations:** 1 Department of Plant Sciences, Rothamsted Research, Harpenden, AL5 2JQ, UK; 2 Agriculture and Agri-Food Canada, 107 Science Place, Saskatoon, Saskatchewan, S7N 0X2, Canada; 3 UMR 118 Amélioration des Plantes et Biotechnologies Végétales, INRA, BP 35327, 35653 Le Rheu Cedex, France; 4 Centre for Haemato-Oncology, Barts Cancer Institute, Barts and The London School of Medicine and Dentistry, Charterhouse Square, London, EC1 M 6BQ, UK; 5 Southern Cross Plant Sciences, Southern Cross University, Lismore, NSW 2480, Australia

## Abstract

**Background:**

The large number of genetic linkage maps representing *Brassica *chromosomes constitute a potential platform for studying crop traits and genome evolution within *Brassicaceae*. However, the alignment of existing maps remains a major challenge. The integration of these genetic maps will enhance genetic resolution, and provide a means to navigate between sequence-tagged loci, and with contiguous genome sequences as these become available.

**Results:**

We report the first genome-wide integration of *Brassica *maps based on an automated pipeline which involved collation of genome-wide genotype data for sequence-tagged markers scored on three extensively used amphidiploid *Brassica napus *(2n = 38) populations. Representative markers were selected from consolidated maps for each population, and skeleton bin maps were generated. The skeleton maps for the three populations were then combined to generate an integrated map for each LG, comparing two different approaches, one encapsulated in JoinMap and the other in MergeMap. The BnaWAIT_01_2010a integrated genetic map was generated using JoinMap, and includes 5,162 genetic markers mapped onto 2,196 loci, with a total genetic length of 1,792 cM. The map density of one locus every 0.82 cM, corresponding to 515 Kbp, increases by at least three-fold the locus and marker density within the original maps. Within the *B. napus *integrated map we identified 103 conserved collinearity blocks relative to *Arabidopsis*, including five previously unreported blocks. The BnaWAIT_01_2010a map was used to investigate the integrity and conservation of order proposed for genome sequence scaffolds generated from the constituent A genome of *Brassica rapa*.

**Conclusions:**

Our results provide a comprehensive genetic integration of the *B. napus *genome from a range of sources, which we anticipate will provide valuable information for rapeseed and Canola research.

## Background

*Brassica napus *is found almost solely in an agricultural setting represented by the oil crops oilseed rape (Canola, rapeseed) and vegetable/fodder crops swede and rutabaga. As one of the most commercially important oil crops, it is grown in most temperate regions of the world including North and South America, Europe, Australia, and East and South Asia, for the production of vegetable oil for human consumption, industrial uses including as a lubricant or biofuel, and a protein meal used as animal feed.

*Brassica napus *is an amphidiploid species (AC genome, n = 19) derived from a recent hybridization event between *Brassica rapa *(A genome, n = 10) and *Brassica oleracea *(C genome, n = 9) (U, 1935). It probably arose and was selected in human cultivation within the past 10,000 years. It is widely accepted that *Brassica *species diverged from a common ancestor with the *Arabidopsis *lineage ~20 MYA [1,2]. Similarly, the A and C genomes diverged from a common ancestor ~5 MYA. Since the divergence of the two lineages leading to the genera *Brassica *and *Arabidopsis*, there has been a triplication event that created a hexaploid ancestor unique to the tribe *Brassiceae *[3-7]. This is supported by evidence from ~1,300 restriction fragment length polymorphism (RFLP) loci in the *Brassica *A and C genomes that were mapped to homologous positions in *Arabidopsis *[7], along with evidence from comparative linkage mapping between *B. juncea*, *B. oleracea*, *B. rapa *and *Arabidopsis *[8-11] and FISH analysis [6]. These events occurred after ancient whole-genome duplications found in *Arabidopsis *ancestors (1-3R, or γ, β and α, respectively) [12-14]. A recent study of the distribution and rate of synonymous substitutions in homologous sequences among *Brassica *and *Arabidopsis *has suggested that the triplicated *B. rapa *(A) genome may also have undergone a process of genome shrinkage [15].

Genetic linkage maps represent a key resource to understand genome organisation, evolutionary relationships, and to assist in the assignment and orientation of sequence assemblies to correct chromosome locations. In addition, dense linkage maps provide the basis for map-based cloning of major genes and QTLs underlying agronomic traits, as well as for marker-assisted selection. In *B. napus*, a range of sequence tagged genetic markers, including restriction fragment length polymorphism (RFLPs), simple sequence repeats (SSRs) and single nucleotide polymorphisms (SNPs) have been developed both from *Arabidopsis *and *Brassica *species. Various versions of linkage maps, derived from a range of reference *B. napus *mapping populations, have been published within the last twenty years [7,16-26].

Development of a high density integrated genetic map of *B. napus *derived from well established mapping populations will provide a superior tool for high resolution mapping and verification of DNA sequence contig order and orientation. Benefits arise from incorporating information derived from the increased number of individuals and chiasmata represented within the populations. Since the parent lines are genetically diverse, a larger proportion of markers will be informative and so enable a higher number of mapped markers to be obtained from the potential number of markers available. For several crop species such as maize [27,28], soybean [29,30], barley [31-33], sorghum [34-36], wild wheat [37,38], grapevine [39,40], cowpea [41] and peanut [42], integrated consensus linkage maps of multiple mapping populations have been developed. In *Brassica*, early attempts [43] to align linkage maps derived from different *Brassica *populations were based on very low numbers of shared markers, and suffered from lack of resolution with respect to distinguishing between paralogous loci. More recent efforts have been successful in generating aligned maps for the *Brassica *A genome that integrate marker information using a common set of SSRs scored in *B. rapa *and *B. napus *[26].

Although conceptually simple, in practice construction of an integrated map from diverse sources (populations and types of markers) is a non-trivial exercise. This is particularly true where genetic maps have been generated from different populations or sub-populations with different subsets of informative genetic markers. The situation is exacerbated where multiple paralogous loci may exist as a result of chromosomal segmental duplication over relatively recent evolutionary time, which in the case of *B. napus *is compounded by amphidiploidy. This may lead to a low number of shared (bridge or anchor) markers between maps. Moreover, the quality of genotype data may vary across studies, thus hampering the progress of genetic map integration.

Several systematic approaches have been proposed to construct integrated maps. Early attempts involved pooling genotype information from several segregating populations, and then relying on conventional mapping algorithms (e.g., log-likelihood statistic) to build a single composite map [44,45]. However, this method has some shortcomings. Firstly, mapping populations may be of different types (e.g., double haploid, backcross, F2 intercross and recombinant inbred lines) and have different estimates of genetic distance. Pooling information cannot be applied to all combinations of populations, since treating data from different sources equivalently is flawed. Secondly, once a composite genotype matrix is generated from several populations it contains a large proportion of missing data, where conventional mapping algorithms will tend to generate maps of low quality. Alternative approaches have involved modification to mapping algorithms, such as employed by JoinMap [46-48] and Carthagène [49]. These software packages take into account all available information from each individual dataset (e.g., population structure and size) and estimate the marker order and genetic distances of common (anchor or bridge) markers using regression mapping (JoinMap) or multiple 2-point maximum likelihood (Carthagène). Since both methods involve exhaustive search of objective functions, the computational process to search for an optimal map is very time consuming. This becomes limiting for map integration that involves a very large number of markers and/or populations. A third approach, MergeMap [50], relies on graph theory [51,52] and uses directed acyclic graphs (DAGs) to represent maps from individual populations, and to resolve conflicts between maps. Although MergeMap does not make use of genotype data, simulations have shown that MergeMap can outperform JoinMap in terms both of accuracy and running time [50].

In this study, we report the first genome-wide integration of *Brassica *genetic maps based on an automated implementation of a defined algorithm. We selected three extensively studied *B. napus *DH mapping populations, BnaSNDH, BnaSGDH and BnaDYDH, since they share a high number of loci derived from common genetic marker assays. A range of different published and unpublished sources of genotype data have been collated and curated for each population. Our approach involved first constructing a population-specific consolidated map by merging constituent genotype matrices for each mapping population following initial assignment to each of the 19 LGs. A skeleton map that consists solely of representative markers from each bin was then prepared for the subsequent map integration for each population. We were able to compare the contrasting approaches employed by JoinMap and MergeMap, and then to investigate models of genome collinearity within the *Brassicaceae*, and the relationship between genetic and physical distances.

## Results

The first stage of the integration process involved combining map data from previously published sources with new genotype score datasets, primarily from a large number of SSR markers for each of the three DH populations. This not only increases the map density and represents more recombination events, but also for the purpose of map integration potentially provides additional 'bridge' information between populations.

### Population-specific consolidated maps for three DH populations

BnaSGDH_03_2010a is the first published map derived from the BnaSGDH population, and includes 483 RFLP and 1,897 SSR marker loci. In addition to 1,287 RFLP markers used previously in the BnaSNDH population [7,16], 1,314 SSR markers were included in the BnaSNDH_05_2010a consolidated map. In the BnaDYDH_05_2010a map, there were 356 SSR and 511 other genetic markers, including RFLPs, AFLPs, RAPDs and SNPs. The population specific genetic maps comprised 745 (BnaSNDH), 894 (BnaSGDH) and 528 (BnaDYDH) unique mapping loci (Table 1). The elimination of unlikely local double crossovers and selection of representative markers to form population-specific bin maps greatly reduced the initial inflated lengths of the LGs, by up to 50%, with average LG lengths varying from 140 to 194 cM in the three mapping populations. The lengths of LGs among all three population-specific maps were positively correlated (between BnaSNDH and BnaSGDH Spearman's correlation *r *= 0.68, *p *= 0.0016; between BnaSNDH and BnaDYDH *r *= 0.55, *p *= 0.02; and between BnaSGDH and BnaDYDH *r *= 0.49, *p *= 0.03,).

**Table 1:** Distribution of marker loci (n), shared markers (n), unique mapping loci (n) and LGs lengths (cM) within different LGs of the three population-specific *B. napus *maps, BnaSNDH_05_2010a (Map A), BnaSGDH_03_2010a (Map B) and BnaDYDH_05_2010a (Map C) and the two integrated maps, BnaWAIT_01_2010a (Map D) generated by JoinMap and BnaWAIT_01_2010b (Map E) generated by MergeMap.

	Number of markers (n)				Number of shared markers (n)			Number of unique loci (n)					LGs length (cM)				
**LG**	**Map** **A**	**Map** **B**	**Map** **C**	**Map** **D & E**	**Map** **A/B**	**Map** **A/C**	**Map** **B/C**	**Map** **A**	**Map** **B**	**Map** **C**	**Map** **D**	**Map** **E**	**Map** **A**	**Map** **B**	**Map** **C**	**Map** **D**	**Map** **E**

**A01**	145	107	60	271	26	9	8	54	52	33	134	98	265.31	227.47	159.66	100.96	440.76
**A02**	131	70	34	199	30	6	2	45	25	22	92	72	154.07	129.59	121.95	86.69	194.45
**A03**	199	166	72	385	39	9	7	62	63	44	174	141	222.34	247.10	217.88	126.10	417.02
**A04**	83	83	33	176	18	1	4	26	28	20	77	64	108.13	157.47	111.51	80.32	216.89
**A05**	113	76	41	205	19	4	3	31	40	25	87	74	123.98	206.19	123.15	100.15	213.32
**A06**	145	114	43	261	33	4	4	33	52	30	111	93	158.92	149.34	158.26	108.46	201.08
**A07**	95	100	33	211	11	3	4	42	43	24	102	91	220.08	140.65	92.85	88.08	269.32
**A08**	106	78	41	199	17	5	4	28	20	26	80	64	130.32	87.97	102.84	73.77	147.75
**A09**	148	154	70	327	29	8	8	52	68	36	153	131	236.60	256.02	154.03	103.21	454.28
**A10**	134	74	40	215	23	7	5	22	37	26	89	73	127.17	111.02	147.12	78.69	219.01
**C01**	117	141	50	280	19	4	8	32	70	23	126	104	220.52	263.74	126.82	94.59	308.63
**C02**	121	88	44	232	14	5	3	27	37	29	90	83	113.53	106.79	116.85	67.09	255.17
**C03**	255	253	64	497	57	6	13	68	75	45	200	169	266.69	317.20	240.38	132.94	516.25
**C04**	173	189	61	379	36	2	7	33	51	40	137	107	163.42	197.08	161.66	111.41	307.20
**C05**	142	141	42	284	30	5	7	41	39	21	110	79	160.35	203.83	164.19	88.80	237.87
**C06**	34	83	30	133	10	2	2	16	32	17	67	65	105.63	90.39	89.98	62.07	204.02
**C07**	150	153	42	303	30	3	12	38	45	28	117	86	180.04	225.58	139.25	90.83	278.70
**C08**	145	141	39	290	28	2	5	46	54	20	117	90	141.09	303.54	141.27	92.96	346.12
**C09**	165	169	28	315	41	2	4	49	63	19	133	112	193.31	258.75	97.31	104.75	319.60

**total**	2601	2380	867	5162	510	87	110	745	894	528	2196	1796	3291.51	3679.72	2666.96	1791.87	5547.44

### Segregation distortion within the three DH populations

Comparison of the three DH populations indicated that the proportion of mapped loci displaying segregation distortion (*p *< 0.05 in the χ^2 ^test) varied from 22% to 49% (Table 2). The proportion of loci showing segregation distortion within the BnaSNDH_03_2005a map [7], 18.3%, was slightly lower than that within our consolidated BnaSNDH map BnaSNDH_05_2010a.

**Table 2:** Segregation distortion within the three *B. napus *DH populations, BnaSNDH, BnaSGDH and BnaDYDH.

	BnaSNDH		BnaSGDH		BnaDYDH	
**LG**	**Number of loci showing segregation distortion favouring**		**Number of loci showing segregation distortion favouring**		**Number of loci showing segregation distortion favouring**	
	
	**female**	**male**	**female**	**Male**	**female**	**Male**

**A01**	5	5	0	5	8	2
**A02**	4	6	0	17	0	21
**A03**	31	0	12	0	23	0
**A04**	2	0	12	2	1	1
**A05**	1	0	1	6	0	13
**A06**	0	0	1	3	0	26
**A07**	1	1	0	36	5	6
**A08**	6	0	0	2	2	0
**A09**	0	6	64	0	19	6
**A10**	0	7	24	1	2	1
**C01**	13	0	13	5	7	0
**C02**	3	0	35	0	19	0
**C03**	11	1	0	31	1	36
**C04**	10	0	1	31	10	0
**C05**	12	4	35	0	12	0
**C06**	11	0	32	0	15	0
**C07**	0	8	0	8	4	4
**C08**	2	8	28	0	0	4
**C09**	9	0	17	31	0	16

**total**	121	46	275	178	132	136

Loci that showed segregation distortion were detected by the calculation of locus genotype frequency where the χ^2 ^test is significant (*p *< 0.05).

The most extreme segregation distortion in BnaSNDH was observed in LG A03, with 31 out of 62 loci (50%) mostly clustered in the top arm. The BnaSNDH A03 showed an average skewed ratio of 1.65:1 over its entire length (χ^2 ^= 174.02, *p *< 0.0001), favouring alleles from SYN1, the female parent. In BnaSGDH, several LGs showed segregation distortion along almost the entire lengths (> 80% of the LG length). For example, all 32 loci in C06 showed segregation distortion (a skewed ratio of 4.39:1 over the entire length, χ^2 ^= 758.41, *p *< 0.0001), favouring alleles from female line PSA12. In BnaDYDH, the most extreme case of segregation distortion was found on A02 where 21 out of 22 loci (95.4%) showed segregation distortion, favouring alleles from the male parental line Yudal. The BnaDYDH A02 showed an average skewed ratio of 1:1.85 instead of 1:1 over its entire length (χ^2 ^= 161.04, p < 0.0001).

### Conservation of marker orders between populations

Comparison of marker orders between the three population-specific consolidated maps indicated good agreement over most of the LGs (Additional File 1 generated by MapChart 2.1). Marker orders were strongly positively correlated between BnaSNDH and BnaSGDH, with a mean correlation coefficient of 0.88 (Table 3). A07 was an exception (*p *= 0.09). This could result from an observed inversion between BnaSGDH and the other two maps on A07 (Additional File 1). For some LGs, the number of shared markers was very low between populations (e.g., ≤3 shared markers for 7 LGs between BnaSNDH and BnaDYDH, and for 4 LGs between BnaSGDH and BnaDYDH, Table 1). In these cases it was difficult to judge the overall consistency of marker order among maps, as reported by correlation coefficients. Thus there was no significant correlation reported with many BnaDYDH LGs. However, marker order was conserved within those LGs where sufficient bridge markers (more than 4 shared markers) allowed for assessment of statistical significance (Table 3). This provided more confidence for the subsequent use of bridge markers for the map integration.

**Table 3:** Spearman's rank correlation (*r*) of the marker order for the comparison among the three population-specific *B. napus *maps, BnaSNDH_05_2010a, BnaSGDH_03_2010a and BnaDYDH_05_2010a, comparison between each of the three population-specific maps and each of the two integrated maps, BnaWAIT_01_2010a generated by JoinMap and BnaWAIT_01_2010b generated by MergeMap and comparison between the two integrated maps, BnaWAIT_01_2010a and BnaWAIT_01_2010b.

LG	Map	BnaSNDH_05_2010a	BnaSGDH_03_2010a	BnaDYDH_05_2010a	BnaWAIT_01_2010a
**A01**	**BnaSGDH_03_2010a**	0.96****			
	**BnaDYDH_05_2010a**	0.97***	0.97***		
	**BnaWAIT_01_2010a**	0.97****	0.98****	0.99****	
	**BnaWAIT_01_2010b**	0.99****	1.00****	1.00****	0.97***

**A02**	**BnaSGDH_03_2010a**	0.95****			
	**BnaDYDH_05_2010a**	0.94*	1.00		
	**BnaWAIT_01_2010a**	0.99****	0.99****	1.00****	
	**BnaWAIT_01_2010b**	1.00****	0.99****	1.00****	0.99****

**A03**	**BnaSGDH_03_2010a**	0.99****			
	**BnaDYDH_05_2010a**	0.97***	0.96**		
	**BnaWAIT_01_2010a**	1.00****	0.99****	1.00****	
	**BnaWAIT_01_2010b**	1.00****	1.00****	0.98****	0.99****

**A04**	**BnaSGDH_03_2010a**	0.93****			
	**BnaDYDH_05_2010a**	-------------	0.20		
	**BnaWAIT_01_2010a**	0.99****	0.97****	0.86****	
	**BnaWAIT_01_2010b**	1.00****	1.00****	0.98****	0.95****

**A05**	**BnaSGDH_03_2010a**	0.90****			
	**BnaDYDH_05_2010a**	0.20	1.00		
	**BnaWAIT_01_2010a**	0.94****	0.97****	1.00****	
	**BnaWAIT_01_2010b**	1.00****	0.99****	1.00****	0.96****

**A06**	**BnaSGDH_03_2010a**	0.84****			
	**BnaDYDH_05_2010a**	1.00	1.00		
	**BnaWAIT_01_2010a**	0.96****	0.94****	1.00****	
	**BnaWAIT_01_2010b**	0.97****	0.98****	1.00****	0.95****

**A07**	**BnaSGDH_03_2010a**	0.54			
	**BnaDYDH_05_2010a**	1.00	-0.80		
	**BnaWAIT_01_2010a**	0.87****	0.76****	1.00****	
	**BnaWAIT_01_2010b**	1.00****	0.98****	0.98****	0.85****

**A08**	**BnaSGDH_03_2010a**	0.98****			
	**BnaDYDH_05_2010a**	0.70	0.80		
	**BnaWAIT_01_2010a**	0.79****	0.46****	0.58****	
	**BnaWAIT_01_2010b**	1.00****	0.98****	0.98****	0.64****

**A09**	**BnaSGDH_03_2010a**	0.75****			
	**BnaDYDH_05_2010a**	0.95***	0.93**		
	**BnaWAIT_01_2010a**	0.86****	0.91****	0.97****	
	**BnaWAIT_01_2010b**	0.96****	0.99****	1.00****	0.92****

**A10**	**BnaSGDH_03_2010a**	0.97****			
	**BnaDYDH_05_2010a**	0.93**	0.90		
	**BnaWAIT_01_2010a**	0.99****	0.99****	0.92****	
	**BnaWAIT_01_2010b**	1.00****	1.00****	0.98****	0.97****

**C01**	**BnaSGDH_03_2010a**	0.97****			
	**BnaDYDH_05_2010a**	1.00	0.93**		
	**BnaWAIT_01_2010a**	0.99****	0.96****	0.97****	
	**BnaWAIT_01_2010b**	1.00****	0.99****	1.00****	0.97****

**C02**	**BnaSGDH_03_2010a**	0.64*			
	**BnaDYDH_05_2010a**	0.70	1.00		
	**BnaWAIT_01_2010a**	0.99****	0.88****	0.99****	
	**BnaWAIT_01_2010b**	1.00****	0.99****	1.00****	0.96****

**C03**	**BnaSGDH_03_2010a**	0.91****			
	**BnaDYDH_05_2010a**	0.66	0.93****		
	**BnaWAIT_01_2010a**	0.96****	0.96****	0.95****	
	**BnaWAIT_01_2010b**	0.98****	1.00****	1.00****	0.96****

**C04**	**BnaSGDH_03_2010a**	0.86****			
	**BnaDYDH_05_2010a**	1.00	0.96**		
	**BnaWAIT_01_2010a**	0.89****	0.96****	0.99****	
	**BnaWAIT_01_2010b**	0.99****	0.98****	1.00****	0.92****

**C05**	**BnaSGDH_03_2010a**	0.95****			
	**BnaDYDH_05_2010a**	0.70	1.00***		
	**BnaWAIT_01_2010a**	0.99****	0.98****	0.47***	
	**BnaWAIT_01_2010b**	1.00****	1.00****	1.00****	0.81****

**C06**	**BnaSGDH_03_2010a**	0.69*			
	**BnaDYDH_05_2010a**	1.00	1.00		
	**BnaWAIT_01_2010a**	0.63****	0.82****	1.00****	
	**BnaWAIT_01_2010b**	0.97****	0.97****	1.00****	0.72****

**C07**	**BnaSGDH_03_2010a**	0.91****			
	**BnaDYDH_05_2010a**	1.00	0.78**		
	**BnaWAIT_01_2010a**	0.99****	0.99****	0.99****	
	**BnaWAIT_01_2010b**	1.00****	0.98****	1.00****	0.99****

**C08**	**BnaSGDH_03_2010a**	0.96****			
	**BnaDYDH_05_2010a**	1.00	0.70		
	**BnaWAIT_01_2010a**	0.92****	0.96****	1.00****	
	**BnaWAIT_01_2010b**	1.00****	1.00****	1.00****	0.94****

**C09**	**BnaSGDH_03_2010a**	0.94****			
	**BnaDYDH_05_2010a**	1.00	1.00		
	**BnaWAIT_01_2010a**	0.99****	0.99****	0.98****	
	**BnaWAIT_01_2010b**	0.99****	1.00****	1.00****	0.99****

The level of significance is shown by asterisks. Significance is shown as: * < 0.05; ** < 0.01; *** < 0.001; **** < 0.0001.

### Integration of genetic maps using JoinMap and comparison with population-specific maps

**Figure 1:**
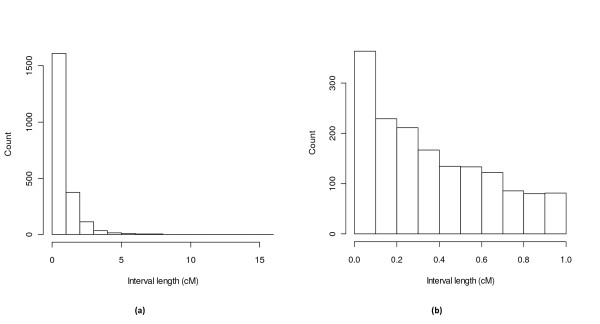
**The distribution of map intervals for the BnaWAIT_01_2010a integrated map generated by JoinMap**. It is highly skewed with an abundance of shorter distances. a) shows the distribution from 0-1 cM to >10 cM sections. b) shows the further partition of interval length distribution within the 0-1 cM section.

The BnaWAIT_01_2010a integrated linkage map contains 5,162 markers representing 2,196 unique loci (i.e. unique map positions and bins) (Table 1, Additional File 2 and 3). Map integration using JoinMap 4.0 was based on representative markers from the population-specific bin-maps, including ~20% of all markers as bridge markers across populations (Additional File 3). The A genome is represented by 2,449 markers and the C genome 2,713 (Table 1). The total genetic length for the integrated maps is 1,792 cM, with a mean length of 94.3 cM per LG. The lengths of LGs for BnaWAIT_01_2010a in relation to all three population-specific maps were significantly positively correlated (for BnaSNDH Spearman's correlation *r *= 0.74, *p *= 0.0005; for BnaSGDH *r *= 0.74, *p *= 0.0004; for BnaDYDH *r *= 0.68, *p *= 0.002). Although on average there are 2.3 markers per map interval, this ranges from one to 20. The mean map density is a locus per 0.82 cM (1,792 cM/2,196 positions). This corresponds to a locus every 515 Kbp, based on the estimated size of 1,132 Mbp [53,54] for the *B. napus *genome. The distribution of map intervals was highly skewed, with a preponderance of shorter distances (Figure 1). The marker density was 1 marker every 0.35 cM (1,792 cM/5,162 markers), or 1 marker every 219 Kbp (1,132 Mbp/5,162 markers).

**Figure 2:**
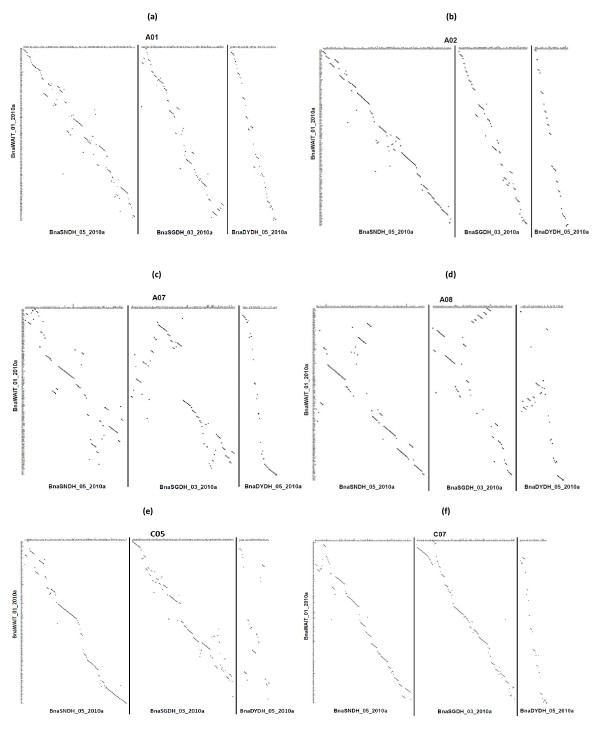
**Comparisons of marker orders between the BnaWAIT_01_2010a integrated map and each population-specific consolidated map, BnaSNDH_05_2010a, BnaSGDH_03_2010a and BnaDYDH_05_2010a**. The vertical axis indicates the BnaWAIT_01_2010a integrated map, and the horizontal axis indicates the three population-specific maps with solid vertical lines separating them. LG (a) A01, (b) A02, (c) A07, (d) A08, (e) C05 and (f) C07 are shown. A01, A02 and C07 display a good marker order consistency between the BnaWAIT_01_2010a map and component maps, and A07, A08 and C05 show relatively low level of agreement. Remainder of dot-plots are shown in Additional File 4.

Comparison of the marker order between the population-specific and integrated maps indicated overall good agreement (Figure 2; Additional File 4; Table 3). For 11 LGs, there was good agreement between the integrated and population-specific maps (Spearman's correlation *r *> 0.90 for all three pairwise comparisons). For a further five LGs the agreement in marker order was good for two of the pairwise comparisons (*r *> 0.90). For A07, A08, C05 and C06 there was a relatively low level of agreement, although the marker order was still significantly positively correlated between the three component and integrated maps. This could be due to the local order discrepancies between component maps. When there are inversions in specific populations, the use of an integrated map alone may not be informative. Map alignment of different populations (presented in Additional File 1) and dot-plots (presented in Figure 2 and Additional File 4) became powerful tools to indicate genetic regions where maker order differs among population-specific maps.

Since there were very few markers in common between some LGs, it implied that the integrated map BnaWAIT_01_2010a was likely the best estimate of a map. JoinMap 4.0 generates two alternative maps (Round 1 and Round 2 under the algorithm of regression) where a group of poorly fitting representative markers in the skeleton map were excluded from the analyses. We reported the two alternative integrated maps and the Spearman's rank correlation test between these two integrated maps and population-specific maps for all 19 LGs (Additional File 5). The BnaWAIT_01_2010a integrated map appeared to be the best estimate of the integrated map for almost all 19 LGs, compared with the other two alternative maps, except for LG C05. But 17 and 10 poorly linked representative markers were excluded from the two alternative integrated maps for C05, respectively (Additional File 5). In general, the marker orders were much conserved (*r *> 0.95) among all three integrated maps generated by JoinMap (all three rounds).

### Integration of genetic maps using MergeMap and comparison with JoinMap

We compared the pipeline incorporating JoinMap with that using MergeMap. The integrated map produced by MergeMap, BnaWAIT_01_2010b, had a total genetic length of 5,547 cM, consisting of 1,796 loci (Table 1). The map density was thus one map position every 630 Kbp, lower than that produced by JoinMap (one position every 515 Kbp). Compared with JoinMap, MergeMap tended to generate integrated maps with much higher consistency of marker order compared with each population-specific map, with Spearman's correlation coefficients >0.95 across all LGs for all three populations (Table 3).

Comparison and calculation of the Spearman's rank correlation in the marker orders for the integrated maps generated by JoinMap and MergeMap (Table 3) indicated a good agreement between the two methods for most of the LGs. Fifteen LGs had Spearman's correlation coefficients >0.90. Not surprisingly, the four LGs with correlation coefficients <0.90 were those where JoinMap performed relatively poorly for the map integration (A07, A08, C05 and C06). MergeMap appeared to outperform JoinMap in terms of marker order consistency between integrated maps and population-specific maps (especially for A07, A08, C05 and C06). One should note that MergeMap achieved this by relying solely on the existing marker orders for each component maps, rather than making use of the information within the genotype data to perform the map re-calculation. It is clear that JoinMap tended to produce more accurate estimates of genetic distances and resolve a greater number of unique marker loci for each LG compared with MergeMap (Table 1).

### Comparative mapping of *B. napus *and *Arabidopsis*, and resolution of collinearity blocks

Since the BnaWAIT_01_2010a integrated map increased the marker density by more than 3 fold compared with the BnaSNDH_03_2005a map [7], we were able to refine the recognised collinearity blocks and resolve additional blocks within the *B. napus *genome. Sequence data were obtained for RFLPs and 'BBSRC', 'Celera' and 'AAFC' SSR canonical marker assays. Homologous loci were identified within the *Arabidopsis *genome (Additional File 3 and 6).

We incorporated previously calculated homology results for 99 RFLP markers (prefixed 'es', 'I', 'N', 'R', 'T' and 'Z') from Parkin *et al. *[7], which had been established with slightly less stringent criteria. We also identified homologous loci within sequenced *B. rapa *BAC clones for RFLP and SSR canonical markers, and used the annotation of 984 *B. rapa *BAC clones (*Brassica *Genome Gateway: http://brassica.bbsrc.ac.uk/) to infer the putative *Arabidopsis *gene homology for markers whose relationship to *Arabidopsis *sequence could not be identified directly. However, this only increased the proportion of markers in the integrated map with homology in *Arabidopsis *by 1.0%. Local marker order was rearranged for 2.8% of markers based on physical proximity within sequenced *B. rapa *BAC clones. Additional homology information was obtained for some PCR markers designed from *Arabidopsis *sequences mapped in BnaDYDH (ACGM from Fourmann *et al. *[55] and specific PCR markers prefixed 'At', Delourme *et al. *[25]). In total, 41.0% of all genetic markers in the BnaWAIT_01_2010a integrated map (2,114/5,162) displayed homology to *Arabidopsis*, representing 39.2% of all mapped loci in the BnaWAIT_01_2010a integrated map. All the information of high-scoring segment pairs (HSP) and their relations to the BnaWAIT_01_2010a integrated map are available in Additional File 3.

For the identification of collinearity blocks conserved between *B. napus *and *Arabidopsis *genomes, we employed similar criteria to Parkin *et al. *[7]. A conserved block was defined as being supported by at least four homologous loci with at least one shared locus within every 5 cM in *B. napus*, and at least one shared locus within every 1 Mb in *Arabidopsis*. Based on these criteria, we detected 103 collinearity blocks in the *B. napus *genome in relation to *Arabidopsis*, of which 45 showed a significant correlation in the marker order for shared loci between *B. napus *and *Arabidopsis *(*p *< 0.05, Additional File 7). Each block contained on average 12 shared loci, and had an average length of 10.0 cM in *B. napus *and 2.8 Mb in *Arabidopsis*. The blocks represent 1,026 cM of the *B. napus *integrated map (57.3% of the mapped length) and 87.6 Mb (74.2%) of the *Arabidopsis *genome sequence. It appeared that the mapped genetic lengths of conserved blocks were significantly positively correlated with the aligned physical chromosomal lengths *of Arabidopsis *across all blocks (Spearman's correlation *r *= 0.64, *p *= 2.84e-13). The longest conserved block in terms of genetic length was BnaWAIT_A_26 in A05 with the genetic length of 49.1 cM (49.0% of the LG length), supported by 30 shared loci. The block with the highest number of shared loci was BnaWAIT_C_49 in C09 (44). The longest block in terms of aligned physical length was BnaWAIT_A_20 in A04 which was aligned to 10.9 Mb of *Arabidopsis *chromosome 2 (*Arabidopsis *blocks C2B and C2C).

Consistent with previous findings, we also found evidence of inversions and internal duplications within LGs relative to *Arabidopsis *(Additional File 7). In A07, the blocks arising from chromosomal segmental duplications, BnaWAIT_A_38 and BnaWAIT_A_39, were adjacent to each other with reversed orientation, consistent with an inverted duplication block (IDB *sensu *[56]). This has also been observed in the homeologous chromosome C06 in *Brassica oleracea *[57,58] and *B. napus *[56]. There was also evidence that some blocks overlapped with each other, and that some blocks were nested within other blocks. The overlapping genetic distances between blocks (also including blocks which were nested within another block) varied from 0.5 cM up to 10.1 cM within LGs (Additional File 7).

### Genome duplication within the *Brassica *genome

**Figure 3:**
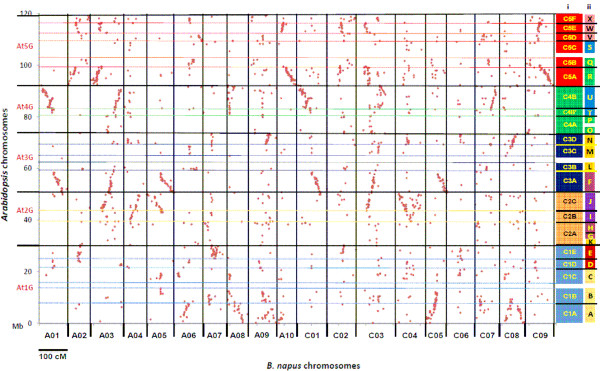
**Genome duplication within the *B. napus *genome relative to five *Arabidopsis *chromosomes**. Each dot represents an alignment between a genetic marker of *B. napus *and its homology BLAST hit within *Arabidopsis *chromosomes. i) the *Arabidopsis *blocks used in Parkin *et al. *[7], ii) the ancient karyotype (AK) blocks from Schranz *et al. *[83] are shown alongside the dot-plots aligned to their *Arabidopsis *chromosomal positions.

The BnaWAIT_01_2010a integrated map enabled us to investigate the global genome organization of *B. napus *relative to the *Arabidopsis *genome. Consistent with previous observations [7] there were between 5 and 8 conserved collinearity blocks distributed across the 19 *B. napus *chromosomes for each *Arabidopsis *block (Figure 3). It appeared that the *Arabidopsis *blocks adopted in the BnaSNDH_03_2005a map [7] were sufficient to describe the pattern of genome triplication in the BnaWAIT_01_2010a map. There was stronger evidence for genome triplication within *Brassica *for some *Arabidopsis *blocks compared with others, supported by a higher number of shared loci and longer continuous collinearity block between the two genera across LGs (e.g. blocks C1A, C1B, C2C, C3A, C3 D, C4B, C5A and C5E). *Arabidopsis *chromosomal regions having at least 5 continuous homologous copies within *B. napus *covered approximately 80% of the *Arabidopsis *genome (Figure 3).

### Comparative mapping of *B. napus *and *B. rapa *A genome

The BnaWAIT_01_2010a integrated map also enabled us to investigate the A genome evolutionary dynamics since the hybridization with the C genome. We mapped all sequence tagged markers used in the BnaWAIT_01_2010a integrated map onto the *B. rapa *A genome anchored scaffolds (The *Brassica rapa *Genome Sequencing Project Consortium [59]) for each chromosome, and compared the marker order of genetic distances (cM) with that of physical distances (Mb) using dot-plots and rank correlation.

**Figure 4:**
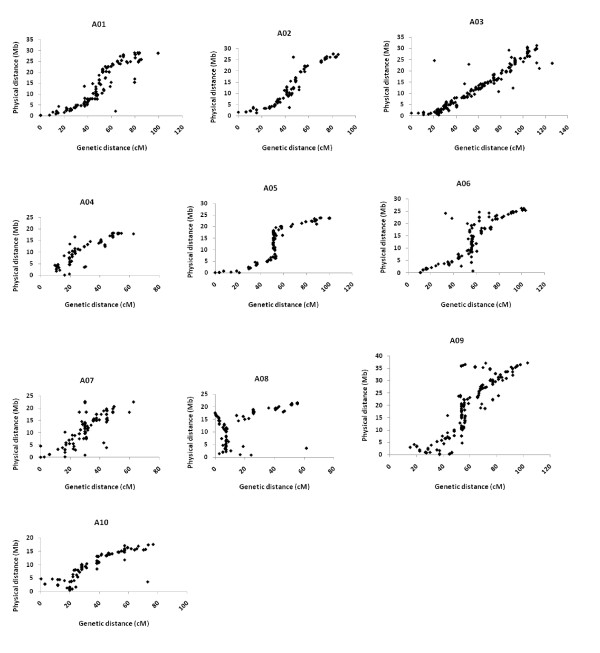
**Indication of relationship between genetic distance and physical distance for the ten *Brassica *A genome chromosomes**. Genetic distance (cM) is derived from the *B. napus *BnaWAIT map. physical distance (Mb) is derived from concatenated scaffolds of *B. rapa *Chiifu-401. The orientation of the genetic map for each LG is consistent with that of Parkin *et al. *[7]. Each marker represents a unique alignment of sequence for a marker within the genetic map against the corresponding sequence scaffold.

Marker order was globally conserved between the *B. napus *A genome integrated map and the *B. rapa *A genome anchored scaffolds across all 10 chromosomes despite some local discrepancies (Figure 4, Table 4). In A03, the correlation between the genetic length and the physical length appeared to be almost linear across the entire chromosome. The poorest correspondence between genetic and physical maps was found in A08 (Spearman's correlation *r *= 0.65, *p *< 0.0001). In some regions of the genome, the local order was clearly shown to be inconsistent between the integrated genetic map of *B. napus *A genome and the *B. rapa *genome scaffolds, such as the top section of LG A08 (0 - 8 cM) (Figure 4). The local correlation between the genetic distance and the physical distance in this region (*r *= -0.62, *p *< 0.05) appeared to be of opposite sign to the global correlation for the whole chromosome. This appeared to result from the fact that more than half of the loci in this region were physically mapped to the bottom of the chromosome (10 - 17 Mb). Moreover, this region of 8 cM (10.8% of the genetic length of A08) covered ~15 Mb of physical length (~75% of the whole chromosome physical length). Interestingly, A08 also had the lowest correlations of marker order between population-specific maps and the BnaWAIT_01_2010a integrated map (Table 3). In A05, both ends of the chromosome (0 - 5 Mb and 20 - 25 Mb) together corresponded to ~90% of the genetic length. We further investigated two additional LGs, A07 and A09, with relatively low correlations of marker order between population-specific maps and the integrated map, compared with other LGs in the A genome (Table 3). Both LGs also showed relatively lower correlations in the marker order between the integrated genetic map and the physical *B. rapa *genome sequences (*r *= 0.80 for A07, *r *= 0.86 for A09, Table 4).

**Table 4:** Spearman's rank correlation (*r*) of the marker order of the integrated map BnaWAIT_01_2010a, the three population-specific maps, BnaSNDH_05_2010a, BnaSGDH_03_2010a and BnaDYDH_05_2010a, against the physical *B. rapa *A genome scaffolds.

LG	BnaSNDH_05_2010a	BnaSGDH_03_2010a	BnaDYDH_05_2010a	BnaWAIT_01_2010a
**A01**	0.97****	0.95****	0.93****	0.96****
**A02**	0.96****	0.98****	0.84****	0.97****
**A03**	0.97****	0.96****	0.92****	0.97****
**A04**	0.95****	0.92****	0.94****	0.92****
**A05**	0.95****	0.95****	0.91****	0.91****
**A06**	0.91****	0.86****	0.92****	0.92****
**A07**	0.79****	0.72****	0.87	0.80****
**A08**	0.96****	0.98****	0.23	0.65***
**A09**	0.88****	0.64****	0.91****	0.86****
**A10**	0.95****	0.97****	0.76****	0.95****

The level of significance is shown by asterisks. Significance is shown as: * < 0.05; ** < 0.01; *** < 0.001; **** < 0.0001.

We then carried out the comparison of marker order between each population-specific consolidated map and the *B. rapa *genome scaffolds using rank correlation. It showed that for most of the LGs, the correlation coefficient was >0.85 for all three individual population-specific maps in relation to the physical *B. rapa *scaffolds. This correlation was relatively weaker for LGs A07, A08 and A09 (Table 4). Interestingly, for A08, both BnaSNDH_05_2010a and BnaSGDH_03_2010a maps showed very high correlations, but the BnaDYDH_05_2010a showed a very poor correlation with the physical *B. rapa *scaffolds (Table 4). The BnaSGDH_03_2010a map also showed a similar pattern of discrepancy against the physical *B. rapa *sequence in A09. The marker order discrepancies between some population-specific maps and the physical *B. rapa *sequence for some LGs may derive from the genome structural variation (deletion, inversion and translocation) between populations.

## Discussion

Over the past two decades more than 20 substantial genetic maps have been published for different *Brassica *species but little concerted efforts has been made to align maps from different populations. We have collated both published and previously unpublished genome-wide genotype data for sequence-tagged RFLP and SSR markers scored on three widely used *Brassica napus *populations of doubled haploid lines (BnaSNDH, BnaSGDH and BnaDYDH).

Constituent genotype matrices for each of the 19 linkage groups (LGs) were first combined to generate a consolidated genetic map for each population. Integration of component genetic maps involved selection either of bridge markers shared between populations or of markers with the highest information content to represent each unique mapping locus (bin). The skeleton bin maps for the three populations were then combined to generate an integrated map for each LG, comparing two different approaches, one encapsulated in JoinMap and the other in MergeMap. JoinMap made use of the full set of available genotype scores whilst MergeMap made use of the marker orders and cM distances of the component maps. Although the performance of MergeMap depends on the quality and accuracy of marker order within component maps, this approach has been shown to outperform JoinMap both in terms of accuracy and running time based on simulated data [50], and has been used successfully to construct integrated maps in barley [60] and cowpea [41].

In the present study, a relatively low proportion of marker loci (20.2%) were common to at least two populations. This may not provide sufficient information to overcome a few cases of uncertainty in locus order that were present in the component maps (e.g., between BnaSGDH and BnaDYDH for A04 and A07, and between BnaDYDH and the other two maps for C06, Additional File 1). However, for the purpose of map alignment/integration, the consistency of order among common markers between individual maps appeared to be more important than simply the number of shared loci. Our results demonstrate that the marker order was generally well conserved (i.e., a high level of collinearity) in the component maps, which provided a good foundation for the subsequent map integration analyses. Indeed, both JoinMap and MergeMap generated integrated maps with good consistency in marker order (measured by Spearman's rank correlation coefficient *r*) compared with component population-specific maps for most LGs (JoinMap, *r *> 0.90 for all three pairwise comparisons for 11 LGs; MergeMap, *r *> 0.95 for the three pairwise comparisons for all 19 LGs). MergeMap improved the marker order consistency for some LGs where JoinMap performed relatively poorly (e.g., A07, A08, C05 and C06).

There may be several reasons why JoinMap appeared to perform relatively poorly for some LGs. This includes the low number of shared 'bridge' markers between component maps which may hide underlying conflicts in genotype ordering that is accessible to JoinMap and not used by MergeMap. Resolving such conflicts in marker order is relatively straightforward for MergeMap as it makes use of directed acyclic graphs (DAG) to generate a single directed graph according to their shared vertices. Any ordering conflict between individual maps resulted in cycles in the combined graph. MergeMap then resolves the cycles (conflicts) by identifying and eliminating a small number of marker occurrences from some of the maps after weighting marker order differences. MergeMap only requires the marker order and cM distances of the component maps rather than the data of original genotype scores of individual populations. Thus it may be possible for consistent errors in the marker order or interval lengths in a majority of component maps to be incorporated into the integrated maps. However, in this study we can be reasonably confident that the component maps were a reliable representation of *B. napus *chromosomes, since the maps from independent populations and in different laboratories generated similar marker order. MergeMap was therefore expected to produce a relatively reliable marker order in the integrated map. In contrast, JoinMap is constrained by its need to resolve a consistent marker order in the integrated map based on a limited number of mean recombination frequencies and combined LOD scores. For both methods, when the degree of marker order inconsistency increases between individual maps, the performance becomes relatively inferior. Establishing the thresholds of such inconsistencies will be important for more extensive map integration where larger numbers of maps and/or reduced numbers of bridge markers are available.

Furthermore, one should note that there would be always conflicting markers between/among different component maps to be merged (Table 4). These conflicts of marker orders could be derived from the genome structural variation (deletion, inversion and translocation) between populations for some LGs or mapping errors. Thus, low correlations between the integrated map and a particular population-specific map, along with good correlations between the integrated map and the other two component maps (Table 3 and 4), could be indications of genome rearrangements in one of the populations. Further investigation of the dot plots (Figure 2 and Addition File 4) may identify the event(s) which creates such marker order conflicts.

As part of the pre-processing of genotype data prior to map integration, we carried out a masking of genotype scores where single data points were eliminated where a single locus was flanked by a double crossover. This process provides more consistent genetic lengths for specific linkage groups, and more realistic lengths between adjacent crossovers that represent exchange of large chromosomal regions. This process may also eliminate some actual genetic exchanges. However, since these would be short they will have only a small effect on the final map. Following this procedure a degree of map inflation still remained compared with those published previously for BnaSNDH [7,16] and BnaDYDH [19,25], which is often encountered when large numbers of markers are employed due to the cumulative effect of the low background error rate. Any overestimation of genetic length is incorporated into integrated maps calculated by MergeMap. In contrast, JoinMap makes use of all available pairwise recombination frequencies and LOD scores, and so LG lengths were closer to expectation and appeared more reliable, with good agreement with previously published component maps. In addition, JoinMap was also able to resolve a greater number of unique marker loci across all LGs, increasing the number of loci by 22.8% compared with MergeMap.

The heuristic method employed in MergeMap greatly enhances the speed of map integration compared with the regression mapping algorithm employed in JoinMap, especially where large genotype matrices are used. Indeed JoinMap is limited by the matrix size for dense maps, and so the problem needs to be broken down into sub-problems, either by bin mapping as we have done here, or by taking overlapping sub-sections of LGs, which does not provide an ideal solution. Pragmatically, where accurate estimates of genetic distances are not the priority, MergeMap provides a rapid and relatively reliable solution, especially where component maps have been generated with consistently low error rates for marker scores. The MergeMap algorithm has been successfully applied for map integration where either a large number of genetic markers are involved, such as high-throughput SNP genotyping [60], or where genotyping data were not available for many published genetic maps [61]. However, JoinMap still performed well in map integration based on our map construction procedure for the three *B. napus *DH populations.

Overall, the BnaWAIT_01_2010a integrated map generated by the JoinMap method included 5,162 markers, compared with 1,317 markers in the previous reference BnaSNDH map of Parkin *et al. *[7] and 866 markers in the BnaDYDH map reported by Delourme *et al. *[25]. This increased the marker density by 3.3 and 5.8 fold, respectively. Furthermore, the nine LGs representing C genome chromosomes contain 11.6% more markers and 11.8% more loci than the ten LGs representing A genome chromosomes in the BnaWAIT_01_2010a map. This is in close agreement with the estimated 16% larger size of the C genome [53,54].

The BnaWAIT_01_2010a integrated map enabled us to test existing models of collinearity between *Arabidopsis *and *Brassica*. This analysis was based on twice as many markers where sequence similarity to *Arabidopsis *could be identified, compared with the BnaSNDH map of Parkin *et al. *[7]. We identified 103 conserved colinearity blocks in *B. napus *relative to *Arabidopsis*. These corresponded to almost all 97 *B. napus *blocks reported in the BnaSNDH_03_2005a map, although we did not resolve 17 short blocks previously identified based solely on RFLP markers [7]. Although the same homology hits were identified between the *Arabidopsis *genome and 50 RFLPs within these 17 short blocks, the criteria to define a collinearity block (i.e., four homologous loci with at least one shared locus within every 5 cM in *B. napus *and at least one shared locus within every 1 Mb in *Arabidopsis*) were not met in our study. Moreover, these short blocks only represented <5.0% of the total mapped length of the BnaSNDH_03_2005a map. Five previously unreported collinearity blocks were identified in our study. However, these new blocks covered only 14.5 cM of genetic length in total, aligned to 7.0 Mb in *Arabidopsis *chromosomes 3 and 5. We further established that the synteny order of the 48 collinearity blocks within the A genome of *B. napus *in BnaWAIT_01_2010a is essentially the same as that established in *B. juncea *based on intron polymorphism (IP) markers [10]. This indicates that synteny order is highly conserved in the A genomes of *B. juncea *and *B. napus*.

We attempted to align 3,837 primer sequence pairs for the SSR markers to the *Arabidopsis *chromosomes to identify homology with the resultant target 'virtual PCR product' of primers. However, <2% of the primer pairs had homology in *Arabidopsis*, of which only 50% agreed with those identified using the corresponding SSR clone sequences. This suggests that future comparative studies within the *Brassicaceae *based solely on SSR primer sequences are unlikely to provide useful information where sequences have diverged over similar time scales.

The increased marker density provided by the integrated map is a valuable resource that increases the availability of markers in regions of interest, thus assisting in fine mapping. It also provides additional information for comparative mapping studies, e.g., to detect potential genome rearrangements in some populations. Furthermore, the increase in density of sequence tagged markers and availability of draft genome sequence scaffolds, enabled us to carry out a preliminary investigation of the relationship between genetic and physical distances in the *Brassica *A genome. This indicated that the chiasmata were not evenly distributed within chromosomes, and that there was considerable variation in the pattern of crossovers between chromosomes. Many studies have suggested the distribution of meiotic crossover events along chromosomes in plants and other species is non-random [62-66]. Non-random distributions of crossover rates have been reported to be correlated with several chromosomal features, including chromosome size, gene density, presence of transposable elements or heterochromatin, and distance to centromeres [67-72]. However, the underlying mechanisms affecting chiasmata distribution may be taxa specific [73], and so it is important to establish any relationships within or between *Brassica *chromosomes and species. Within the C genome of *B. oleracea*, a clear difference in relationship between genetic and physical distances has been established for IDBs on C6 [58]. The analysis we have carried out is preliminary and any mechanistic understanding will require more complete genome sequence scaffold data that include details of the distribution of repetitive DNA and of degree of chromatin condensation. In addition, it may be necessary to select additional markers that represent the full length of individual chromosomes. Based on complete genome sequence data, Drouaud *et al. *[74] have been able to resolve details of non-random distribution of chiasmata in relation to heterochromatic knobs and other chromosomal feature on *Arabidopsis *chromosome 4. Access to larger populations and more reliable sequence-tagged mapping methods (e.g., high-density SNP mapping) are likely to increase the resolution and understanding of the basis of variation in recombination frequency in *Brassica*.

We also attempted to anchor the remainder of the unanchored A genome scaffolds onto LGs based on the *B. napus *integrated map, and this anchored three additional scaffolds. Given the genome structure of *Brassica*, some scaffolds will be in repeat-rich or duplication regions, and thus it is difficult to resolve the LG assignments.

## Conclusions

In summary, we have generated a comprehensive integrated map for the *B. napus *genome, which includes 5,162 genetic markers mapped onto 2,196 loci, with a total genetic length of 1,792 cM. The map density of one locus every 0.82 cM, corresponding to 515 Kbp, increases by at least three-fold the marker density within the original maps. The BnaWAIT map thus provides access to additional informative markers, which will assist in resolution and fine mapping of QTL regions, as well as facilitating marker-assisted introgression and selection in *Brassica *crops. Our map integration pipeline is readily applied to map integration studies for other genera. The population-specific consolidated maps and the integrated maps are publicly available http://www.cropstoredb.org/brassica and provide a valuable resource in fine mapping and comparative mapping studies for *Brassica *research.

## Methods

### Component maps, genetic markers and genotype data

Three extensively studied *Brassica napus *mapping populations of doubled haploid (DH) lines, BnaSNDH, BnaDYDH and BnaSGDH (Additional File 8) were used to construct integrated maps. The BnaSNDH [7,16] and BnaDYDH [19,25] populations have been described previously. The BnaSGDH population was derived from an F1 generated from a cross between PSA12 (a resynthesized *B. napus *line generated from a cross between *B. oleracea *A12DHd and *B. rapa *Parkland Sunshine hybrid) and DH12075 (a DH line derived from a Westar × Cresor cross). All the mapping data (e.g., genetic maps and genotyping scoring matrices) of the three DH populations for the 19 linkage groups (LGs) have been collated and curated into the CropStoreDB database that provides a registry of data relating to *Brassica *genetics http://www.cropstoredb.org/brassica.

Assignment of marker loci to existing linkage groups was already available for a subset of previously published component maps (Additional File 8), BnaSNDH_02_2004a [20], BnaSNDH_03_2005a [7], BnaDYDH_01_2001a [19] and BnaDYDH_03_2008a [25]. These had been calculated using Mapmaker v3.0 [75,76], with LGs assigned at a threshold LOD score of > = 4.0. Similarly a component linkage map had been developed for BnaSGDH using a core set of RFLP markers. Additional SSR genotyping data for BnaSNDH and BnaSGDH (Additional File 8) were provisionally assigned to existing LGs by string-matching and linkage map distances were confirmed and calculated using Mapmaker v3.0. For each population, the composite sets of genotype data were pooled to generate a single matrix for each of the 19 linkage groups (LGs). Missing values (notated as "-") were assigned where a marker had not been genotyped for a particular individual line. Where scoring strings had been collated from more than one source for the same marker in the same population, the set containing the greater number of genotype scores was retained.

### Map construction

**Figure 5:**
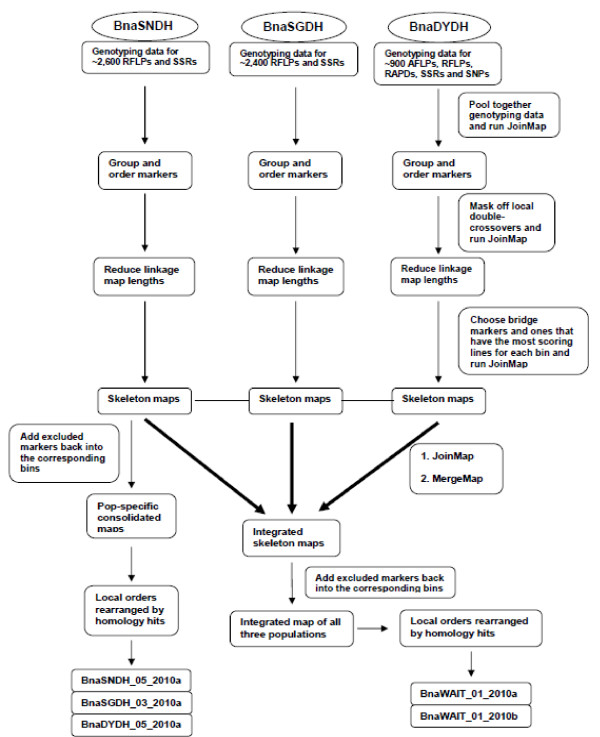
**Flow diagram indicating the process of the genetic map integration for three *B. napus *DH populations, BnaSNDH, BnaSGDH and BnaDYDH**.

The overall process of map integration is outlined in Figure 5. Each merged scoring matrix was analysed using JoinMap version 4.0 [48]. Linked loci were grouped with a LOD grouping threshold ranging from 3.0 to 5.0. Locus order within the LOD grouping was generated for each LG using the maximum likelihood (ML) algorithm with default parameters. The Kosambi map function was used to estimate genetic distances. Following initial ordering, the genotype matrix for each LG was investigated and data points were eliminated where a single locus was flanked by a double crossover. The modified genotype matrix for each LG was then imported again into JoinMap for linkage analysis using the same grouping and ordering algorithm and parameters. This procedure reduced the linkage map length for each LG in the integrated map by an average of >100 cM. Linkage groups were orientated consistent with Parkin *et al. *[7].

Prior to construction of an integrated map, population-specific bin maps were generated for each linkage group using a modification of the method described by Howad *et al. *[77]. A bin was defined where a unique map position was assigned. Thus, a bin may contain just one marker or more than one marker up to ~20 markers in our maps. Moreover, markers within <1 cM were also assigned to the same bin. A bin continued until a new map position was ≥1 cM distance from the first map position of the bin. The next bin would then start from the new map position. For each bin, a single genetic marker was selected that either provided a bridge to at least one other population-specific map, or maximised the information content with the maximum number of genotype/line scores. Following map calculation based on these binned genotype matrices, the residual markers were re-introduced and assigned to their bin positions.

We compared two different approaches for map integration, based on MergeMap and JoinMap procedures. In MergeMap [50], individual maps are first converted to directed acyclic graphs (DAG), which are merged into a consensus graph on the basis of their shared vertices. MergeMap then attempts to resolve conflicts among individual maps by deleting a minimum set of marker occurrences. The result of the conflict-resolution step is a consensus DAG, which is then simplified and linearised to produce the final consensus map.

JoinMap 4.0 [48] was used to generate pairwise recombination frequencies and LOD scores for the selected sets of representative loci for each linkage group, which were then combined into a single group node in the navigation tree. Within JoinMap the "Combine Groups for Map Integration" function carries out map calculations based on mean recombination frequencies and combined LOD scores [48]. The regression mapping algorithm was used and the LG lengths for the consensus map of all the representative markers were calculated. Values for the "jump" threshold ranged from 4.0 to 6.0. When more than ~150 markers are present, JoinMap is limited by computational constraints, as its computation time is the fourth power of the number of markers.

The final stage involved local rearrangement of marker order, where there was evidence of physical proximity based on homology to sequences co-located on contiguous stretches of DNA. Since this was primarily available for *Brassica rapa *BACs  http://www.brassica.info/resources.php, this evidence was strongly weighted to A genome LGs. In the absence of evidence from recombination (i.e., within the same map bins), the local order was sorted with the assumption of collinearity with *Arabidopsis*, based on the order of orthologous gene models and the previously described internal synteny block structure [7].

### Homology search between *Arabidopisis *and *Brassica rapa*

For each set of markers (Additional File 6 and 8) we identified the corresponding DNA sequences. This information has been collated and curated with the CropStoreDB and SeqStoreDB databases http://www.cropstoredb.org. SeqStoreDB contains records of all publicly available *Brassica *sequences released in GenBank, together with clone and primer sequences from many public and proprietary sources. This enables unambiguous management of sequence collections of query and target sequences, with explicit dataset versioning and recording of data provenance. The sequences associated with each set of genetic markers were used as queries in homology searches against the *Arabidopsis thaliana *pseudo-chromosomes (TAIR9 release, ftp://ftp.arabidopsis.org/home/tair/Sequences/), and against 1,089 sequenced *B. rapa *BACs available in NCBI GenBank (date version: 01/12/09). In addition, we were kindly provided with pre-publication access to 192 *B. rapa *Chiifu-401 genome scaffolds (255.9 Mb, representing 90% of the assembled sequences) by Xiaowu Wang, IVF-CAAS, Beijing. These scaffolds have been analysed and incorporated into the *Brassica rapa *Genome Sequencing Project Consortium [59]. These *Brassica *A genome scaffolds had been assigned to chromosomes based on integration of information from several different *B. rapa *genetic maps including BraCKDH [78], BraJWF3 [79] and BraVCS_DH http://www.brassica-rapa.org, as well as a newly constructed map for the BraRCZ16_DH population based on 86 SSRs and 403 InDel markers developed directly from the scaffold sequences. Where scaffolds could not be assigned and orientated with respect to *Brassica *A genome chromosomes by genetic markers, provisional locations were assigned based on location within collinearity blocks relative to *Arabidopsis *(The *Brassica rapa *Genome Sequencing Project Consortium [59]).

For RFLP probes, homology searches used the Tera-BLAST algorithm on a TimeLogic^R ^solutions DeCypher system http://www.timelogic.com/, with parameters: match = 1, mismatch = -3, gap open penalty = -5, gap extension penalty = -2, word size = 11 bp, and low complexity sequences filtered. A fairly low expect value (*E*-value) was used as the exclusion cutoff (1*E*-07). High-scoring alignment segments were then further excluded where (1) the sequence identity was less than 86% between *Brassica *and *Arabidopsis *(the average sequence identity over all aligned sequence pairs for RFLPs used in Parkin *et al. *[7]) and (2) alignment length was less than 100 consecutive nucleotides.

For the SSR markers, we used the whole clone sequences from which the original primer sequences had been designed. RepeatMasker http://www.repeatmasker.org/ was first used to mask simple repeats and interspersed repetitive elements from each SSR set. The algorithm of Cross_Match http://www.phrap.org was implemented and the *Brassicaceae *Repeat Database from TIGR plant repeat database http://plantrepeats.plantbiology.msu.edu/brassicaceae.html was used as the repeat library. Each masked SSR set was queried using the Tera-BLAST algorithm against target database sequences, using parameters: match = 1, mismatch = -1, gap open penalty = -2, gap extension penalty = -2, word size = 11 bp, with the dust filter on. The cutoff *E*-value of 1*E*-03 was used. We further excluded alignments where the sequence identity was less than 80% between *Brassica *and *Arabidopsis *and alignment length was less than 30 consecutive nucleotides. A similar approach has also been used to indentify homologous hits of microsatellite sequences between livestock species [80,81]. The sequence divergence cutoff value was increased to 90% for alignments between marker sequences derived from *B. napus *clones, and those of *B. rapa *BAC clones or genome scaffolds. This is a lower value than that suggested by the divergence between orthologous sequences of two stearoyl-ACP desaturase loci from the A genome of *B. rapa *and *B. napus*, which had 97.5% ± 3.1% sequence identity [82].

Where available, we also used SSR primer sequences (~20 bp in length) in pairs directly as query sequences to search for homologies against the *A. thaliana *pseudochromosomes, using the Tera-Probe algorithm http://www.timelogic.com/teraprobe.html with both gapped alignment and query filter options off. We allowed at most one mismatch between each of the primer sequences and the homologous *A. thaliana *sequences. Alignments were only accepted where both sequences from a primer pair had hits to the same *A. thaliana *chromosome, with the orientation consistent with the original conformation in *Brassica*, and the distance between the hits was shorter than 1000 bp and longer than 150 bp.

Homology search alignments were managed within the AlignStoreDB relational database. This enabled explicit and cumulative querying of result sets in the context of sets of markers located on specific linkage groups (managed within CropStoreDB). The relationships between the different databases are shown in Additional File 9.

The marker loci within the *Brassica *integrated map were compared with the chromosomal location of corresponding genes with the highest homology (in terms of bit scores) in the *Arabidopsis *genome and *B. rapa *genome scaffolds. Collinearity blocks were colour-coded according to the convention of Parkin *et al. *[7]. Positions of markers in the integrated maps are shown within each component map. We compared the marker order of the integrated map generated from the three populations and those of population-specific maps for each LG using dot plots. A dot was generated using a combination of a Perl script and the "conditional formatting" function within Microsoft Excel, and highlighted by linking the horizontal position in one map and the vertical position in the other map for a shared marker between the two maps. Such dot plots can be applied to compare marker orders for any pair of maps where there are shared markers. We then calculated Spearman's rank correlation coefficients for marker orders between pairs of maps.

## Authors' contributions

JW and GJK conceived the study. JW designed and performed the study, and wrote the paper. GJK designed and supervised the work, and wrote the paper. DJL, IAPP, CF and RD provided unpublished data and commented on the work. DJL contributed to editing the paper. PWCC curated the data. All authors read and approved the final manuscript.

## Supplementary Material

Additional file 1 **Comparison of marker orders between the three population-specific consolidated maps, generated by MapChart 2.1**. Click here for file

Additional file 2 **The BnaWAIT_01_2010a integrated map (by JoinMap) generated by MapChart 2.1**. Click here for file

Additional file 3 **All the information of high-scoring segement pairs (HSP) of canonical markers against Arabidopsis gene models, Arabidopsis chromosomes and B. rapa sequenced BACs**. The information of the BnaWAIT_10_2010a BnaWAIT map and markers aligned with *Arabidopsis *genes and chromosomes are also shown, so are the skeleton maps of each population. Click here for file

Additional file 4 **Dot-plots between the BnaWAIT map and all three population-specific maps for the remainder of all 19 LGs**. The marker order of the vertical axis is from the BnaWAIT_01_2010a integrated map, and three marker orders of the horizontal axis are for the three population-specific maps. Click here for file

Additional file 5 **Integrated skeleton maps of representative markers generated by JoinMap 4.0 Round 1 and Round 2 under the algorithm of regression**. The number of excluded representative markers from the map integration in these two rounds is reported. Spearman's rank correlation coefficients (r) between the two integrated maps (map1 and map2) and the three population-specific maps (BnaSNDH, BnaSGDH and BnaDYDH) are also shown. Click here for file

Additional file 6 **Number of different sets of canonical marker assays using in the *B. napus *mapping, number of marker assays that show homology with *Arabidopsis *and number of homology regions in *Arabidopsis *with similarity to *Brassica *canonical marker assays**. Click here for file

Additional file 7 **Summary of conserved collinearity blocks between the *B. napus *integrated map BnaWAIT_01_2010a and the *Arabidopsis *genome sequence**. Click here for file

Additional file 8 **Description of mapping populations and genetic markers used in the map integration study, which are maintained in CropStoreDB**. Corresponding references are also shown if available. Click here for file

Additional file 9 **Diagram of database interaction facilitating the map integration process and establishing links between genetic maps to DNA sequence information (e.g., TAIR9 genome or *B. rapa *BACs) via sequence-tagged marker sequences**. CropStoreDB is used to manage data relating to *Brassica *genetics, including populations, genetic maps, genetic markers and their positions. SeqStoreDB is used to manage all publicly available *Brassica *sequences together with sequence data from private sources. AlignStoreDB is used to manage all the homology alignments between query *Brassica *sequences and target genomic or BAC sequences. Click here for file
