# Supplementary material for: Integration of linkage maps for the Amphidiploid Brassica napus and comparative mapping with Arabidopsis and Brassica rapa
Source: BMC Genomics. 2011 Feb 9;12:101. doi: 10.1186/1471-2164-12-101 (PMC3042011; doi:10.1186/1471-2164-12-101)
Supplement: Additional file 1 — Comparison of marker orders between the three population-specific consolidated maps, generated by MapChart 2.1. [file 1471-2164-12-101-S1.PDF]

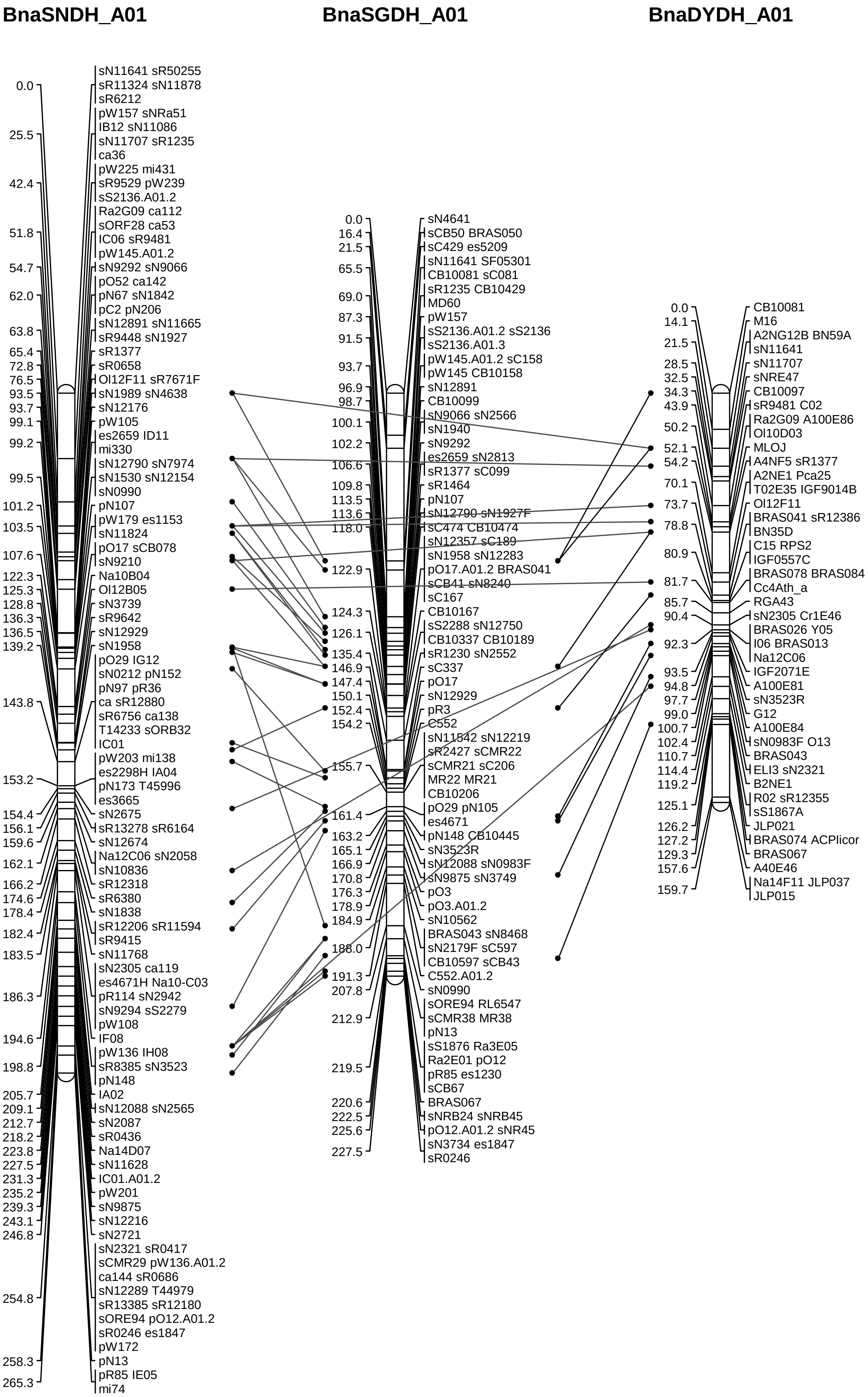
BnaSNDH_A01
0.0
25.5
42.4
51.8
54.7
62.0
63.8
65.4
72.8
76.5
93.5
93.7
99.1
99.2
99.5
101.2
103.5
107.6
122.3
125.3
128.8
136.3
136.5
139.2
143.8
153.2
154.4
156.1
159.6
162.1
166.2
174.6
178.4
182.4
183.5
186.3
194.6
198.8
205.7
209.1
212.7
218.2
223.8
227.5
231.3
235.2
239.3
243.1
246.8
254.8
258.3
265.3
sN11641 sR50255
sR11324 sN11878
sR6212
pW157 sNRa51
IB12 sN11086
sN11707 sR1235
ca36
pW225 mi431
sR9529 pW239
sS2136.A01.2
Ra2G09 ca112
sORF28 ca53
IC06 sR9481
pW145.A01.2
sN9292 sN9066
pO52 ca142
pN67 sN1842
pC2 pN206
sN12891 sN11665
sR9448 sN1927
sR1377
sR0658
Ol12F11 sR7671F
sN1989 sN4638
sN12176
pW105
es2659 ID11
mi330
sN12790 sN7974
sN1530 sN12154
sN0990
pN107
pW179 es1153
sN11824
pO17 sCB078
sN9210
Na10B04
Ol12B05
sN3739
sR9642
sN12929
sN1958
pO29 IG12
sN0212 pN152
pN97 pR36
ca sR12880
sR6756 ca138
T14233 sORB32
IC01
pW203 mi138
es2298H IA04
pN173 T45996
es3665
sN2675
sR13278 sR6164
sN12674
Na12C06 sN2058
sN10836
sR12318
sR6380
sN1838
sR12206 sR11594
sR9415
sN11768
sN2305 ca119
es4671H Na10-C03
pR114 sN2942
sN9294 sS2279
pW108
IF08
pW136 IH08
sR8385 sN3523
pN148
IA02
sN12088 sN2565
sN2087
sR0436
Na14D07
sN11628
IC01.A01.2
pW201
sN9875
sN12216
sN2721
sN2321 sR0417
sCMR29 pW136.A01.2
ca144 sR0686
sN12289 T44979
sR13385 sR12180
sORE94 pO12.A01.2
sR0246 es1847
pW172
pN13
pR85 IE05
mi74
BnaSGDH_A01
0.0
16.4
21.5
65.5
69.0
87.3
91.5
93.7
96.9
98.7
100.1
102.2
106.6
109.8
113.5
113.6
118.0
122.9
124.3
126.1
135.4
146.9
147.4
150.1
152.4
154.2
155.7
161.4
163.2
165.1
166.9
170.8
176.3
178.9
184.9
188.0
191.3
207.8
212.9
219.5
220.6
222.5
225.6
227.5
sN4641
sCB50 BRAS050
sC429 es5209
sN11641 SF05301
CB10081 sC081
sR1235 CB10429
MD60
pW157
sS2136.A01.2 sS2136
sS2136.A01.3
pW145.A01.2 sC158
pW145 CB10158
sN12891
CB10099
sN9066 sN2566
sN1940
sN9292
es2659 sN2813
sR1377 sC099
sR1464
pN107
sN12790 sN1927F
sC474 CB10474
sN12357 sC189
sN1958 sN12283
pO17.A01.2 BRAS041
sCB41 sN8240
sC167
CB10167
sS2288 sN12750
CB10337 CB10189
sR1230 sN2552
sC337
pO17
sN12929
pR3
C552
sN11542 sN12219
sR2427 sCMR22
sCMR21 sC206
MR22 MR21
CB10206
pO29 pN105
es4671
pN148 CB10445
sN3523R
sN12088 sN0983F
sN9875 sN3749
pO3
pO3.A01.2
sN10562
BRAS043 sN8468
sN2179F sC597
CB10597 sCB43
C552.A01.2
sN0990
sORE94 RL6547
sCMR38 MR38
pN13
sS1876 Ra3E05
Ra2E01 pO12
pR85 es1230
sCB67
BRAS067
sNRB24 sNRB45
pO12.A01.2 sNR45
sN3734 es1847
sR0246
BnaDYDH_A01
0.0
14.1
21.5
28.5
32.5
34.3
43.9
50.2
52.1
54.2
70.1
73.7
78.8
80.9
81.7
85.7
90.4
92.3
93.5
94.8
97.7
99.0
100.7
102.4
110.7
114.4
119.2
125.1
126.2
127.2
129.3
157.6
159.7
CB10081
M16
A2NG12B BN59A
sN11641
sN11707
sNRE47
CB10097
sR9481 C02
Ra2G09 A100E86
Ol10D03
MLOJ
A4NF5 sR1377
A2NE1 Pca25
T02E35 IGF9014B
Ol12F11
BRAS041 sR12386
BN35D
C15 RPS2
IGF0557C
BRAS078 BRAS084
Cc4Ath_a
RGA43
sN2305 Cr1E46
BRAS026 Y05
I06 BRAS013
Na12C06
IGF2071E
A100E81
sN3523R
G12
A100E84
sN0983F O13
BRAS043
ELI3 sN2321
B2NE1
R02 sR12355
sS1867A
JLP021
BRAS074 ACPlicor
BRAS067
A40E46
Na14F11 JLP037
JLP015

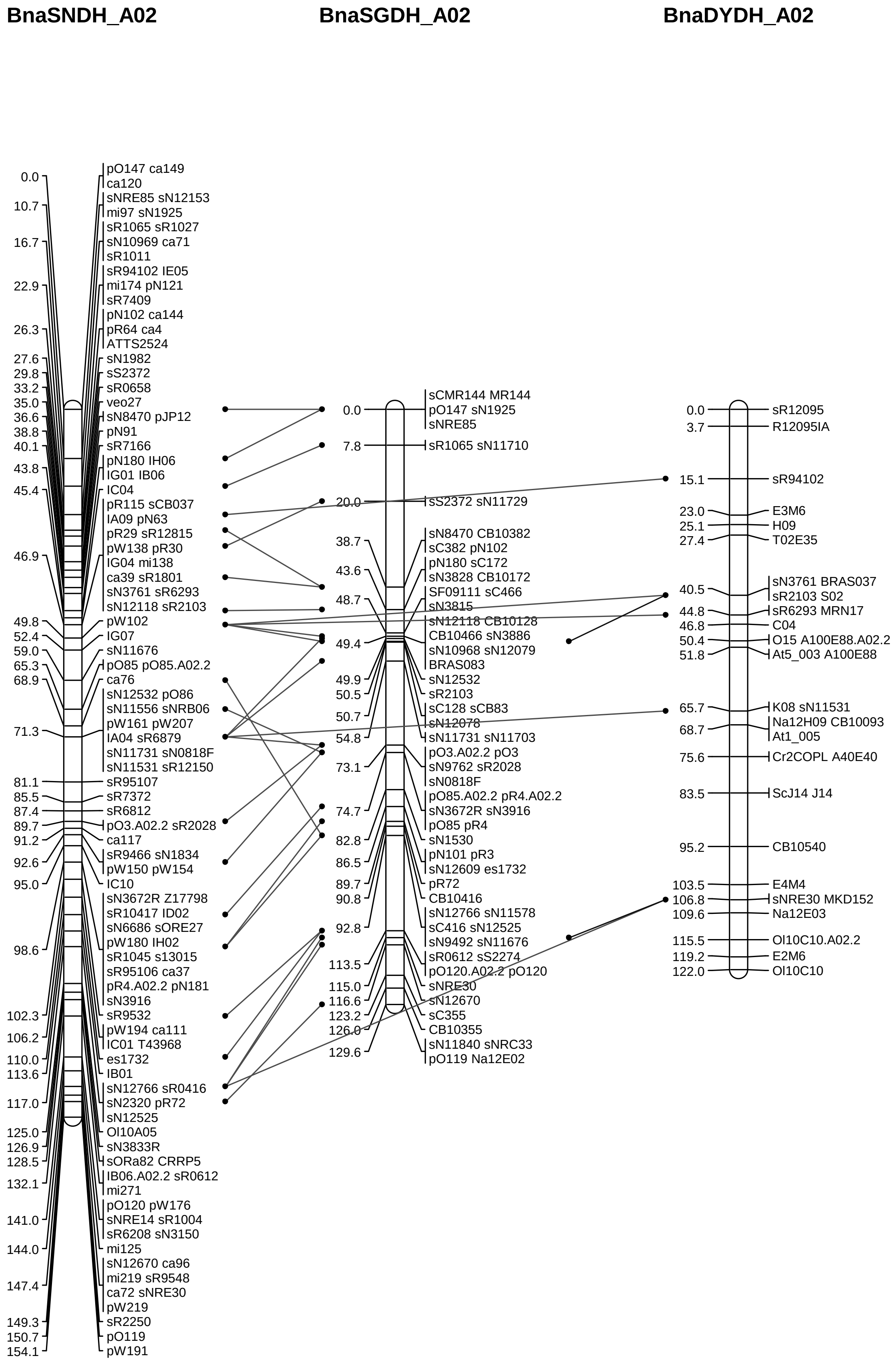
BnaSNDH_A02
BnaSGDH_A02
BnaDYDH_A02
0.0 pO147 ca149
ca120
10.7 sNRE85 sN12153
mi97 sN1925
16.7 sR1065 sR1027
sN10969 ca71
sR1011
22.9 sR94102 IE05
mi174 pN121
sR7409
26.3 pN102 ca144
pR64 ca4
ATTS2524
27.6 sN1982
29.8 sS2372
33.2 sR0658
35.0 veo27
36.6 sN8470 pJP12
38.8 pN91
40.1 sR7166
43.8 pN180 IH06
45.4 IG01 IB06
IC04
pR115 sCB037
IA09 pN63
pR29 sR12815
pW138 pR30
46.9 IG04 mi138
ca39 sR1801
sN3761 sR6293
sN12118 sR2103
49.8 pW102
52.4 IG07
59.0 sN11676
65.3 pO85 pO85.A02.2
68.9 ca76
sN12532 pO86
sN11556 sNRB06
71.3 pW161 pW207
IA04 sR6879
sN11731 sN0818F
sN11531 sR12150
81.1 sR95107
85.5 sR7372
87.4 sR6812
89.7 pO3.A02.2 sR2028
91.2 ca117
92.6 sR9466 sN1834
pW150 pW154
95.0 IC10
sN3672R Z17798
sR10417 ID02
sN6686 sORE27
98.6 pW180 IH02
sR1045 s13015
sR95106 ca37
pR4.A02.2 pN181
sN3916
102.3 sR9532
106.2 pW194 ca111
IC01 T43968
110.0 es1732
113.6 IB01
sN12766 sR0416
117.0 sN2320 pR72
sN12525
125.0 OI10A05
126.9 sN3833R
128.5 sORa82 CRRP5
132.1 IB06.A02.2 sR0612
mi271
pO120 pW176
141.0 sNRE14 sR1004
sR6208 sN3150
144.0 mi125
sN12670 ca96
147.4 mi219 sR9548
ca72 sNRE30
pW219
149.3 sR2250
150.7 pO119
154.1 pW191
0.0 sCMR144 MR144
pO147 sN1925
sNRE85
7.8 sR1065 sN11710
20.0 sS2372 sN11729
38.7 sN8470 CB10382
sC382 pN102
43.6 pN180 sC172
sN3828 CB10172
48.7 SF09111 sC466
sN3815
sN12118 CB10128
49.4 CB10466 sN3886
sN10968 sN12079
BRAS083
49.9 sN12532
50.5 sR2103
50.7 sC128 sCB83
sN12078
54.8 sN11731 sN11703
pO3.A02.2 pO3
73.1 sN9762 sR2028
sN0818F
pO85.A02.2 pR4.A02.2
74.7 sN3672R sN3916
pO85 pR4
82.8 sN1530
86.5 pN101 pR3
sN12609 es1732
89.7 pR72
90.8 CB10416
sN12766 sN11578
92.8 sC416 sN12525
sN9492 sN11676
113.5 sR0612 sS2274
115.0 pO120.A02.2 pO120
116.6 sNRE30
123.2 sN12670
126.0 sC355
CB10355
129.6 sN11840 sNRC33
pO119 Na12E02
0.0 sR12095
3.7 R12095IA
15.1 sR94102
23.0 E3M6
25.1 H09
27.4 T02E35
40.5 sN3761 BRAS037
sR2103 S02
44.8 sR6293 MRN17
46.8 C04
50.4 O15 A100E88.A02.2
51.8 At5_003 A100E88
65.7 K08 sN11531
68.7 Na12H09 CB10093
At1_005
75.6 Cr2COPL A40E40
83.5 ScJ14 J14
95.2 CB10540
103.5 E4M4
106.8 sNRE30 MKD152
109.6 Na12E03
115.5 Ol10C10.A02.2
119.2 E2M6
122.0 Ol10C10

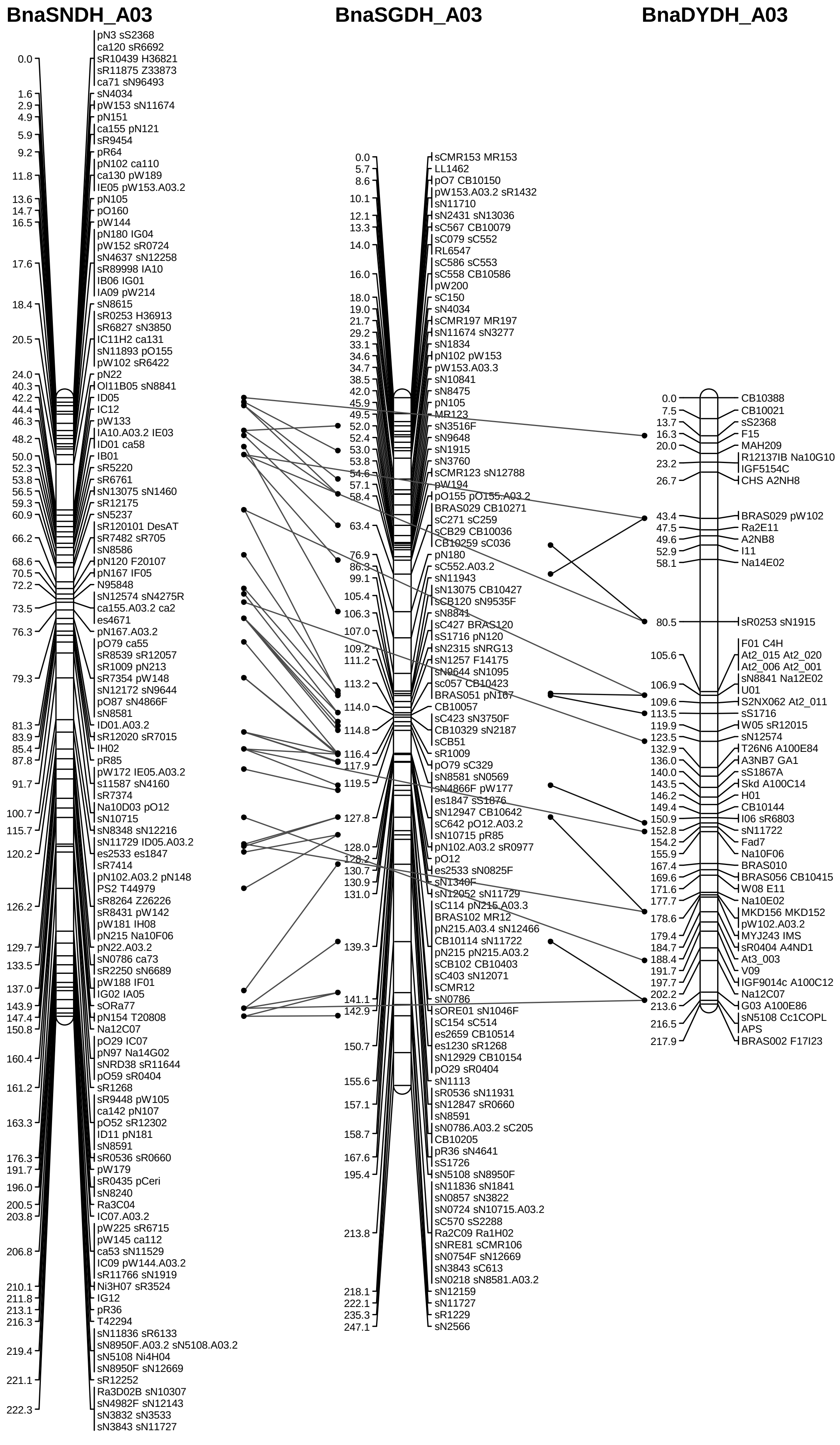
BnaSNDH_A03
BnaSGDH_A03
BnaDYDH_A03
pN3 sS2368
ca120 sR6692
sR10439 H36821
sR11875 Z33873
ca71 sN96493
sN4034
pW153 sN11674
pN151
ca155 pN121
sR9454
pR64
pN102 ca110
ca130 pW189
IE05 pW153.A03.2
pN105
pO160
pW144
pN180 IG04
pW152 sR0724
sN4637 sN12258
sR89998 IA10
IB06 IG01
IA09 pW214
sN8615
sR0253 H36913
sR6827 sN3850
IC11H2 ca131
sN11893 pO155
pW102 sR6422
pN22
OI11B05 sN8841
ID05
IC12
pW133
IA10.A03.2 IE03
ID01 ca58
IB01
sR5220
sR6761
sN13075 sN1460
sR12175
sN5237
sR120101 DesAT
sR7482 sR705
sN8586
pN120 F20107
pN167 IF05
N95848
sN12574 sN4275R
ca155.A03.2 ca2
es4671
pN167.A03.2
pO79 ca55
sR8539 sR12057
sR1009 pN213
sR7354 pW148
sN12172 sN9644
pO87 sN4866F
sN8581
ID01.A03.2
sR12020 sR7015
IH02
pR85
pW172 IE05.A03.2
s11587 sN4160
sR7374
Na10D03 pO12
sN10715
sN8348 sN12216
sN11729 ID05.A03.2
es2533 es1847
sR7414
pN102.A03.2 pN148
PS2 T44979
sR8264 Z26226
sR8431 pW142
pW181 IH08
pN215 Na10F06
pN22.A03.2
sN0786 ca73
sR2250 sN6689
pW188 IF01
IG02 IA05
sORa77
pN154 T20808
Na12C07
pO29 IC07
pN97 Na14G02
sNRD38 sR11644
pO59 sR0404
sR1268
sR9448 pW105
ca142 pN107
pO52 sR12302
ID11 pN181
sN8591
sR0536 sR0660
pW179
sR0435 pCeri
sN8240
Ra3C04
IC07.A03.2
pW225 sR6715
pW145 ca112
ca53 sN11529
IC09 pW144.A03.2
sR11766 sN1919
Ni3H07 sR3524
IG12
pR36
T42294
sN11836 sR6133
sN8950F.A03.2 sN5108.A03.2
sN5108 Ni4H04
sN8950F sN12669
sR12252
Ra3D02B sN10307
sN4982F sN12143
sN3832 sN3533
sN3843 sN11727
sCMR153 MR153
LL1462
pO7 CB10150
pW153.A03.2 sR1432
sN11710
sN2431 sN13036
sC567 CB10079
sC079 sC552
RL6547
sC586 sC553
sC558 CB10586
pW200
sC150
sN4034
sCMR197 MR197
sN11674 sN3277
sN1834
pN102 pW153
pW153.A03.3
sN10841
sN8475
pN105
MR123
sN3516F
sN9648
sN1915
sN3760
sCMR123 sN12788
pW194
pO155 pO155.A03.2
BRAS029 CB10271
sC271 sC259
sCB29 CB10036
CB10259 sC036
pN180
sC552.A03.2
sN11943
sN13075 CB10427
sCB120 sN9535F
sN8841
sC427 BRAS120
sS1716 pN120
sN2315 sNRG13
sN1257 F14175
sN9644 sN1095
sc057 CB10423
BRAS051 pN167
CB10057
sC423 sN3750F
CB10329 sN2187
sCB51
sR1009
pO79 sC329
sN8581 sN0569
sN4866F pW177
es1847 sS1876
sN12947 CB10642
sC642 pO12.A03.2
sN10715 pR85
pN102.A03.2 sR0977
pO12
es2533 sN0825F
sN1340F
sN12052 sN11729
sC114 pN215.A03.3
BRAS102 MR12
pN215.A03.4 sN12466
CB10114 sN11722
pN215 pN215.A03.2
sCB102 CB10403
sC403 sN12071
sCMR12
sN0786
sORE01 sN1046F
sC154 sC514
es2659 CB10514
es1230 sR1268
sN12929 CB10154
pO29 sR0404
sN1113
sR0536 sN11931
sN12847 sR0660
sN8591
sN0786.A03.2 sC205
CB10205
pR36 sN4641
sS1726
sN5108 sN8950F
sN11836 sN1841
sN0857 sN3822
sN0724 sN10715.A03.2
sC570 sS2288
Ra2C09 Ra1H02
sNRE81 sCMR106
sN0754F sN12669
sN3843 sC613
sN0218 sN8581.A03.2
sN12159
sN11727
sR1229
sN2566
CB10388
CB10021
sS2368
F15
MAH209
R12137IB Na10G10
IGF5154C
CHS A2NH8
BRAS029 pW102
Ra2E11
A2NB8
I11
Na14E02
sR0253 sN1915
F01 C4H
At2_015 At2_020
At2_006 At2_001
sN8841 Na12E02
U01
S2NX062 At2_011
sS1716
W05 sR12015
sN12574
T26N6 A100E84
A3NB7 GA1
sS1867A
Skd A100C14
H01
CB10144
I06 sR6803
sN11722
Fad7
Na10F06
BRAS010
BRAS056 CB10415
W08 E11
Na10E02
MKD156 MKD152
pW102.A03.2
MYJ243 IMS
sR0404 A4ND1
At3_003
V09
IGF9014c A100C12
Na12C07
G03 A100E86
sN5108 Cc1COPL
APS
BRAS002 F17I23

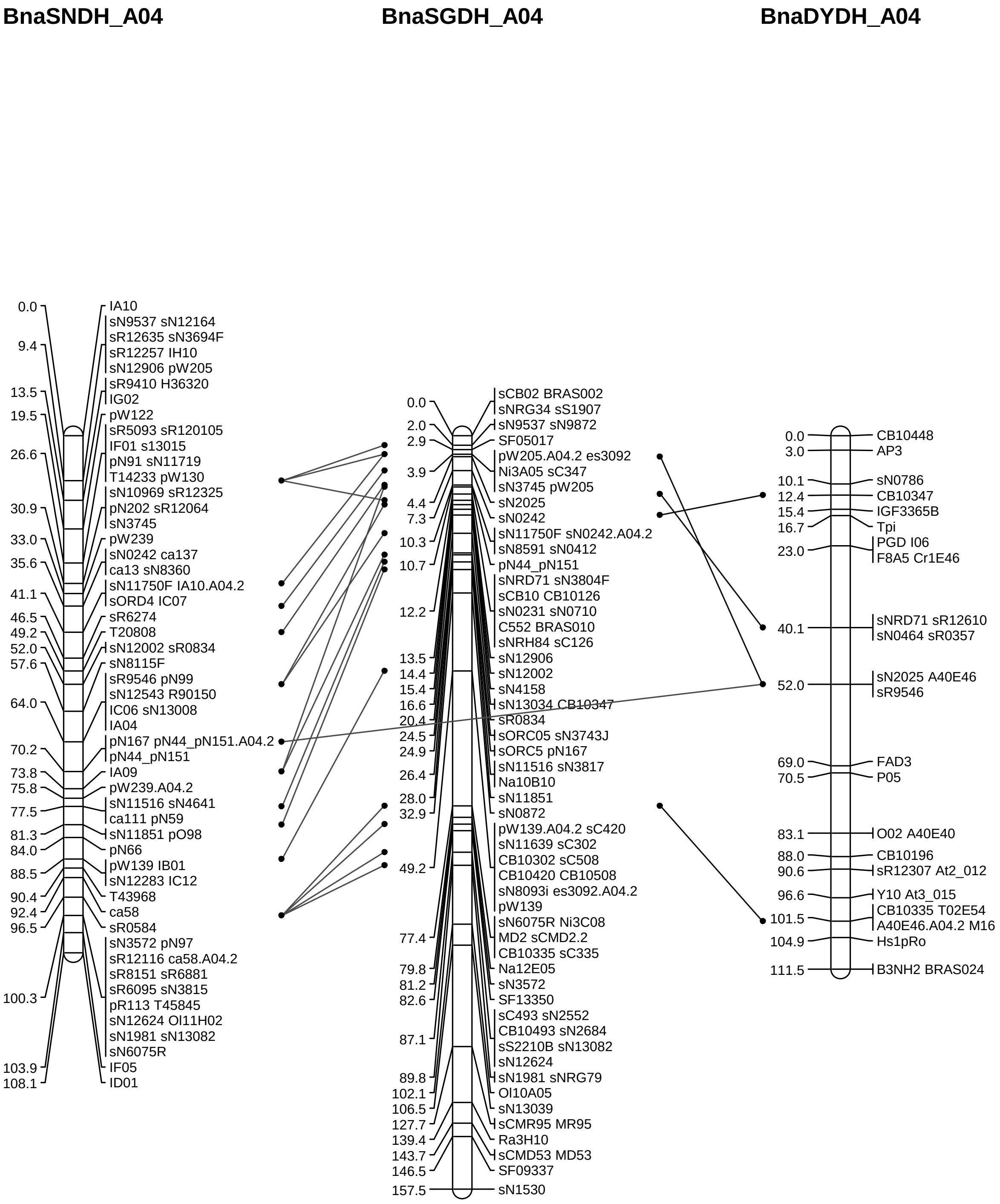
BnaSNDH_A04
BnaSGDH_A04
BnaDYDH_A04
0.0 IA10
sN9537 sN12164
9.4 sR12635 sN3694F
sR12257 IH10
sN12906 pW205
13.5 sR9410 H36320
IG02
19.5 pW122
sR5093 sR120105
IF01 s13015
26.6 pN91 sN11719
T14233 pW130
sN10969 sR12325
30.9 pN202 sR12064
sN3745
33.0 pW239
35.6 sN0242 ca137
ca13 sN8360
41.1 sN11750F IA10.A04.2
sORD4 IC07
46.5 sR6274
49.2 T20808
52.0 sN12002 sR0834
57.6 sN8115F
sR9546 pN99
64.0 sN12543 R90150
IC06 sN13008
IA04
70.2 pN167 pN44_pN151.A04.2
pN44_pN151
73.8 IA09
75.8 pW239.A04.2
77.5 sN11516 sN4641
ca111 pN59
81.3 sN11851 pO98
84.0 pN66
88.5 pW139 IB01
sN12283 IC12
90.4 T43968
92.4 ca58
96.5 sR0584
sN3572 pN97
sR12116 ca58.A04.2
sR8151 sR6881
100.3 sR6095 sN3815
pR113 T45845
sN12624 OI11H02
sN1981 sN13082
sN6075R
103.9 IF05
108.1 ID01
0.0 sCB02 BRAS002
sNRG34 sS1907
2.0 sN9537 sN9872
2.9 SF05017
pW205.A04.2 es3092
3.9 Ni3A05 sC347
sN3745 pW205
4.4 sN2025
7.3 sN0242
sN11750F sN0242.A04.2
10.3 sN8591 sN0412
10.7 pN44_pN151
sNRD71 sN3804F
sCB10 sCB10126
12.2 sN0231 sN0710
C552 BRAS010
sNRH84 sC126
13.5 sN12906
14.4 sN12002
15.4 sN4158
16.6 sN13034 CB10347
20.4 sR0834
24.5 sORC05 sN3743J
24.9 sORC5 pN167
26.4 sN11516 sN3817
Na10B10
28.0 sN11851
32.9 sN0872
pW139.A04.2 sC420
sN11639 sC302
CB10302 sC508
49.2 CB10420 CB10508
sN8093i es3092.A04.2
pW139
sN6075R Ni3C08
77.4 MD2 sCMD2.2
CB10335 sC335
79.8 Na12E05
81.2 sN3572
82.6 SF13350
sC493 sN2552
CB10493 sN2684
87.1 sS2210B sN13082
sN12624
89.8 sN1981 sNRG79
102.1 OI10A05
106.5 sN13039
127.7 sCMR95 MR95
139.4 Ra3H10
143.7 sCMD53 MD53
146.5 SF09337
157.5 sN1530
0.0 CB10448
3.0 AP3
10.1 sN0786
12.4 CB10347
15.4 IGF3365B
16.7 Tpi
23.0 PGD I06
F8A5 Cr1E46
40.1 sNRD71 sR12610
sN0464 sR0357
52.0 sN2025 A40E46
sR9546
69.0 FAD3
70.5 P05
83.1 O02 A40E40
88.0 CB10196
90.6 sR12307 At2_012
96.6 Y10 At3_015
101.5 CB10335 T02E54
A40E46.A04.2 M16
104.9 Hs1pRo
111.5 B3NH2 BRAS024

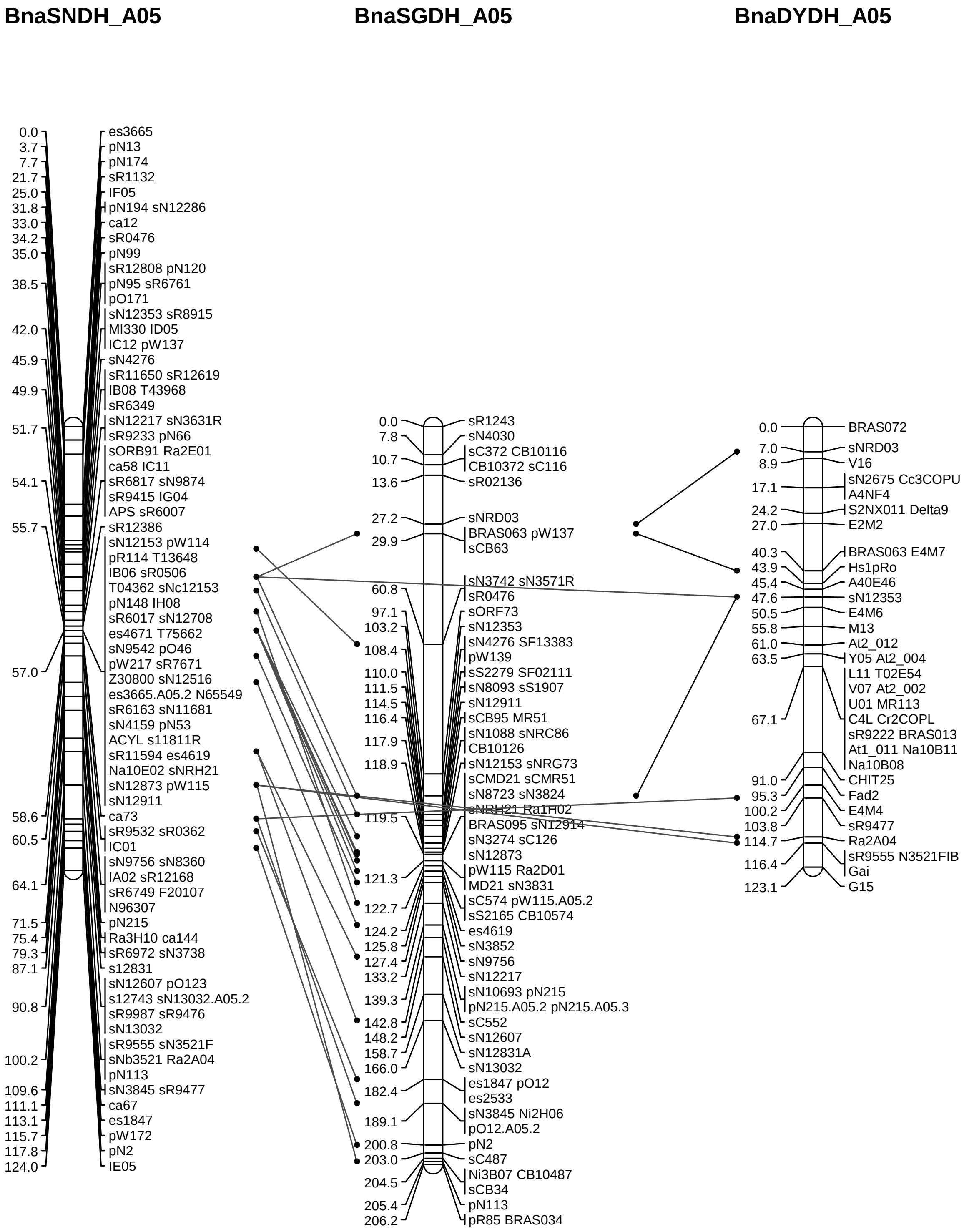
BnaSNDH_A05
BnaSGDH_A05
BnaDYDH_A05
0.0 es3665
3.7 pN13
7.7 pN174
21.7 sR1132
25.0 IF05
31.8 pN194 sN12286
33.0 ca12
34.2 sR0476
35.0 pN99
sR12808 pN120
38.5 pN95 sR6761
pO171
sN12353 sR8915
42.0 MI330 ID05
IC12 pW137
45.9 sN4276
sR11650 sR12619
49.9 IB08 T43968
sR6349
51.7 sN12217 sN3631R
sR9233 pN66
sORB91 Ra2E01
ca58 IC11
54.1 sR6817 sN9874
sR9415 IG04
APS sR6007
55.7 sR12386
sN12153 pW114
pR114 T13648
IB06 sR0506
T04362 sNc12153
pN148 IH08
sR6017 sN12708
es4671 T75662
sN9542 pO46
pW217 sR7671
57.0 Z30800 sN12516
es3665.A05.2 N65549
sR6163 sN11681
sN4159 pN53
ACYL s11811R
sR11594 es4619
Na10E02 sNRH21
sN12873 pW115
sN12911
58.6 ca73
60.5 sR9532 sR0362
IC01
sN9756 sN8360
64.1 IA02 sR12168
sR6749 F20107
N96307
71.5 pN215
75.4 Ra3H10 ca144
79.3 sR6972 sN3738
87.1 s12831
sN12607 pO123
90.8 s12743 sN13032.A05.2
sR9987 sR9476
sN13032
sR9555 sN3521F
100.2 sNb3521 Ra2A04
pN113
109.6 sN3845 sR9477
111.1 ca67
113.1 es1847
115.7 pW172
117.8 pN2
124.0 IE05
0.0 sR1243
7.8 sN4030
10.7 sC372 CB10116
CB10372 sC116
13.6 sR02136
27.2 sNRD03
29.9 BRAS063 pW137
sCB63
sN3742 sN3571R
60.8 sR0476
97.1 sORF73
103.2 sN12353
108.4 sN4276 SF13383
pW139
110.0 sS2279 SF02111
111.5 sN8093 sS1907
114.5 sN12911
116.4 sCB95 MR51
117.9 sN1088 sNRC86
CB10126
118.9 sN12153 sNRG73
sCMD21 sCMR51
sN8723 sN3824
sNRH21 Ra1H02
119.5 BRAS095 sN12914
sN3274 sC126
sN12873
121.3 pW115 Ra2D01
MD21 sN3831
122.7 sC574 pW115.A05.2
sS2165 CB10574
124.2 es4619
125.8 sN3852
127.4 sN9756
133.2 sN12217
139.3 sN10693 pN215
pN215.A05.2 pN215.A05.3
142.8 sC552
148.2 sN12607
158.7 sN12831A
166.0 sN13032
182.4 es1847 pO12
es2533
189.1 sN3845 Ni2H06
pO12.A05.2
200.8 pN2
203.0 sC487
204.5 Ni3B07 CB10487
sCB34
205.4 pN113
206.2 pR85 BRAS034
0.0 BRAS072
7.0 sNRD03
8.9 V16
17.1 sN2675 Cc3COPU
A4NF4
24.2 S2NX011 Delta9
27.0 E2M2
40.3 BRAS063 E4M7
43.9 Hs1pRo
45.4 A40E46
47.6 sN12353
50.5 E4M6
55.8 M13
61.0 At2_012
63.5 Y05 At2_004
L11 T02E54
V07 At2_002
U01 MR113
67.1 C4L Cr2COPL
sR9222 BRAS013
At1_011 Na10B11
Na10B08
91.0 CHIT25
95.3 Fad2
100.2 E4M4
103.8 sR9477
114.7 Ra2A04
116.4 sR9555 N3521FIB
Gai
123.1 G15

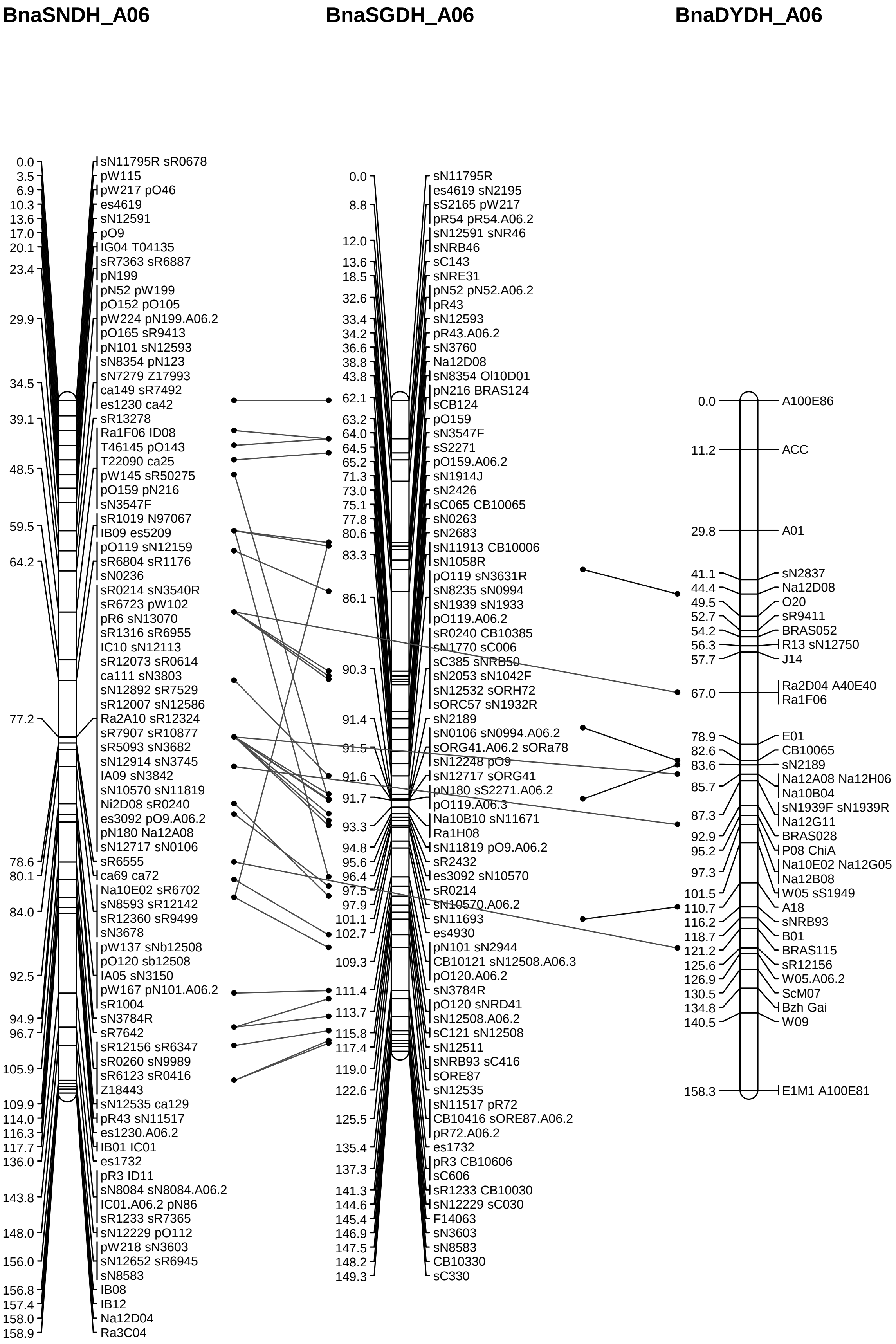
BnaSNDH_A06
BnaSGDH_A06
BnaDYDH_A06
0.0 3.5 6.9 10.3 13.6 17.0 20.1 23.4 29.9 34.5 39.1 48.5 59.5 64.2 77.2 78.6 80.1 84.0 92.5 94.9 96.7 105.9 109.9 114.0 116.3 117.7 136.0 143.8 148.0 156.0 156.8 157.4 158.0 158.9
sN11795R sR0678
pW115
pW217 pO46
es4619
sN12591
pO9
IG04 T04135
sR7363 sR6887
pN199
pN52 pW199
pO152 pO105
pW224 pN199.A06.2
pO165 sR9413
pN101 sN12593
sN8354 pN123
sN7279 Z17993
ca149 sR7492
es1230 ca42
sR13278
Ra1F06 ID08
T46145 pO143
T22090 ca25
pW145 sR50275
pO159 pN216
sN3547F
sR1019 N97067
IB09 es5209
pO119 sN12159
sR6804 sR1176
sN0236
sR0214 sN3540R
sR6723 pW102
pR6 sN13070
sR1316 sR6955
IC10 sN12113
sR12073 sR0614
ca111 sN3803
sN12892 sR7529
sR12007 sN12586
Ra2A10 sR12324
sR7907 sR10877
sR5093 sN3682
sN12914 sR3745
IA09 sN3842
sN10570 sN11819
Ni2D08 sR0240
es3092 pO9.A06.2
pN180 Na12A08
sN12717 sN0106
sR6555
ca69 ca72
Na10E02 sR6702
sN8593 sR12142
sR12360 sR9499
sN3678
pW137 sNb12508
pO120 sb12508
IA05 sN3150
pW167 pN101.A06.2
sR1004
sN3784R
sR7642
sR12156 sR6347
sR0260 sN9989
sR6123 sR0416
Z18443
sN12535 ca129
pR43 sN11517
es1230.A06.2
IB01 IC01
es1732
pR3 ID11
sN8084 sN8084.A06.2
IC01.A06.2 pN86
sR1233 sR7365
sN12229 pO112
pW218 sN3603
sN12652 sR6945
sN8583
IB08
IB12
Na12D04
Ra3C04
0.0 8.8 12.0 13.6 18.5 32.6 33.4 34.2 36.6 38.8 43.8 62.1 63.2 64.0 64.5 65.2 71.3 73.0 75.1 77.8 80.6 83.3 86.1 90.3 91.4 91.5 91.6 91.7 93.3 94.8 95.6 96.4 97.5 97.9 101.1 102.7 109.3 111.4 113.7 115.8 117.4 119.0 122.6 125.5 135.4 137.3 141.3 144.6 145.4 146.9 147.5 148.2 149.3
sN11795R
es4619 sN2195
sS2165 pW217
pR54 pR54.A06.2
sN12591 sNR46
sNRB46
sC143
sNRE31
pN52 pN52.A06.2
pR43
sN12593
pR43.A06.2
sN3760
Na12D08
sN8354 Ol10D01
pN216 BRAS124
sCB124
pO159
sN3547F
sS2271
pO159.A06.2
sN1914J
sN2426
sC065 CB10065
sN0263
sN2683
sN11913 CB10006
sN1058R
pO119 sN3631R
sN8235 sN0994
sN1939 sN1933
pO119.A06.2
sR0240 CB10385
sN1770 sC006
sC385 sNRB50
sN2053 sN1042F
sN12532 sORH72
sORC57 sN1932R
sN2189
sN0106 sN0994.A06.2
sORG41.A06.2 sORa78
sN12248 pO9
sN12717 sORG41
pN180 sS2271.A06.2
pO119.A06.3
Na10B10 sN11671
Ra1H08
sN11819 pO9.A06.2
sR2432
es3092 sN10570
sR0214
sN10570.A06.2
sN11693
es4930
pN101 sN2944
CB10121 sN12508.A06.3
pO120.A06.2
sN3784R
pO120 sNRD41
sN12508.A06.2
sC121 sN12508
sN12511
sNRB93 sC416
sORE87
sN12535
sN11517 pR72
CB10416 sORE87.A06.2
pR72.A06.2
es1732
pR3 CB10606
sC606
sR1233 CB10030
sN12229 sC030
F14063
sN3603
sN8583
CB10330
sC330
0.0 11.2 29.8 41.1 44.4 49.5 52.7 54.2 56.3 57.7 67.0 78.9 82.6 83.6 85.7 87.3 92.9 95.2 97.3 101.5 110.7 116.2 118.7 121.2 125.6 126.9 130.5 134.8 140.5 158.3
A100E86
ACC
A01
sN2837
Na12D08
O20
sR9411
BRAS052
R13 sN12750
J14
Ra2D04 A40E40
Ra1F06
E01
CB10065
sN2189
Na12A08 Na12H06
Na10B04
sN1939F sN1939R
Na12G11
BRAS028
P08 ChiA
Na10E02 Na12G05
Na12B08
W05 sS1949
A18
sNRB93
B01
BRAS115
sR12156
W05.A06.2
ScM07
Bzh Gai
W09
E1M1 A100E81

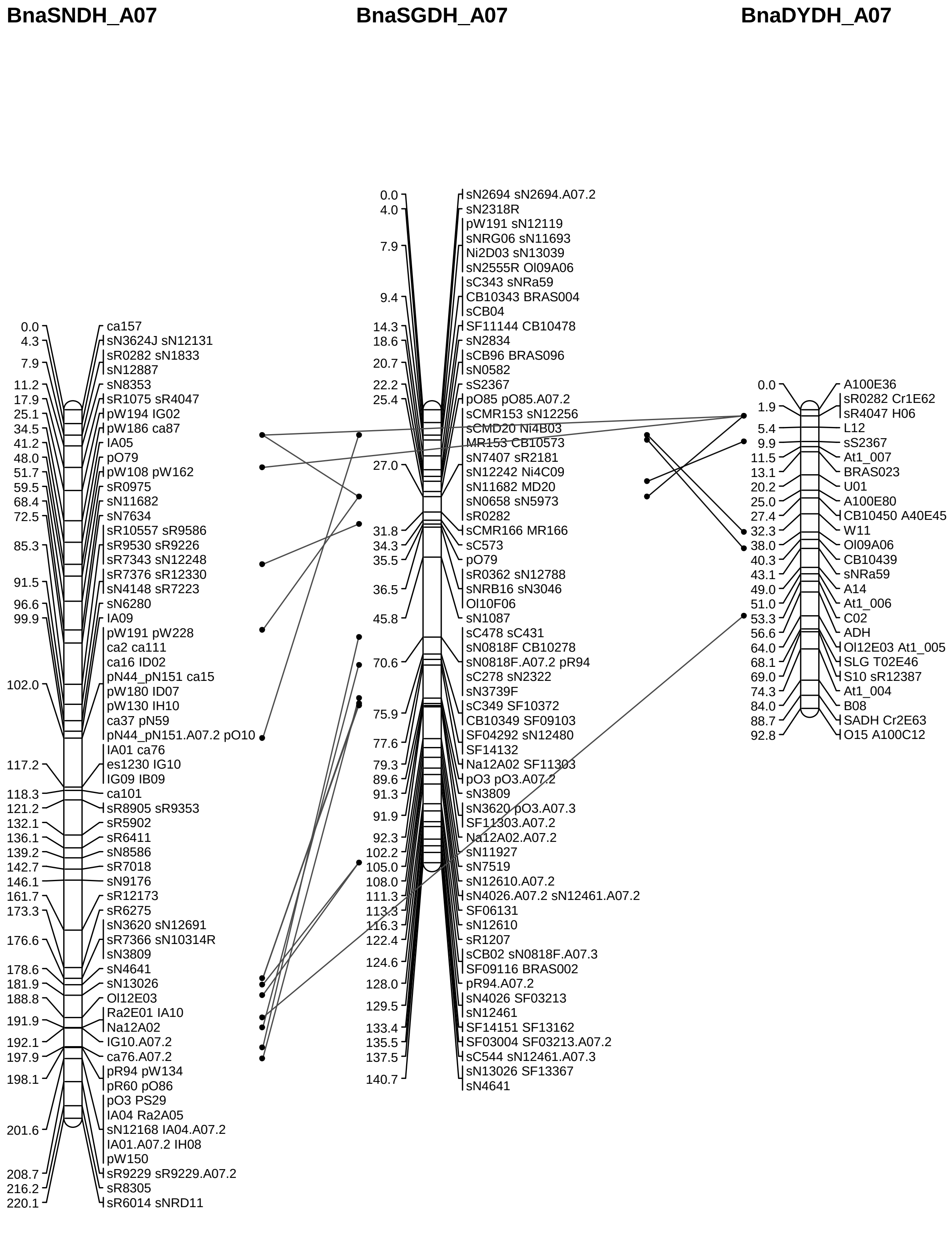
BnaSNDH_A07
BnaSGDH_A07
BnaDYDH_A07
0.0 ca157
4.3 sN3624J sN12131
7.9 sR0282 sN1833
sN12887
11.2 sN8353
17.9 sR1075 sR4047
25.1 pW194 IG02
34.5 pW186 ca87
41.2 IA05
48.0 pO79
51.7 pW108 pW162
59.5 sR0975
68.4 sN11682
72.5 sN7634
sR10557 sR9586
85.3 sR9530 sR9226
sR7343 sN12248
sR7376 sR12330
91.5 sN4148 sR7223
96.6 sN6280
99.9 IA09
pW191 pW228
ca2 ca111
ca16 ID02
102.0 pN44_pN151 ca15
pW180 ID07
pW130 IH10
ca37 pN59
pN44_pN151.A07.2 pO10
IA01 ca76
117.2 es1230 IG10
IG09 IB09
118.3 ca101
121.2 sR8905 sR9353
132.1 sR5902
136.1 sR6411
139.2 sN8586
142.7 sR7018
146.1 sN9176
161.7 sR12173
173.3 sR6275
sN3620 sN12691
176.6 sR7366 sN10314R
sN3809
178.6 sN4641
181.9 sN13026
188.8 Ol12E03
191.9 Ra2E01 IA10
Na12A02
192.1 IG10.A07.2
197.9 ca76.A07.2
198.1 pR94 pW134
pR60 pO86
pO3 PS29
IA04 Ra2A05
201.6 sN12168 IA04.A07.2
IA01.A07.2 IH08
pW150
208.7 sR9229 sR9229.A07.2
216.2 sR8305
220.1 sR6014 sNRD11
0.0 sN2694 sN2694.A07.2
4.0 sN2318R
pW191 sN12119
7.9 sNRG06 sN11693
Ni2D03 sN13039
sN2555R Ol09A06
sC343 sNRa59
9.4 CB10343 BRAS004
sCB04
14.3 SF11144 CB10478
18.6 sN2834
20.7 sCB96 BRAS096
sN0582
22.2 sS2367
25.4 pO85 pO85.A07.2
sCMR153 sN12256
sCMD20 Ni4B03
MR153 CB10573
27.0 sN7407 sR2181
sN12242 Ni4C09
sN11682 MD20
sN0658 sN5973
sR0282
31.8 sCMR166 MR166
34.3 sC573
35.5 pO79
36.5 sR0362 sN12788
sNRB16 sN3046
Ol10F06
45.8 sN1087
sC478 sC431
sN0818F CB10278
70.6 sN0818F.A07.2 pR94
sC278 sN2322
sN3739F
75.9 sC349 SF10372
CB10349 SF09103
77.6 SF04292 sN12480
SF14132
79.3 Na12A02 SF11303
89.6 pO3 pO3.A07.2
91.3 sN3809
91.9 sN3620 pO3.A07.3
SF11303.A07.2
92.3 Na12A02.A07.2
102.2 sN11927
105.0 sN7519
108.0 sN12610.A07.2
111.3 sN4026.A07.2 sN12461.A07.2
113.3 SF06131
116.3 sN12610
122.4 sR1207
124.6 sCB02 sN0818F.A07.3
SF09116 BRAS002
128.0 pR94.A07.2
129.5 sN4026 SF03213
sN12461
133.4 SF14151 SF13162
135.5 SF03004 SF03213.A07.2
137.5 sC544 sN12461.A07.3
140.7 sN13026 SF13367
sN4641
0.0 A100E36
1.9 sR0282 Cr1E62
sR4047 H06
5.4 L12
9.9 sS2367
11.5 At1_007
13.1 BRAS023
20.2 U01
25.0 A100E80
27.4 CB10450 A40E45
32.3 W11
38.0 Ol09A06
40.3 CB10439
43.1 sNRa59
49.0 A14
51.0 At1_006
53.3 C02
56.6 ADH
64.0 Ol12E03 At1_005
68.1 SLG T02E46
69.0 S10 sR12387
74.3 At1_004
84.0 B08
88.7 SADH Cr2E63
92.8 O15 A100C12

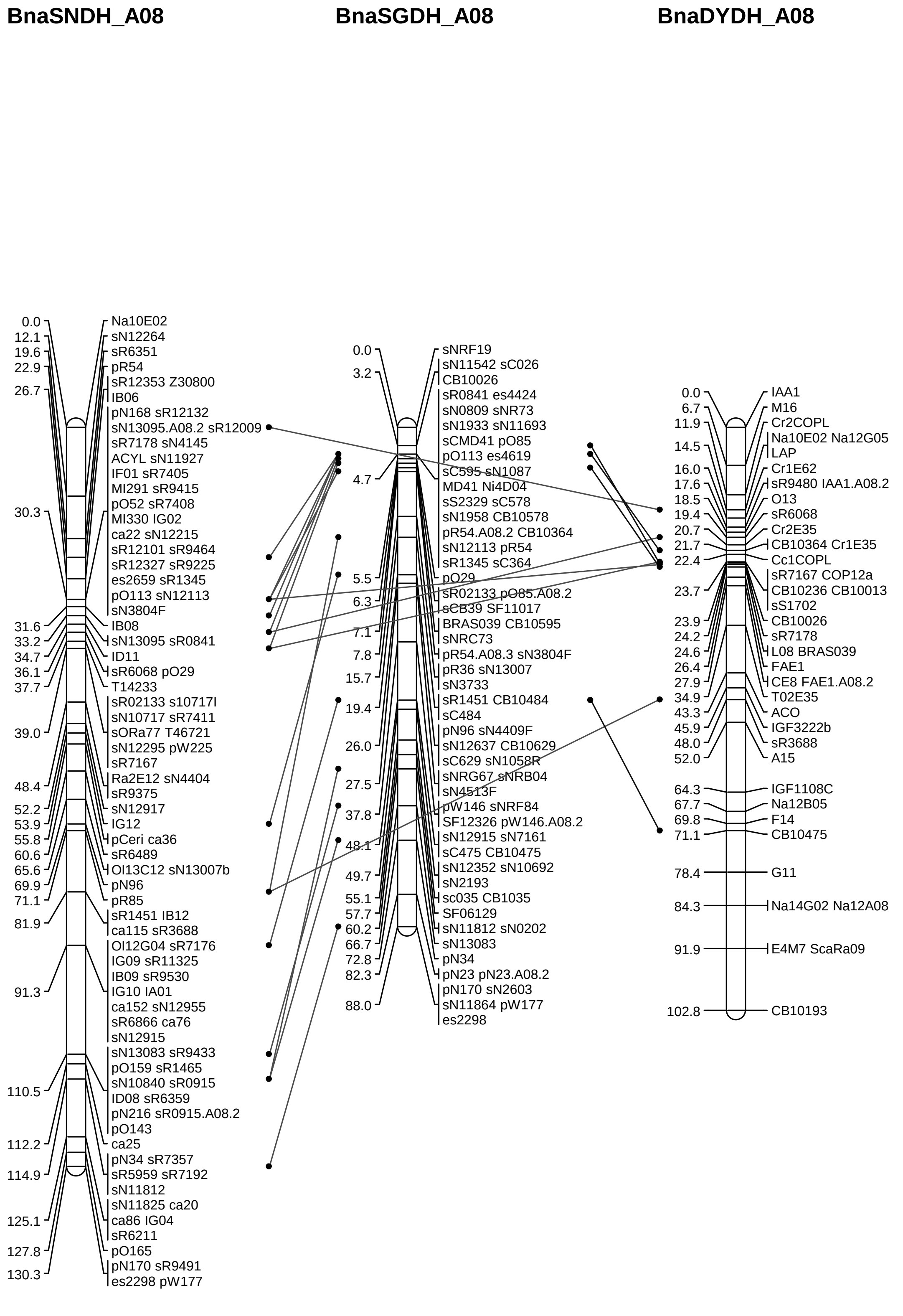
BnaSNDH_A08
BnaSGDH_A08
BnaDYDH_A08
0.0
12.1
19.6
22.9
26.7
30.3
31.6
33.2
34.7
36.1
37.7
39.0
48.4
52.2
53.9
55.8
60.6
65.6
69.9
71.1
81.9
91.3
110.5
112.2
114.9
125.1
127.8
130.3
Na10E02
sN12264
sR6351
pR54
sR12353 Z30800
IB06
pN168 sR12132
sN13095.A08.2 sR12009
sR7178 sN4145
ACYL sN11927
IF01 sR7405
MI291 sR9415
pO52 sR7408
MI330 IG02
ca22 sN12215
sR12101 sR9464
sR12327 sR9225
es2659 sR1345
pO113 sN12113
sN3804F
IB08
sN13095 sR0841
ID11
sR6068 pO29
T14233
sR02133 s10717I
sN10717 sR7411
sORa77 T46721
sN12295 pW225
sR7167
Ra2E12 sN4404
sR9375
sN12917
IG12
pCeri ca36
sR6489
OI13C12 sN13007b
pN96
pR85
sR1451 IB12
ca115 sR3688
OI12G04 sR7176
IG09 sR11325
IB09 sR9530
IG10 IA01
ca152 sN12955
sR6866 ca76
sN12915
sN13083 sR9433
pO159 sR1465
sN10840 sR0915
ID08 sR6359
pN216 sR0915.A08.2
pO143
ca25
pN34 sR7357
sR5959 sR7192
sN11812
sN11825 ca20
ca86 IG04
sR6211
pO165
pN170 sR9491
es2298 pW177
0.0
3.2
4.7
5.5
6.3
7.1
7.8
15.7
19.4
26.0
27.5
37.8
48.1
49.7
55.1
57.7
60.2
66.7
72.8
82.3
88.0
sNRF19
sN11542 sC026
CB10026
sR0841 es4424
sN0809 sNR73
sN1933 sN11693
sCMD41 pO85
pO113 es4619
sC595 sN1087
MD41 Ni4D04
sS2329 sC578
sN1958 CB10578
pR54.A08.2 CB10364
sN12113 pR54
sR1345 sC364
pO29
sR02133 pO85.A08.2
sCB39 SF11017
BRAS039 CB10595
sNRC73
pR54.A08.3 sN3804F
pR36 sN13007
sN3733
sR1451 CB10484
sC484
pN96 sN4409F
sN12637 CB10629
sC629 sN1058R
sNRG67 sNRB04
sN4513F
pW146 sNRF84
SF12326 pW146.A08.2
sN12915 sN7161
sC475 CB10475
sN12352 sN10692
sN2193
sc035 CB1035
SF06129
sN11812 sN0202
sN13083
pN34
pN23 pN23.A08.2
pN170 sN2603
sN11864 pW177
es2298
0.0
6.7
11.9
14.5
16.0
17.6
18.5
19.4
20.7
21.7
22.4
23.7
23.9
24.2
24.6
26.4
27.9
34.9
43.3
45.9
48.0
52.0
64.3
67.7
69.8
71.1
78.4
84.3
91.9
102.8
IAA1
M16
Cr2COPL
Na10E02 Na12G05
LAP
Cr1E62
sR9480 IAA1.A08.2
O13
sR6068
Cr2E35
CB10364 Cr1E35
Cc1COPL
sR7167 COP12a
CB10236 CB10013
sS1702
CB10026
sR7178
L08 BRAS039
FAE1
CE8 FAE1.A08.2
T02E35
ACO
IGF3222b
sR3688
A15
IGF1108C
Na12B05
F14
CB10475
G11
Na14G02 Na12A08
E4M7 ScaRa09
CB10193

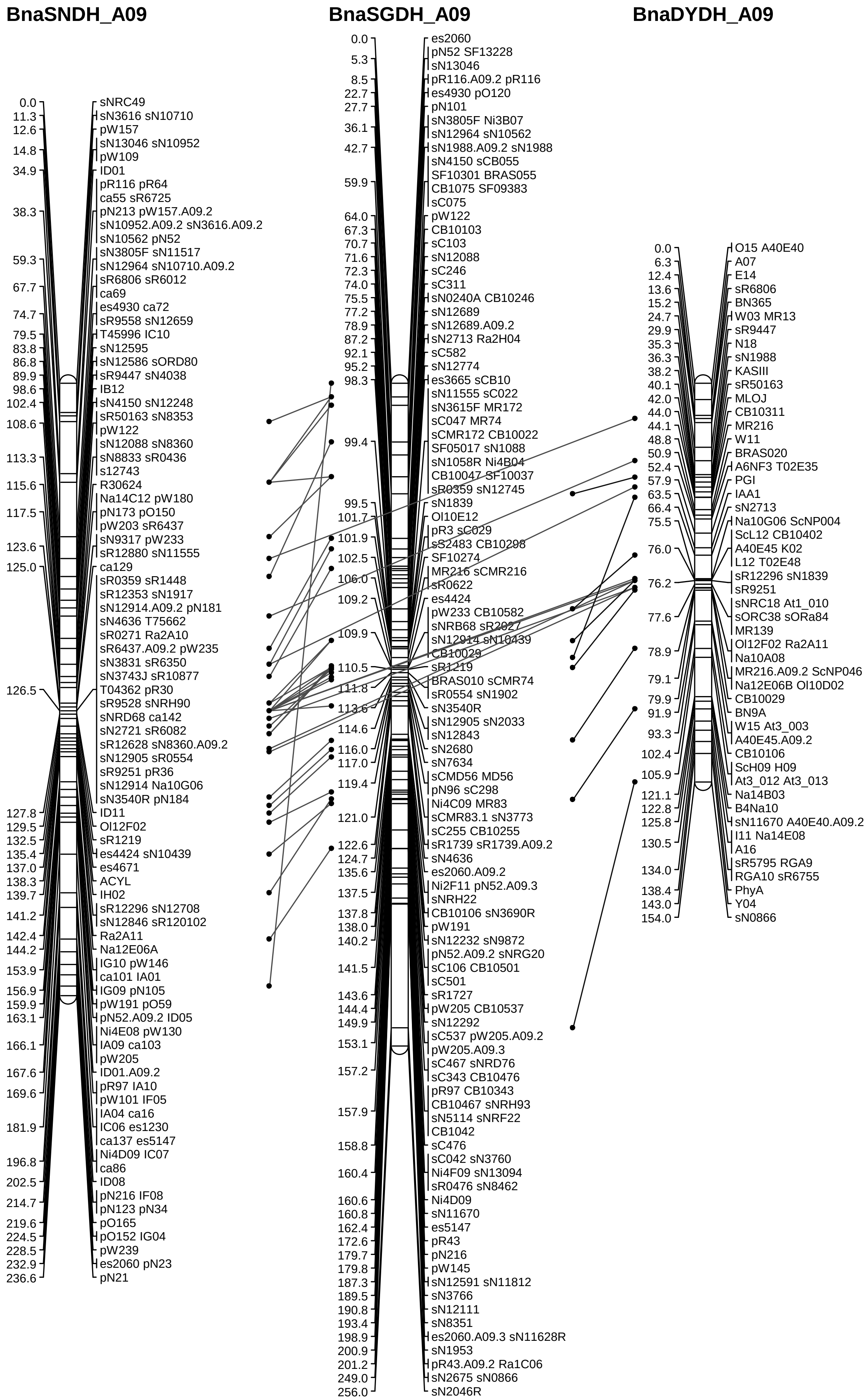
BnaSNDH_A09
0.0
11.3
12.6
14.8
34.9
38.3
59.3
67.7
74.7
79.5
83.8
86.8
89.9
98.6
102.4
108.6
113.3
115.6
117.5
123.6
125.0
126.5
127.8
129.5
132.5
135.4
137.0
138.3
139.7
141.2
142.4
144.2
153.9
156.9
159.9
163.1
166.1
167.6
169.6
181.9
196.8
202.5
214.7
219.6
224.5
228.5
232.9
236.6
sNRC49
sN3616 sN10710
pW157
sN13046 sN10952
pW109
ID01
pR116 pR64
ca55 sR6725
pN213 pW157.A09.2
sN10952.A09.2 sN3616.A09.2
sN10562 pN52
sN3805F sN11517
sN12964 sN10710.A09.2
sR6806 sR6012
ca69
es4930 ca72
sR9558 sN12659
T45996 IC10
sN12595
sN12586 sORD80
sR9447 sN4038
IB12
sN4150 sN12248
sR50163 sN8353
pW122
sN12088 sN8360
sN8833 sR0436
s12743
R30624
Na14C12 pW180
pN173 pO150
pW203 sR6437
sN9317 pW233
sR12880 sN11555
ca129
sR0359 sR1448
sR12353 sN1917
sN12914.A09.2 pN181
sN4636 T75662
sR0271 Ra2A10
sR6437.A09.2 pW235
sN3831 sR6350
sN3743J sR10877
T04362 pR30
sR9528 sNRH90
sNRD68 ca142
sN2721 sR6082
sR12628 sN8360.A09.2
sN12905 sR0554
sR9251 pR36
sN12914 Na10G06
sN3540R pN184
ID11
Ol12F02
sR1219
es4424 sN10439
es4671
ACYL
IH02
sR12296 sN12708
sN12846 sR120102
Ra2A11
Na12E06A
IG10 pW146
ca101 IA01
IG09 pN105
pW191 pO59
pN52.A09.2 ID05
Ni4E08 pW130
IA09 ca103
pW205
ID01.A09.2
pR97 IA10
pW101 IF05
IA04 ca16
IC06 es1230
ca137 es5147
Ni4D09 IC07
ca86
ID08
pN216 IF08
pN123 pN34
pO165
pO152 IG04
pW239
es2060 pN23
pN21
BnaSGDH_A09
0.0
5.3
8.5
22.7
27.7
36.1
42.7
59.9
64.0
67.3
70.7
71.6
72.3
74.0
75.5
77.2
78.9
87.2
92.1
95.2
98.3
99.4
99.5
101.7
101.9
102.5
106.0
109.2
109.9
110.5
111.8
113.6
114.6
116.0
117.0
119.4
121.0
122.6
124.7
135.6
137.5
137.8
138.0
140.2
141.5
143.6
144.4
149.9
153.1
157.2
157.9
158.8
160.4
160.6
160.8
162.4
172.6
179.7
179.8
187.3
189.5
190.8
193.4
198.9
200.9
201.2
249.0
256.0
es2060
pN52 SF13228
sN13046
pR116.A09.2 pR116
es4930 pO120
pN101
sN3805F Ni3B07
sN12964 sN10562
sN1988.A09.2 sN1988
sN4150 sCB055
SF10301 BRAS055
CB1075 SF09383
sC075
pW122
CB10103
sC103
sN12088
sC246
sC311
sN0240A CB10246
sN12689
sN12689.A09.2
sN2713 Ra2H04
sC582
sN12774
es3665 sCB10
sN11555 sC022
sN3615F MR172
sC047 MR74
sCMR172 CB10022
SF05017 sN1088
sN1058R Ni4B04
CB10047 SF10037
sR0359 sN12745
sN1839
Ol10E12
pR3 sC029
sS2483 CB10298
SF10274
MR216 sCMR216
sR0622
es4424
pW233 CB10582
sNRB68 sR2027
sN12914 sN10439
CB10029
sR1219
BRAS010 sCMR74
sR0554 sN1902
sN3540R
sN12905 sN2033
sN12843
sN2680
sN7634
sCMD56 MD56
pN96 sC298
Ni4C09 MR83
sCMR83.1 sN3773
sC255 CB10255
sR1739 sR1739.A09.2
sN4636
es2060.A09.2
Ni2F11 pN52.A09.3
sNRH22
CB10106 sN3690R
pW191
sN12232 sN9872
pN52.A09.2 sNRG20
sC106 CB10501
sC501
sR1727
pW205 CB10537
sN12292
sC537 pW205.A09.2
pW205.A09.3
sC467 sNRD76
sC343 CB10476
pR97 CB10343
CB10467 sNRH93
sN5114 sNRF22
CB1042
sC476
sC042 sN3760
Ni4F09 sN13094
sR0476 sN8462
Ni4D09
sN11670
es5147
pR43
pN216
pW145
sN12591 sN11812
sN3766
sN12111
sN8351
es2060.A09.3 sN11628R
sN1953
pR43.A09.2 Ra1C06
sN2675 sN0866
sN2046R
BnaDYDH_A09
0.0
6.3
12.4
13.6
15.2
24.7
29.9
35.3
36.3
38.2
40.1
42.0
44.0
44.1
48.8
50.9
52.4
57.9
63.5
66.4
75.5
76.0
76.2
77.6
78.9
79.1
79.9
91.9
93.3
102.4
105.9
121.1
122.8
125.8
130.5
134.0
138.4
143.0
154.0
O15 A40E40
A07
E14
sR6806
BN365
W03 MR13
sR9447
N18
sN1988
KASIII
sR50163
MLOJ
CB10311
MR216
W11
BRAS020
A6NF3 T02E35
PGI
IAA1
sN2713
Na10G06 ScNP004
ScL12 CB10402
A40E45 K02
L12 T02E48
sR12296 sN1839
sR9251
sNRC18 At1_010
sORC38 sORa84
MR139
Ol12F02 Ra2A11
Na10A08
MR216.A09.2 ScNP046
Na12E06B Ol10D02
CB10029
BN9A
W15 At3_003
A40E45.A09.2
CB10106
ScH09 H09
At3_012 At3_013
Na14B03
B4Na10
sN11670 A40E40.A09.2
I11 Na14E08
A16
sR5795 RGA9
RGA10 sR6755
PhyA
Y04
sN0866

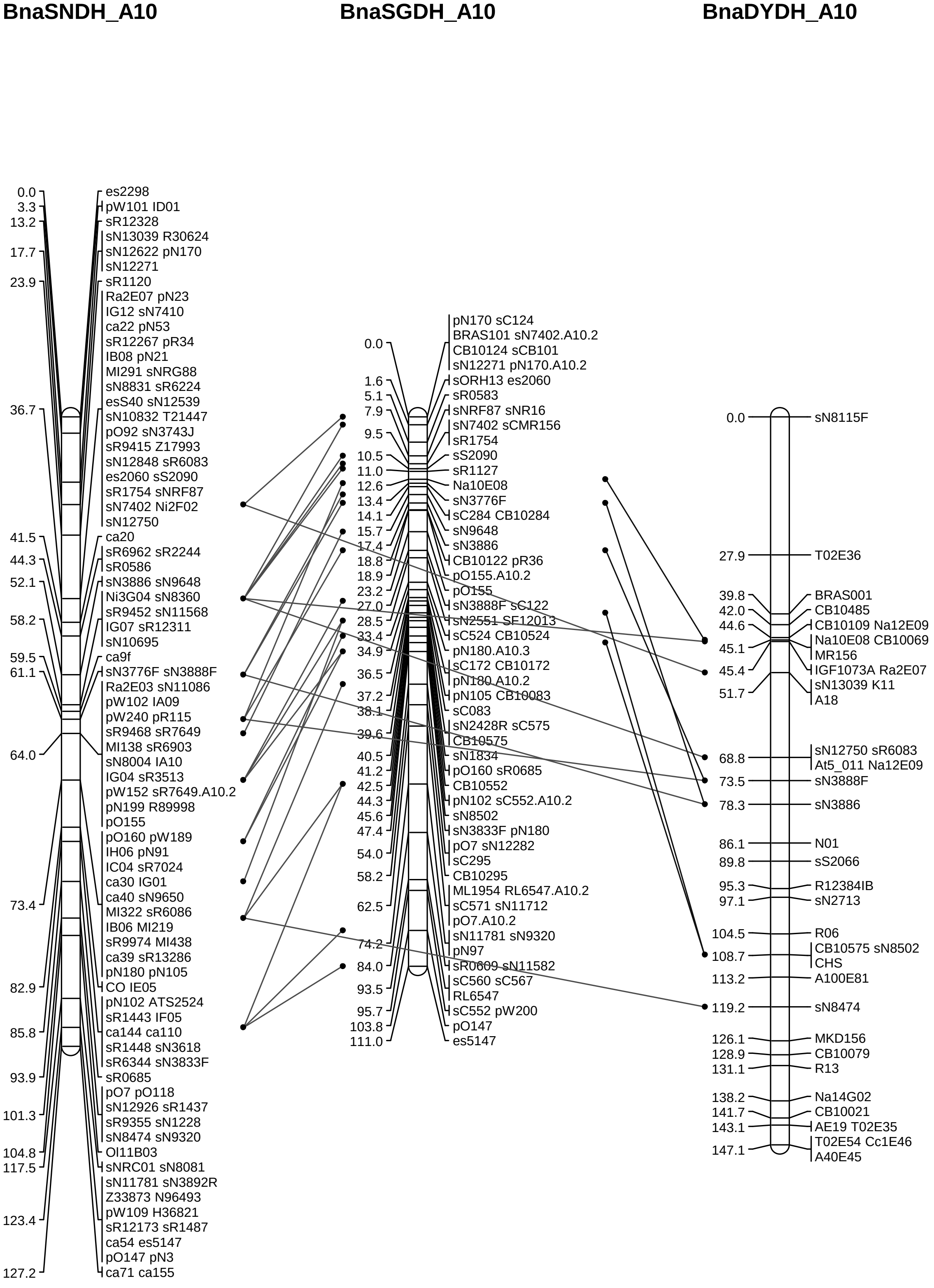
BnaSNDH_A10
BnaSGDH_A10
BnaDYDH_A10
0.0 es2298
3.3 pW101 ID01
13.2 sR12328
sN13039 R30624
17.7 sN12622 pN170
sN12271
23.9 sR1120
Ra2E07 pN23
IG12 sN7410
ca22 pN53
sR12267 pR34
IB08 pN21
MI291 sNRG88
sN8831 sR6224
esS40 sN12539
36.7 sN10832 T21447
pO92 sN3743J
sR9415 Z17993
sN12848 sR6083
es2060 sS2090
sR1754 sNRF87
sN7402 Ni2F02
sN12750
41.5 ca20
44.3 sR6962 sR2244
sR0586
52.1 sN3886 sN9648
Ni3G04 sN8360
sR9452 sN11568
58.2 IG07 sR12311
sN10695
59.5 ca9f
61.1 sN3776F sN3888F
Ra2E03 sN11086
pW102 IA09
pW240 pR115
sR9468 sR7649
64.0 MI138 sR6903
sN8004 IA10
IG04 sR3513
pW152 sR7649.A10.2
pN199 R89998
pO155
pO160 pW189
IH06 pN91
IC04 sR7024
ca30 IG01
ca40 sN9650
73.4 MI322 sR6086
IB06 MI219
sR9974 MI438
ca39 sR13286
pN180 pN105
82.9 CO IE05
pN102 ATS2524
sR1443 IF05
85.8 ca144 ca110
sR1448 sN3618
sR6344 sN3833F
93.9 sR0685
pO7 pO118
101.3 sN12926 sR1437
sR9355 sN1228
sN8474 sN9320
104.8 OI11B03
117.5 sNRC01 sN8081
sN11781 sN3892R
Z33873 N96493
pW109 H36821
123.4 sR12173 sR1487
ca54 es5147
pO147 pN3
127.2 ca71 ca155
0.0 pN170 sC124
BRAS101 sN7402.A10.2
CB10124 sCB101
sN12271 pN170.A10.2
1.6 sORH13 es2060
5.1 sR0583
7.9 sNRF87 sNR16
9.5 sN7402 sCMR156
sR1754
10.5 sS2090
11.0 sR1127
12.6 Na10E08
13.4 sN3776F
14.1 sC284 CB10284
15.7 sN9648
17.4 sN3886
18.8 CB10122 pR36
18.9 pO155.A10.2
23.2 pO155
27.0 sN3888F sC122
28.5 sN2551 SF12013
33.4 sC524 CB10524
34.9 pN180.A10.3
36.5 sC172 CB10172
pN180 A10.2
37.2 pN105 CB10083
38.1 sC083
39.6 sN2428R sC575
CB10575
40.5 sN1834
41.2 pO160 sR0685
42.5 CB10552
44.3 pN102 sC552.A10.2
45.6 sN8502
47.4 sN3833F pN180
54.0 pO7 sN12282
58.2 sC295
CB10295
62.5 ML1954 RL6547.A10.2
sC571 sN11712
pO7.A10.2
74.2 sN11781 sN9320
pN97
84.0 sR0609 sN11582
93.5 sC560 sC567
RL6547
95.7 sC552 pW200
103.8 pO147
111.0 es5147
0.0 sN8115F
27.9 T02E36
39.8 BRAS001
42.0 CB10485
44.6 CB10109 Na12E09
45.1 Na10E08 CB10069
MR156
45.4 IGF1073A Ra2E07
51.7 sN13039 K11
A18
68.8 sN12750 sR6083
At5_011 Na12E09
73.5 sN3888F
78.3 sN3886
86.1 N01
89.8 sS2066
95.3 R12384IB
97.1 sN2713
104.5 R06
108.7 CB10575 sN8502
CHS
113.2 A100E81
119.2 sN8474
126.1 MKD156
128.9 CB10079
131.1 R13
138.2 Na14G02
141.7 CB10021
143.1 AE19 T02E35
147.1 T02E54 Cc1E46
A40E45

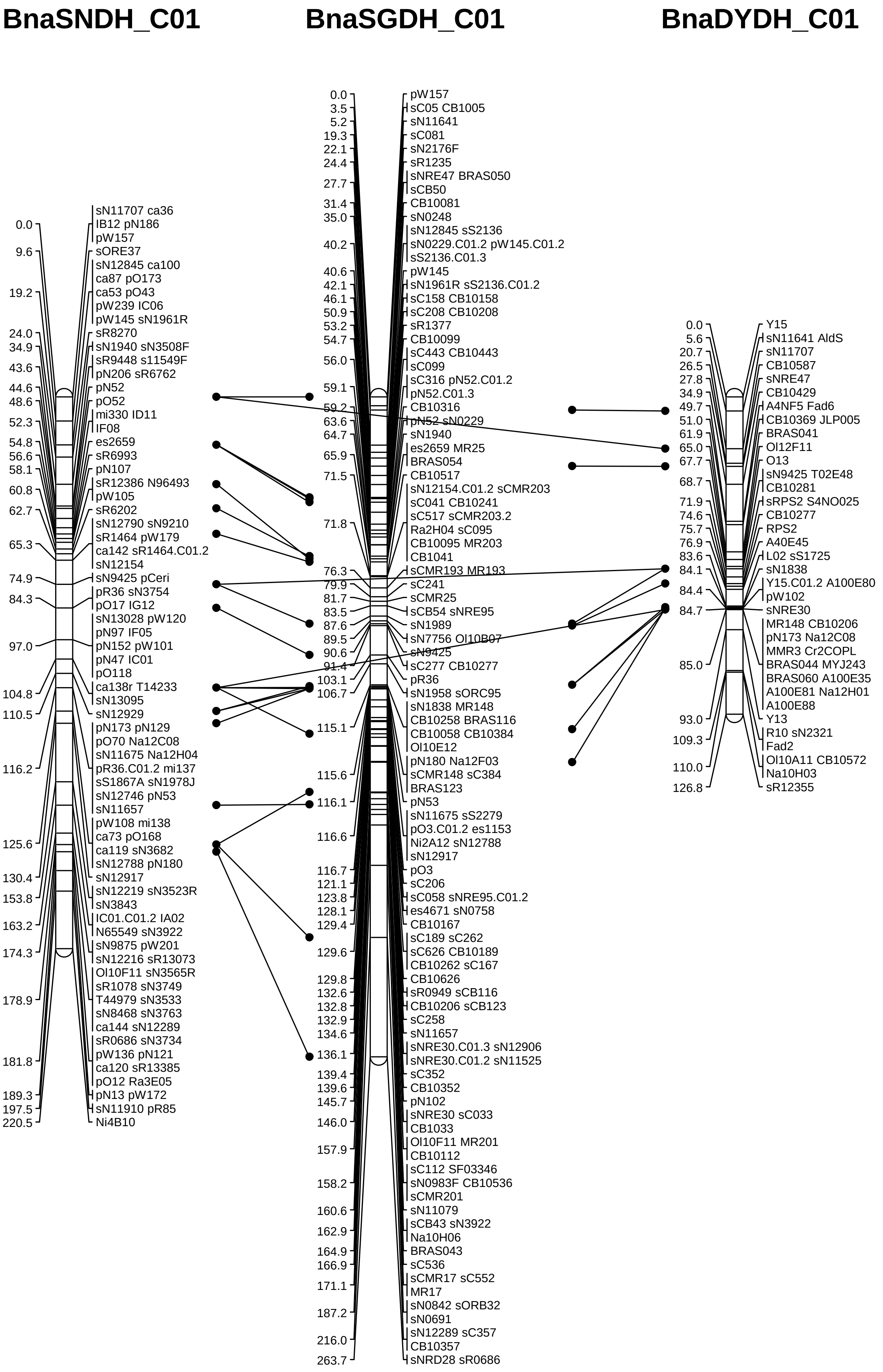
BnaSNDH_C01
BnaSGDH_C01
BnaDYDH_C01
0.0
9.6
19.2
24.0
34.9
43.6
44.6
48.6
52.3
54.8
56.6
58.1
60.8
62.7
65.3
74.9
84.3
97.0
104.8
110.5
116.2
125.6
130.4
153.8
163.2
174.3
178.9
181.8
189.3
197.5
220.5
sN11707 ca36
IB12 pN186
pW157
sORE37
sN12845 ca100
ca87 pO173
ca53 pO43
pW239 IC06
pW145 sN1961R
sR8270
sN1940 sN3508F
sR9448 s11549F
pN206 sR6762
pN52
pO52
mi330 ID11
IF08
es2659
sR6993
pN107
sR12386 N96493
pW105
sR6202
sN12790 sN9210
sR1464 pW179
ca142 sR1464.C01.2
sN12154
sN9425 pCeri
pR36 sN3754
pO17 IG12
sN13028 pW120
pN97 IF05
pN152 pW101
pN47 IC01
pO118
ca138r T14233
sN13095
sN12929
pN173 pN129
pO70 Na12C08
sN11675 Na12H04
pR36.C01.2 mi137
sS1867A sN1978J
sN12746 pN53
sN11657
pW108 mi138
ca73 pO168
ca119 sN3682
sN12788 pN180
sN12917
sN12219 sN3523R
sN3843
IC01.C01.2 IA02
N65549 sN3922
sN9875 pW201
sN12216 sR13073
Ol10F11 sN3565R
sR1078 sN3749
T44979 sN3533
sN8468 sN3763
ca144 sN12289
sR0686 sN3734
pW136 pN121
ca120 sR13385
pO12 Ra3E05
pN13 pW172
sN11910 pR85
Ni4B10
0.0
3.5
5.2
19.3
22.1
24.4
27.7
31.4
35.0
40.2
40.6
42.1
46.1
50.9
53.2
54.7
56.0
59.1
59.2
63.6
64.7
65.9
71.5
71.8
76.3
79.9
81.7
83.5
87.6
89.5
90.6
91.4
103.1
106.7
115.1
115.6
116.1
116.6
116.7
121.1
123.8
128.1
129.4
129.6
129.8
132.6
132.8
132.9
134.6
136.1
139.4
139.6
145.7
146.0
157.9
158.2
160.6
162.9
164.9
166.9
171.1
187.2
216.0
263.7
pW157
sC05 CB1005
sN11641
sC081
sN2176F
sR1235
sNRE47 BRAS050
sCB50
CB10081
sN0248
sN12845 sS2136
sN0229.C01.2 pW145.C01.2
sS2136.C01.3
pW145
sN1961R sS2136.C01.2
sC158 CB10158
sC208 CB10208
sR1377
CB10099
sC443 CB10443
sC099
sC316 pN52.C01.2
pN52.C01.3
CB10316
pN52 sN0229
sN1940
es2659 MR25
BRAS054
CB10517
sN12154.C01.2 sCMR203
sC041 CB10241
sC517 sCMR203.2
Ra2H04 sC095
CB10095 MR203
CB1041
sCMR193 MR193
sC241
sCMR25
sCB54 sNRE95
sN1989
sN7756 Ol10B07
sN9425
sC277 CB10277
pR36
sN1958 sORC95
sN1838 MR148
CB10258 BRAS116
CB10058 CB10384
Ol10E12
pN180 Na12F03
sCMR148 sC384
BRAS123
pN53
sN11675 sS2279
pO3.C01.2 es1153
Ni2A12 sN12788
sN12917
pO3
sC206
sC058 sNRE95.C01.2
es4671 sN0758
CB10167
sC189 sC262
sC626 CB10189
CB10262 sC167
CB10626
sR0949 sCB116
CB10206 sCB123
sC258
sN11657
sNRE30.C01.3 sN12906
sNRE30.C01.2 sN11525
sC352
CB10352
pN102
sNRE30 sC033
CB1033
Ol10F11 MR201
CB10112
sC112 SF03346
sN0983F CB10536
sCMR201
sN11079
sCB43 sN3922
Na10H06
BRAS043
sC536
sCMR17 sC552
MR17
sN0842 sORB32
sN0691
sN12289 sC357
CB10357
sNRD28 sR0686
0.0
5.6
20.7
26.5
27.8
34.9
49.7
51.0
61.9
65.0
67.7
68.7
71.9
74.6
75.7
76.9
83.6
84.1
84.4
84.7
85.0
93.0
109.3
110.0
126.8
Y15
sN11641 AldS
sN11707
CB10587
sNRE47
CB10429
A4NF5 Fad6
CB10369 JLP005
BRAS041
Ol12F11
O13
sN9425 T02E48
CB10281
sRPS2 S4NO025
CB10277
RPS2
A40E45
L02 sS1725
sN1838
Y15.C01.2 A100E80
pW102
sNRE30
MR148 CB10206
pN173 Na12C08
MMR3 Cr2COPL
BRAS044 MYJ243
BRAS060 A100E35
A100E81 Na12H01
A100E88
Y13
R10 sN2321
Fad2
Ol10A11 CB10572
Na10H03
sR12355

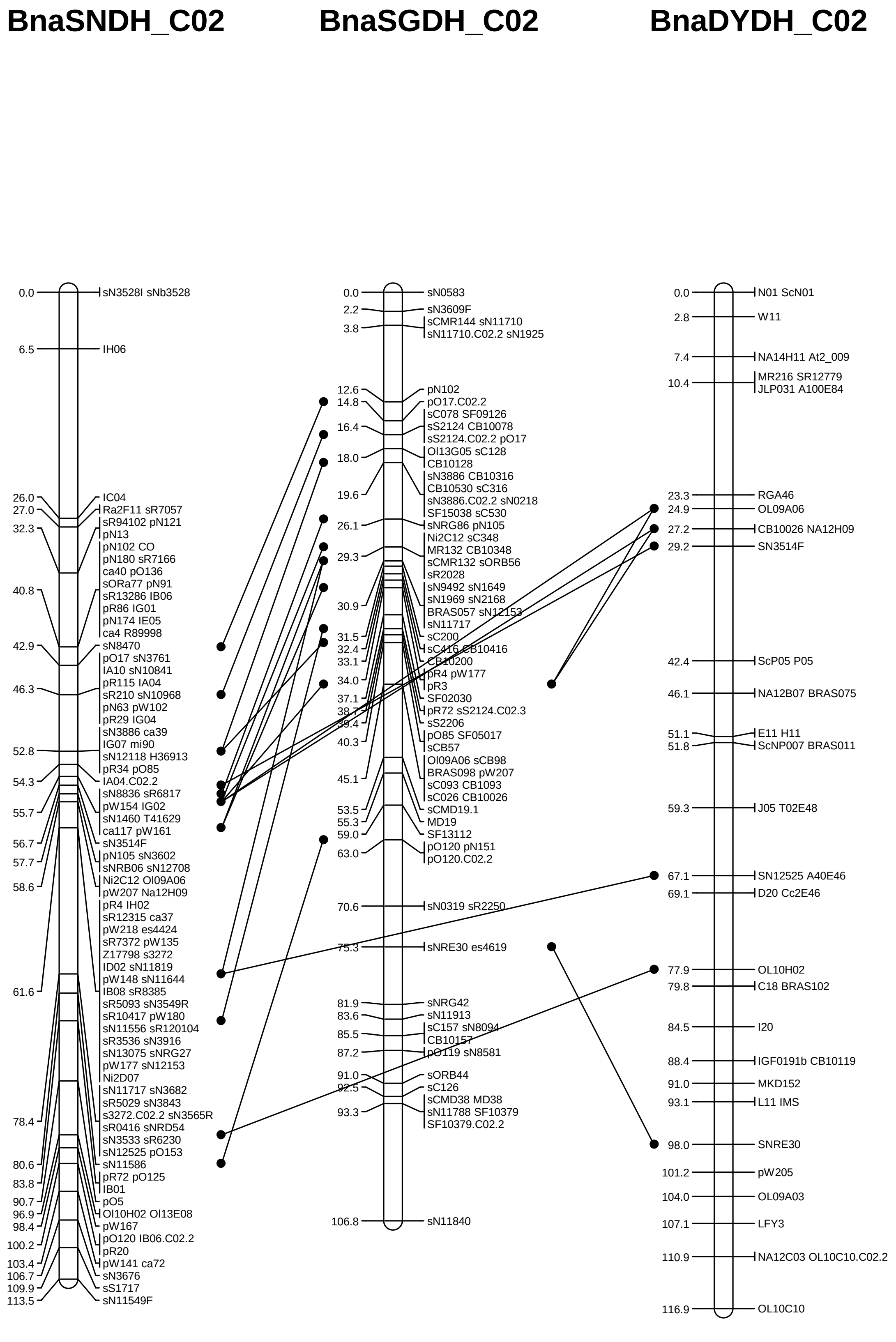
BnaSNDH_C02
BnaSGDH_C02
BnaDYDH_C02
0.0 sN3528I sNb3528
6.5 IH06
26.0 IC04
27.0 Ra2F11 sR7057
32.3 sR94102 pN121
pN13
pN102 CO
pN180 sR7166
ca40 pO136
sORa77 pN91
40.8 sR13286 IB06
pR86 IG01
pN174 IE05
ca4 R89998
42.9 sN8470
pO17 sN3761
IA10 sN10841
pR115 IA04
46.3 sR210 sN10968
pN63 pW102
pR29 IG04
sN3886 ca39
IG07 mi90
52.8 sN12118 H36913
pR34 pO85
54.3 IA04.C02.2
sN8836 sR6817
pW154 IG02
55.7 sN1460 T41629
ca117 pW161
56.7 sN3514F
57.7 pN105 sN3602
sNRB06 sN12708
58.6 Ni2C12 OI09A06
pW207 Na12H09
pR4 IH02
sR12315 ca37
pW218 es4424
sR7372 pW135
Z17798 s3272
ID02 sN11819
pW148 sN11644
61.6 IB08 sR8385
sR5093 sN3549R
sR10417 pW180
sN11556 sR120104
sR3536 sN3916
sN13075 sNRG27
pW177 sN12153
Ni2D07
sN11717 sN3682
sR5029 sN3843
78.4 s3272.C02.2 sN3565R
sR0416 sNRD54
sN3533 sR6230
sN12525 pO153
80.6 sN11586
83.8 pR72 pO125
IB01
90.7 pO5
96.9 OI10H02 OI13E08
98.4 pW167
100.2 pO120 IB06.C02.2
pR20
103.4 pW141 ca72
106.7 sN3676
109.9 sS1717
113.5 sN11549F
0.0 sN0583
2.2 sN3609F
3.8 sCMR144 sN11710
sN11710.C02.2 sN1925
12.6 pN102
14.8 pO17.C02.2
sC078 SF09126
16.4 sS2124 CB10078
sS2124.C02.2 pO17
18.0 OI13G05 sC128
CB10128
sN3886 CB10316
19.6 CB10530 sC316
sN3886.C02.2 sN0218
SF15038 sC530
26.1 sNRG86 pN105
Ni2C12 sC348
29.3 MR132 CB10348
sCMR132 sORB56
sR2028
30.9 sN9492 sN1649
sN1969 sN2168
BRAS057 sN12153
sN11717
31.5 sC200
32.4 sC416 CB10416
33.1 CB10200
34.0 pR4 pW177
pR3
37.1 SF02030
38.7 pR72 sS2124.C02.3
39.4 sS2206
40.3 pO85 SF05017
sCB57
OI09A06 sCB98
45.1 BRAS098 pW207
sC093 CB1093
sC026 CB10026
53.5 sCMD19.1
55.3 MD19
59.0 SF13112
63.0 pO120 pN151
pO120.C02.2
70.6 sN0319 sR2250
75.3 sNRE30 es4619
81.9 sNRG42
83.6 sN11913
85.5 sC157 sN8094
CB10157
87.2 pO119 sN8581
91.0 sORB44
92.5 sC126
93.3 sCMD38 MD38
sN11788 SF10379
SF10379.C02.2
106.8 sN11840
0.0 N01 ScN01
2.8 W11
7.4 NA14H11 At2_009
10.4 MR216 SR12779
JLP031 A100E84
23.3 RGA46
24.9 OL09A06
27.2 CB10026 NA12H09
29.2 SN3514F
42.4 ScP05 P05
46.1 NA12B07 BRAS075
51.1 E11 H11
51.8 ScNP007 BRAS011
59.3 J05 T02E48
67.1 SN12525 A40E46
69.1 D20 Cc2E46
77.9 OL10H02
79.8 C18 BRAS102
84.5 I20
88.4 IGF0191b CB10119
91.0 MKD152
93.1 L11 IMS
98.0 SNRE30
101.2 pW205
104.0 OL09A03
107.1 LFY3
110.9 NA12C03 OL10C10.C02.2
116.9 OL10C10

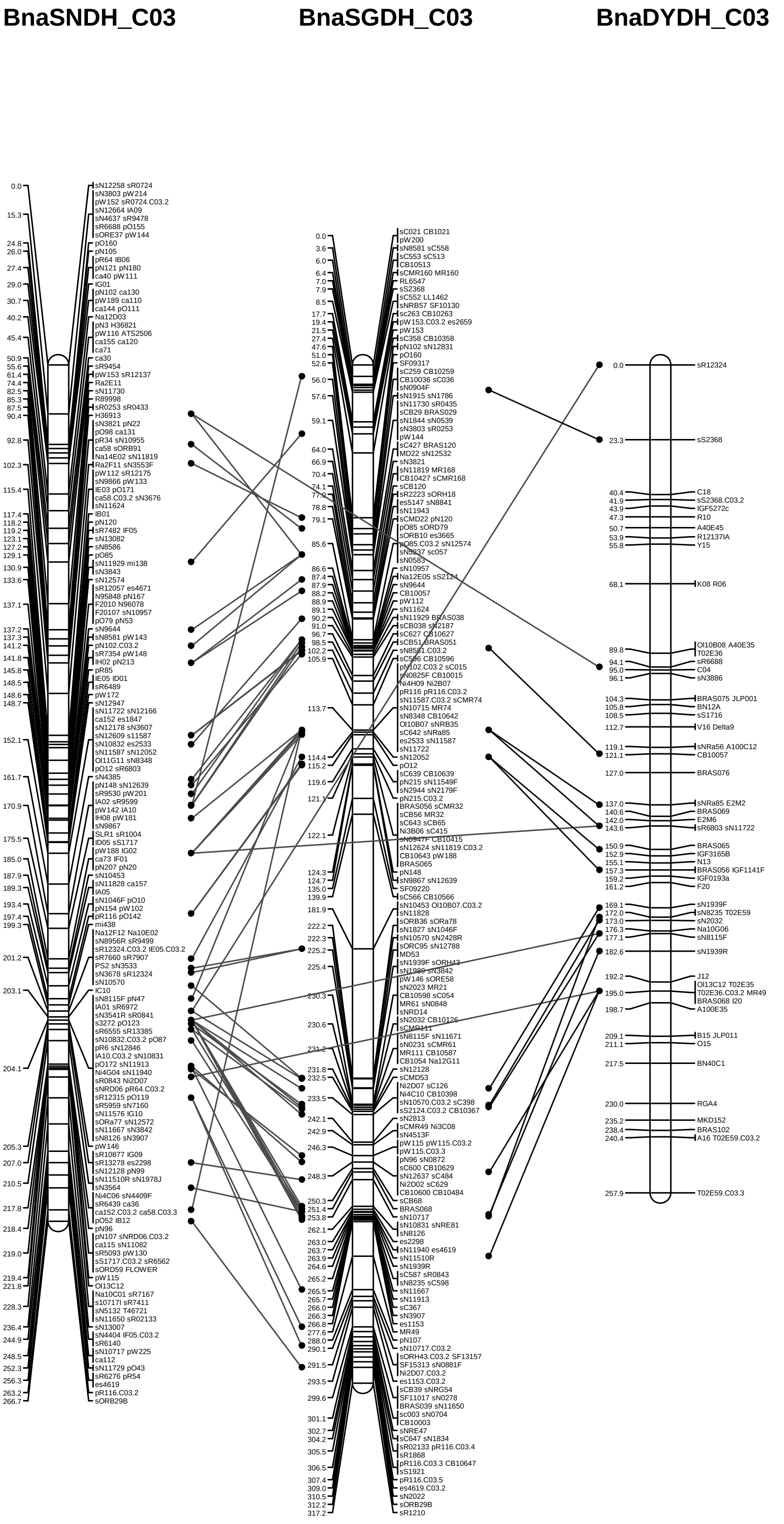
BnaSNDH_C03
BnaSGDH_C03
BnaDYDH_C03

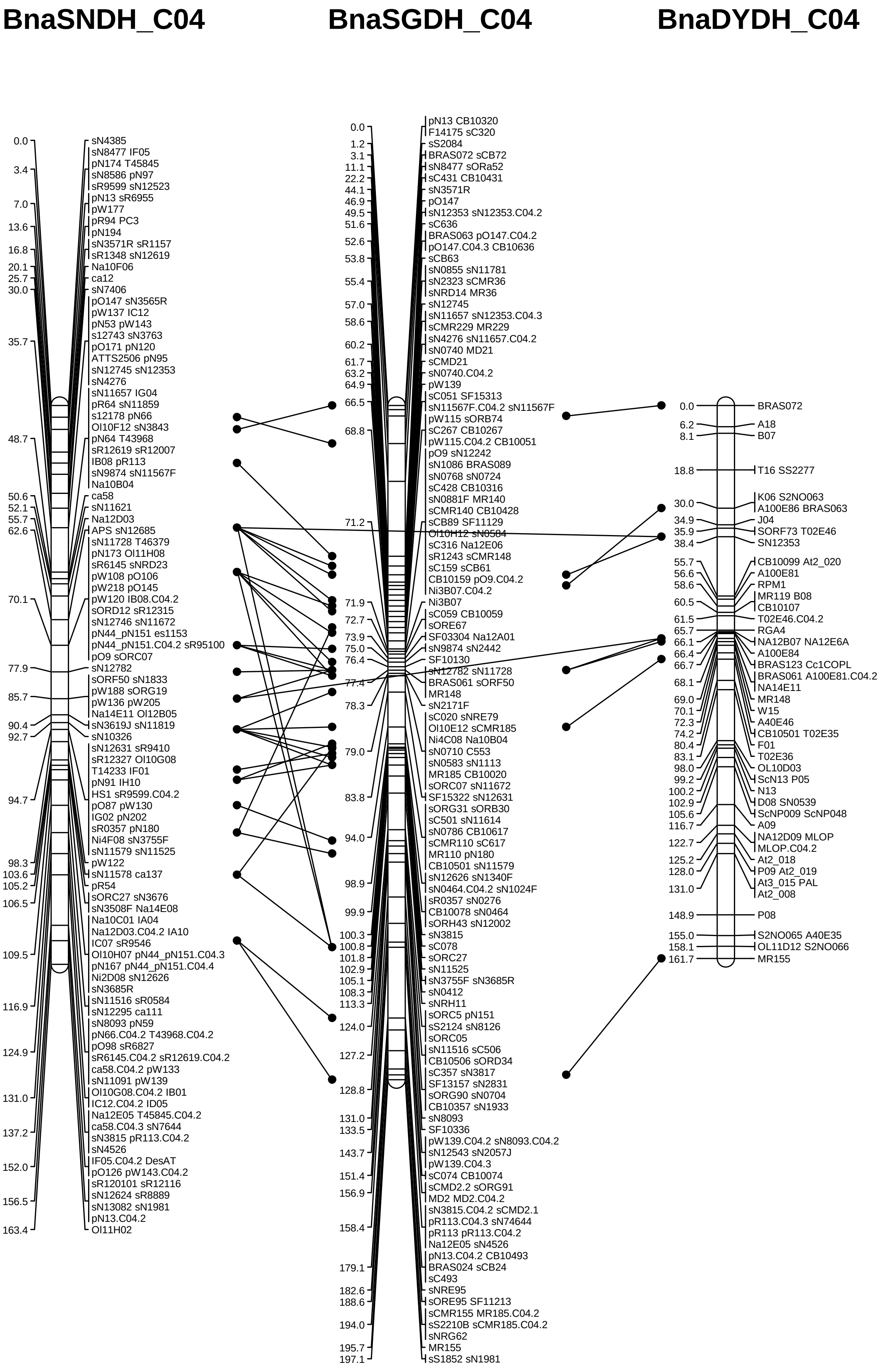
BnaSNDH_C04
BnaSGDH_C04
BnaDYDH_C04
0.0 3.4 7.0 13.6 16.8 20.1 25.7 30.0 35.7 48.7 50.6 52.1 55.7 62.6 70.1 77.9 85.7 90.4 92.7 94.7 98.3 103.6 105.2 106.5 109.5 116.9 124.9 131.0 137.2 152.0 156.5 163.4
sN4385
sN8477 IF05
pN174 T45845
sN8586 pN97
sR9599 sN12523
pN13 sR6955
pW177
pR94 PC3
pN194
sN3571R sR1157
sR1348 sN12619
Na10F06
ca12
sN7406
pO147 sN3565R
pW137 IC12
pN53 pW143
s12743 sN3763
pO171 pN120
ATTS2506 pN95
sN12745 sN12353
sN4276
sN11657 IG04
pR64 sN11859
s12178 pN66
Ol10F12 sN3843
pN64 T43968
sR12619 sR12007
IB08 pR113
sN9874 sN11567F
Na10B04
ca58
sN11621
Na12D03
APS sN12685
sN11728 T46379
pN173 Ol11H08
sR6145 sNRD23
pW108 pO106
pW218 pO145
pW120 IB08.C04.2
sORD12 sR12315
sN12746 sN11672
pN44_pN151 es1153
pN44_pN151.C04.2 sR95100
pO9 sORC07
sN12782
sORF50 sN1833
pW188 sORG19
pW136 pW205
Na14E11 Ol12B05
sN3619J sN11819
sN10326
sN12631 sR9410
sR12327 Ol10G08
T14233 IF01
pN91 IH10
HS1 sR9599.C04.2
pO87 pW130
IG02 pN202
sR0357 pN180
Ni4F08 sN3755F
sN11579 sN11525
pW122
sN11578 ca137
pR54
sORC27 sN3676
sN3508F Na14E08
Na10C01 IA04
Na12D03.C04.2 IA10
IC07 sR9546
Ol10H07 pN44_pN151.C04.3
pN167 pN44_pN151.C04.4
Ni2D08 sN12626
sN3685R
sN11516 sR0584
sN12295 ca111
sN8093 pN59
pN66.C04.2 T43968.C04.2
pO98 sR6827
sR6145.C04.2 sR12619.C04.2
ca58.C04.2 pW133
sN11091 pW139
Ol10G08.C04.2 IB01
IC12.C04.2 ID05
Na12E05 T45845.C04.2
ca58.C04.3 sN7644
sN3815 pR113.C04.2
sN4526
IF05.C04.2 DesAT
pO126 pW143.C04.2
sR120101 sR12116
sN12624 sR8889
sN13082 sN1981
pN13.C04.2
Ol11H02
0.0 1.2 3.1 11.1 22.2 44.1 46.9 49.5 51.6 52.6 53.8 55.4 57.0 58.6 60.2 61.7 63.2 64.9 66.5 68.8 71.2 71.9 72.7 73.9 75.0 76.4 77.4 78.3 79.0 83.8 94.0 98.9 99.9 100.3 100.8 101.8 102.9 105.1 108.3 113.3 124.0 127.2 128.8 131.0 133.5 143.7 151.4 156.9 158.4 179.1 182.6 188.6 194.0 195.7 197.1
pN13 CB10320
F14175 sC320
sS2084
BRAS072 sCB72
sN8477 sORa52
sC431 CB10431
sN3571R
pO147
sN12353 sN12353.C04.2
sC636
BRAS063 pO147.C04.2
pO147.C04.3 CB10636
sCB63
sN0855 sN11781
sN2323 sCMR36
sNRD14 MR36
sN12745
sN11657 sN12353.C04.3
sCMR229 MR229
sN4276 sN11657.C04.2
sN0740 MD21
sCMD21
sN0740.C04.2
pW139
sC051 SF15313
sN11567F.C04.2 sN11567F
pW115 sORB74
sC267 CB10267
pW115.C04.2 CB10051
pO9 sN12242
sN1086 BRAS089
sN0768 sN0724
sC428 CB10316
sN0881F MR140
sCMR140 CB10428
sCB89 SF11129
Ol10H12 sN0584
sC316 Na12E06
sR1243 sCMR148
sC159 sCB61
CB10159 pO9.C04.2
Ni3B07.C04.2
Ni3B07
sC059 CB10059
sORE67
SF03304 Na12A01
sN9874 sN2442
SF10130
sN12782 sN11728
BRAS061 sORF50
MR148
sN2171F
sC020 sNRE79
Ol10E12 sCMR185
Ni4C08 Na10B04
sN0710 C553
sN0583 sN1113
MR185 CB10020
sORC07 sN11672
SF15322 sN12631
sORG31 sORB30
sC501 sN11614
sN0786 CB10617
sCMR110 sC617
MR110 pN180
CB10501 sN11579
sN12626 sN1340F
sN0464.C04.2 sN1024F
sR0357 sN0276
CB10078 sN0464
sORH43 sN12002
sN3815
sC078
sORC27
sN11525
sN3755F sN3685R
sN0412
sNRH11
sORC5 pN151
sS2124 sN8126
sORC05
sN11516 sC506
CB10506 sORD34
sC357 sN3817
SF13157 sN2831
sORG90 sN0704
CB10357 sN1933
sN8093
SF10336
pW139.C04.2 sN8093.C04.2
sN12543 sN2057J
pW139.C04.3
sC074 CB10074
sCMD2.2 sORG91
MD2 MD2.C04.2
sN3815.C04.2 sCMD2.1
pR113.C04.3 sN74644
pR113 pR113.C04.2
Na12E05 sN4526
pN13.C04.2 CB10493
BRAS024 sCB24
sC493
sNRE95
sORE95 SF11213
sCMR155 MR185.C04.2
sS2210B sCMR185.C04.2
sNRG62
MR155
sS1852 sN1981
0.0 6.2 8.1 18.8 30.0 34.9 35.9 38.4 55.7 56.6 58.6 60.5 61.5 65.7 66.1 66.4 66.7 68.1 69.0 70.1 72.3 74.2 80.4 83.1 98.0 99.2 100.2 102.9 105.6 116.7 122.7 125.2 128.0 131.0 148.9 155.0 158.1 161.7
BRAS072
A18
B07
T16 SS2277
K06 S2NO063
A100E86 BRAS063
J04
SORF73 T02E46
SN12353
CB10099 At2_020
A100E81
RPM1
MR119 B08
CB10107
T02E46.C04.2
RGA4
NA12B07 NA12E6A
A100E84
BRAS123 Cc1COPL
BRAS061 A100E81.C04.2
NA14E11
MR148
W15
A40E46
CB10501 T02E35
F01
T02E36
OL10D03
ScN13 P05
N13
D08 SN0539
ScNP009 ScNP048
A09
NA12D09 MLOP
MLOP.C04.2
At2_018
P09 At2_019
At3_015 PAL
At2_008
P08
S2NO065 A40E35
OL11D12 S2NO066
MR155

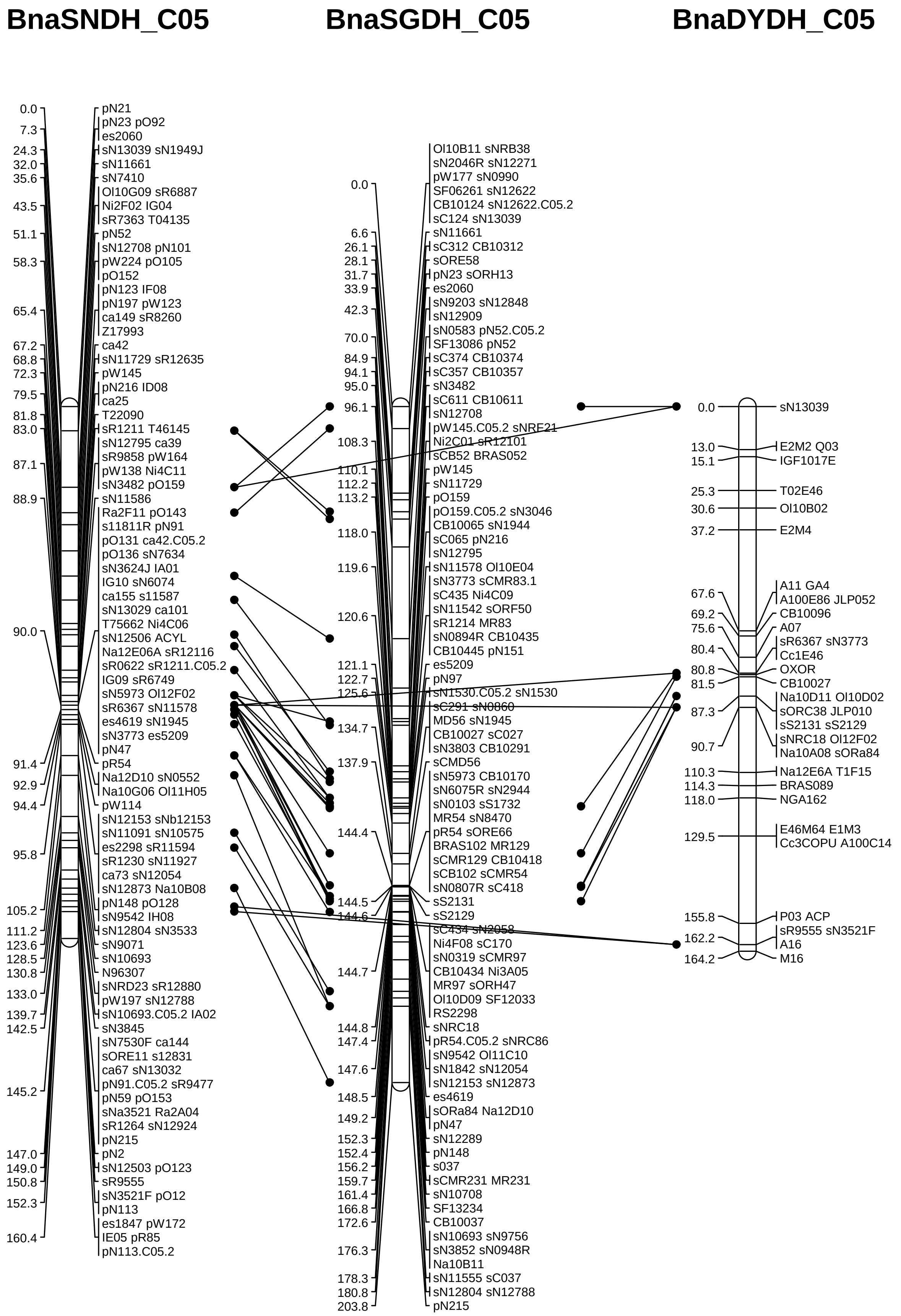
BnaSNDH_C05
BnaSGDH_C05
BnaDYDH_C05
0.0
7.3
24.3
32.0
35.6
43.5
51.1
58.3
65.4
67.2
68.8
72.3
79.5
81.8
83.0
87.1
88.9
90.0
91.4
92.9
94.4
95.8
105.2
111.2
123.6
128.5
130.8
133.0
139.7
142.5
145.2
147.0
149.0
150.8
152.3
160.4
pN21
pN23 pO92
es2060
sN13039 sN1949J
sN11661
sN7410
Ol10G09 sR6887
Ni2F02 IG04
sR7363 T04135
pN52
sN12708 pN101
pW224 pO105
pO152
pN123 IF08
pN197 pW123
ca149 sR8260
Z17993
ca42
sN11729 sR12635
pW145
pN216 ID08
ca25
T22090
sR1211 T46145
sN12795 ca39
sR9858 pW164
pW138 Ni4C11
sN3482 pO159
sN11586
Ra2F11 pO143
s11811R pN91
pO131 ca42.C05.2
pO136 sN7634
sN3624J IA01
IG10 sN6074
ca155 s11587
sN13029 ca101
T75662 Ni4C06
sN12506 ACYL
Na12E06A sR12116
sR0622 sR1211.C05.2
IG09 sR6749
sN5973 Ol12F02
sR6367 sN11578
es4619 sN1945
sN3773 es5209
pN47
pR54
Na12D10 sN0552
Na10G06 Ol11H05
pW114
sN12153 sNb12153
sN11091 sN10575
es2298 sR11594
sR1230 sN11927
ca73 sN12054
sN12873 Na10B08
pN148 pO128
sN9542 IH08
sN12804 sN3533
sN9071
sN10693
N96307
sNRD23 sR12880
pW197 sN12788
sN10693.C05.2 IA02
sN3845
sN7530F ca144
sORE11 s12831
ca67 sN13032
pN91.C05.2 sR9477
pN59 pO153
sNa3521 Ra2A04
sR1264 sN12924
pN215
pN2
sN12503 pO123
sR9555
sN3521F pO12
pN113
es1847 pW172
IE05 pR85
pN113.C05.2
0.0
6.6
26.1
28.1
31.7
33.9
42.3
70.0
84.9
94.1
95.0
96.1
108.3
110.1
112.2
113.2
118.0
119.6
120.6
121.1
122.7
125.6
134.7
137.9
144.4
144.5
144.6
144.7
144.8
147.4
147.6
148.5
149.2
152.3
152.4
156.2
159.7
161.4
166.8
172.6
176.3
178.3
180.8
203.8
Ol10B11 sNRB38
sN2046R sN12271
pW177 sN0990
SF06261 sN12622
CB10124 sN12622.C05.2
sC124 sN13039
sN11661
sC312 CB10312
sORE58
pN23 sORH13
es2060
sN9203 sN12848
sN12909
sN0583 pN52.C05.2
SF13086 pN52
sC374 CB10374
sC357 CB10357
sN3482
sC611 CB10611
sN12708
pW145.C05.2 sNRF21
Ni2C01 sR12101
sCB52 BRAS052
pW145
sN11729
pO159
pO159.C05.2 sN3046
CB10065 sN1944
sC065 pN216
sN12795
sN11578 Ol10E04
sN3773 sCMR83.1
sC435 Ni4C09
sN11542 sORF50
sR1214 MR83
sN0894R CB10435
CB10445 pN151
es5209
pN97
sN1530.C05.2 sN1530
sC291 sN0860
MD56 sN1945
CB10027 sC027
sN3803 CB10291
sCMD56
sN5973 CB10170
sN6075R sN2944
sN0103 sS1732
MR54 sN8470
pR54 sORE66
BRAS102 MR129
sCMR129 CB10418
sCB102 sCMR54
sN0807R sC418
sS2131
sS2129
sC434 sN2058
Ni4F08 sC170
sN0319 sCMR97
CB10434 Ni3A05
MR97 sORH47
Ol10D09 SF12033
RS2298
sNRC18
pR54.C05.2 sNRC86
sN9542 Ol11C10
sN1842 sN12054
sN12153 sN12873
es4619
sORa84 Na12D10
pN47
sN12289
pN148
s037
sCMR231 MR231
sN10708
SF13234
CB10037
sN10693 sN9756
sN3852 sN0948R
Na10B11
sN11555 sC037
sN12804 sN12788
pN215
0.0
13.0
15.1
25.3
30.6
37.2
67.6
69.2
75.6
80.4
80.8
81.5
87.3
90.7
110.3
114.3
118.0
129.5
155.8
162.2
164.2
sN13039
E2M2 Q03
IGF1017E
T02E46
Ol10B02
E2M4
A11 GA4
A100E86 JLP052
CB10096
A07
sR6367 sN3773
Cc1E46
OXOR
CB10027
Na10D11 Ol10D02
sORC38 JLP010
sS2131 sS2129
sNRC18 Ol12F02
Na10A08 sORa84
Na12E6A T1F15
BRAS089
NGA162
E46M64 E1M3
Cc3COPU A100C14
P03 ACP
sR9555 sN3521F
A16
M16

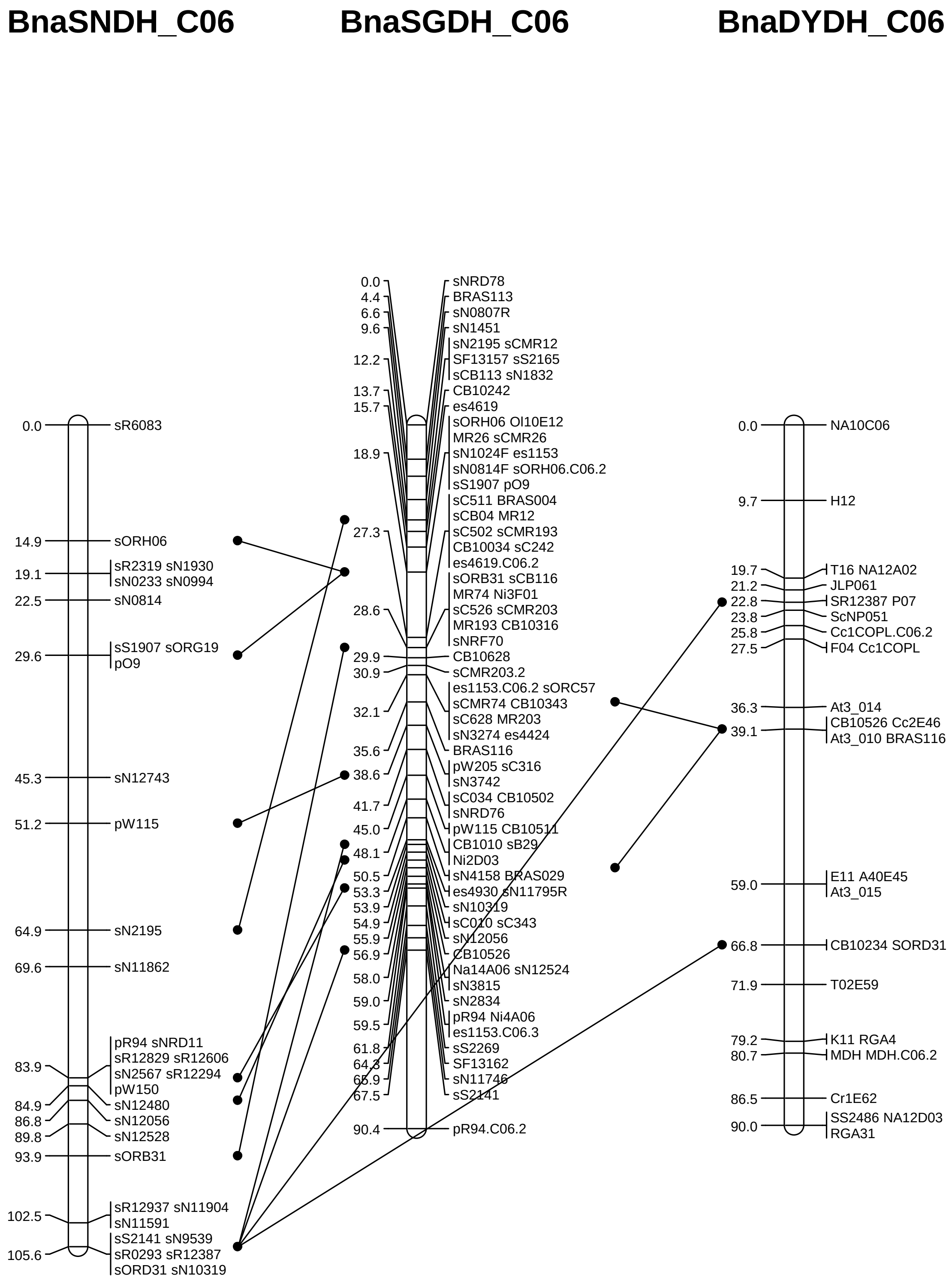
BnaSNDH_C06
BnaSGDH_C06
BnaDYDH_C06
0.0 sR6083
14.9 sORH06
19.1 sR2319 sN1930 sN0233 sN0994
22.5 sN0814
29.6 sS1907 sORG19 pO9
45.3 sN12743
51.2 pW115
64.9 sN2195
69.6 sN11862
83.9 pR94 sNRD11 sR12829 sR12606 sN2567 sR12294 pW150
84.9 sN12480
86.8 sN12056
89.8 sN12528
93.9 sORB31
102.5 sR12937 sN11904 sN11591
105.6 sS2141 sN9539 sR0293 sR12387 sORD31 sN10319
0.0 sNRD78
4.4 BRAS113
6.6 sN0807R
9.6 sN1451
12.2 sN2195 sCMR12 SF13157 sS2165 sCB113 sN1832
13.7 CB10242
15.7 es4619
18.9 sORH06 Ol10E12 MR26 sCMR26 sN1024F es1153 sN0814F sORH06.C06.2 sS1907 pO9
27.3 sC511 BRAS004 sCB04 MR12 sC502 sCMR193 CB10034 sC242 es4619.C06.2
28.6 sORB31 sCB116 MR74 Ni3F01 sC526 sCMR203 MR193 CB10316 sNRF70
29.9 CB10628
30.9 sCMR203.2
32.1 es1153.C06.2 sORC57 sCMR74 CB10343 sC628 MR203 sN3274 es4424
35.6 BRAS116
38.6 pW205 sC316 sN3742
41.7 sC034 CB10502 sNRD76
45.0 pW115 CB10511
48.1 CB1010 sB29 Ni2D03
50.5 sN4158 BRAS029
53.3 es4930 sN11795R
53.9 sN10319
54.9 sC010 sC343
55.9 sN12056
56.9 CB10526
58.0 Na14A06 sN12524 sN3815
59.0 sN2834
59.5 pR94 Ni4A06 es1153.C06.3
61.8 sS2269
64.3 SF13162
65.9 sN11746
67.5 sS2141
90.4 pR94.C06.2
0.0 NA10C06
9.7 H12
19.7 T16 NA12A02
21.2 JLP061
22.8 SR12387 P07
23.8 ScNP051
25.8 Cc1COPL.C06.2
27.5 F04 Cc1COPL
36.3 At3_014
39.1 CB10526 Cc2E46 At3_010 BRAS116
59.0 E11 A40E45 At3_015
66.8 CB10234 SORD31
71.9 T02E59
79.2 K11 RGA4
80.7 MDH MDH.C06.2
86.5 Cr1E62
90.0 SS2486 NA12D03 RGA31

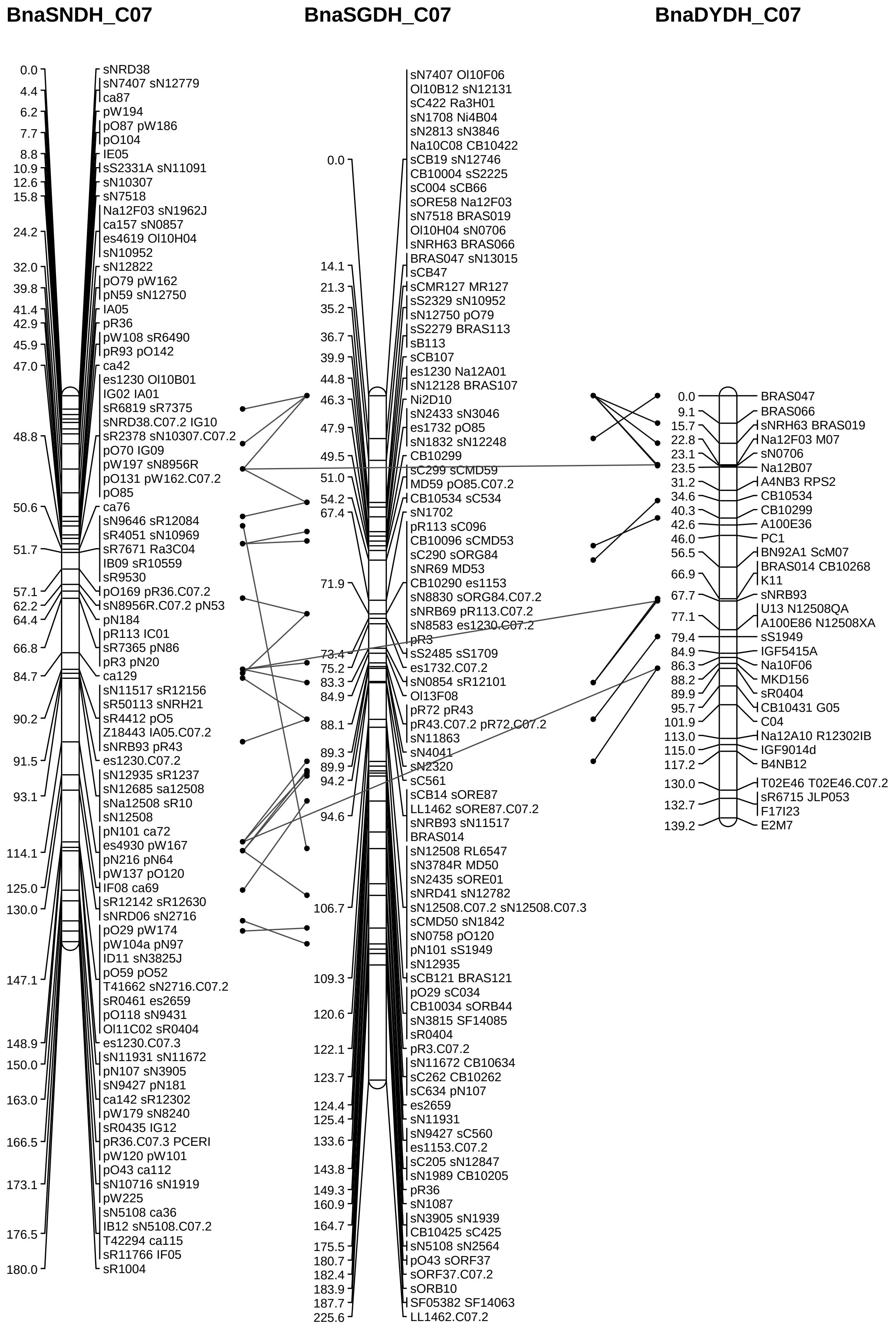
BnaSNDH_C07
BnaSGDH_C07
BnaDYDH_C07

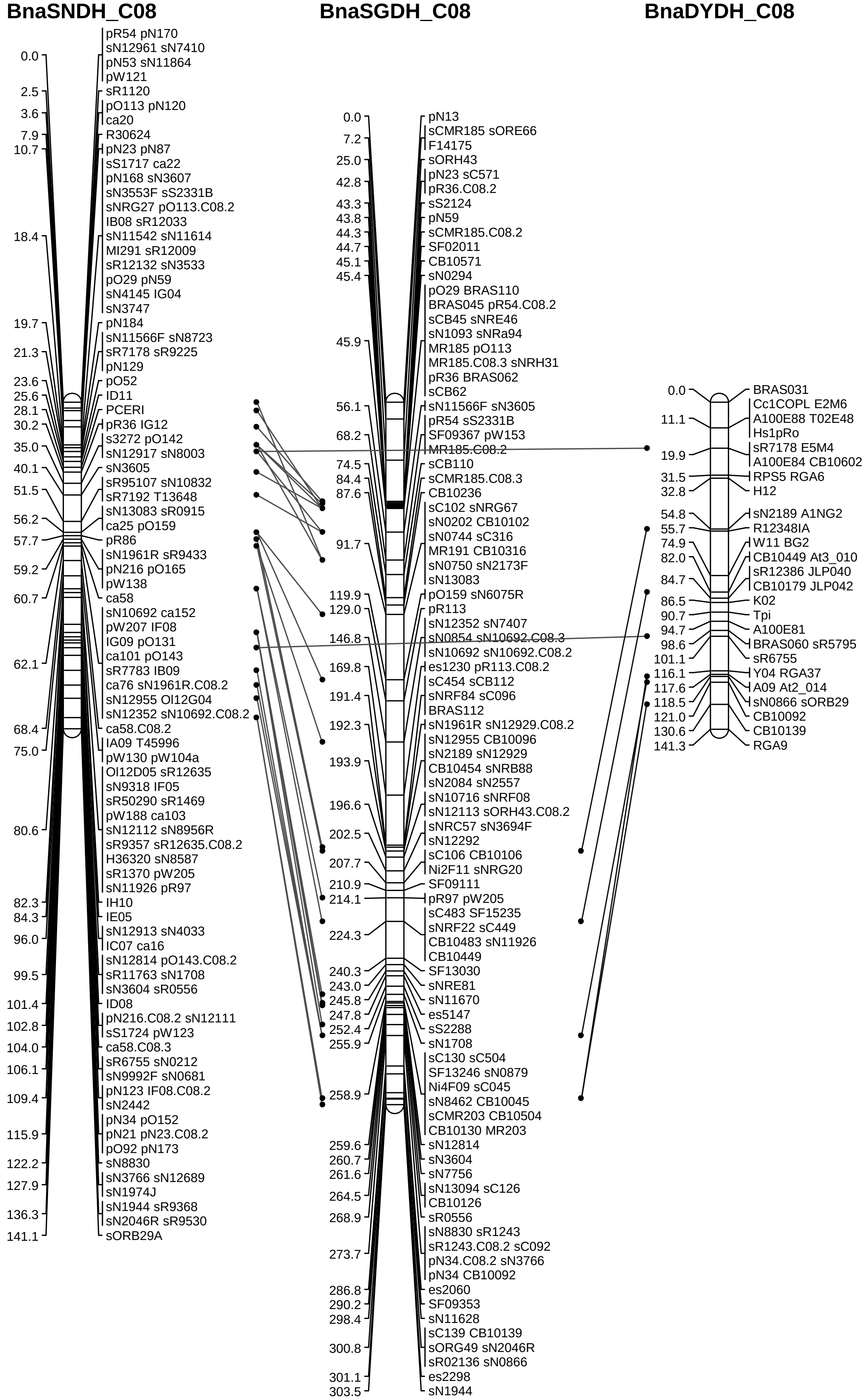
BnaSNDH_C08
0.0
2.5
3.6
7.9
10.7
18.4
19.7
21.3
23.6
25.6
28.1
30.2
35.0
40.1
51.5
56.2
57.7
59.2
60.7
62.1
68.4
75.0
80.6
82.3
84.3
96.0
99.5
101.4
102.8
104.0
106.1
109.4
115.9
122.2
127.9
136.3
141.1
pR54 pN170
sN12961 sN7410
pN53 sN11864
pW121
sR1120
pO113 pN120
ca20
R30624
pN23 pN87
sS1717 ca22
pN168 sN3607
sN3553F sS2331B
sNRG27 pO113.C08.2
IB08 sR12033
sN11542 sN11614
MI291 sR12009
sR12132 sN3533
pO29 pN59
sN4145 IG04
sN3747
pN184
sN11566F sN8723
sR7178 sR9225
pN129
pO52
ID11
PCERI
pR36 IG12
s3272 pO142
sN12917 sN8003
sN3605
sR95107 sN10832
sR7192 T13648
sN13083 sR0915
ca25 pO159
pR86
sN1961R sR9433
pN216 pO165
pW138
ca58
sN10692 ca152
pW207 IF08
IG09 pO131
ca101 pO143
sR7783 IB09
ca76 sN1961R.C08.2
sN12955 OI12G04
sN12352 sN10692.C08.2
ca58.C08.2
IA09 T45996
pW130 pW104a
OI12D05 sR12635
sN9318 IF05
sR50290 sR1469
pW188 ca103
sN12112 sN8956R
sR9357 sR12635.C08.2
H36320 sN8587
sR1370 pW205
sN11926 pR97
IH10
IE05
sN12913 sN4033
IC07 ca16
sN12814 pO143.C08.2
sR11763 sN1708
sN3604 sR0556
ID08
pN216.C08.2 sN12111
sS1724 pW123
ca58.C08.3
sR6755 sN0212
sN9992F sN0681
pN123 IF08.C08.2
sN2442
pN34 pO152
pN21 pN23.C08.2
pO92 pN173
sN8830
sN3766 sN12689
sN1974J
sN1944 sR9368
sN2046R sR9530
sORB29A
BnaSGDH_C08
0.0
7.2
25.0
42.8
43.3
43.8
44.3
44.7
45.1
45.4
45.9
56.1
68.2
74.5
84.4
87.6
91.7
119.9
129.0
146.8
169.8
191.4
192.3
193.9
196.6
202.5
207.7
210.9
214.1
224.3
240.3
243.0
245.8
247.8
252.4
255.9
258.9
259.6
260.7
261.6
264.5
268.9
273.7
286.8
290.2
298.4
300.8
301.1
303.5
pN13
sCMR185 sORE66
F14175
sORH43
pN23 sC571
pR36.C08.2
sS2124
pN59
sCMR185.C08.2
SF02011
CB10571
sN0294
pO29 BRAS110
BRAS045 pR54.C08.2
sCB45 sNRE46
sN1093 sNRa94
MR185 pO113
MR185.C08.3 sNRH31
pR36 BRAS062
sCB62
sN11566F sN3605
pR54 sS2331B
SF09367 pW153
MR185.C08.2
sCB110
sCMR185.C08.3
CB10236
sC102 sNRG67
sN0202 CB10102
sN0744 sC316
MR191 CB10316
sN0750 sN2173F
sN13083
pO159 sN6075R
pR113
sN12352 sN7407
sN0854 sN10692.C08.3
sN10692 sN10692.C08.2
es1230 pR113.C08.2
sC454 sCB112
sNRF84 sC096
BRAS112
sN1961R sN12929.C08.2
sN12955 CB10096
sN2189 sN12929
CB10454 sNRB88
sN2084 sN2557
sN10716 sNRF08
sN12113 sORH43.C08.2
sNRC57 sN3694F
sN12292
sC106 CB10106
Ni2F11 sNRG20
SF09111
pR97 pW205
sC483 SF15235
sNRF22 sC449
CB10483 sN11926
CB10449
SF13030
sNRE81
sN11670
es5147
sS2288
sN1708
sC130 sC504
SF13246 sN0879
Ni4F09 sC045
sN8462 CB10045
sCMR203 CB10504
CB10130 MR203
sN12814
sN3604
sN7756
sN13094 sC126
CB10126
sR0556
sN8830 sR1243
sR1243.C08.2 sC092
pN34.C08.2 sN3766
pN34 CB10092
es2060
SF09353
sN11628
sC139 CB10139
sORG49 sN2046R
sR02136 sN0866
es2298
sN1944
BnaDYDH_C08
0.0
11.1
19.9
31.5
32.8
54.8
55.7
74.9
82.0
84.7
86.5
90.7
94.7
98.6
101.1
116.1
117.6
118.5
121.0
130.6
141.3
BRAS031
Cc1COPL E2M6
A100E88 T02E48
Hs1pRo
sR7178 E5M4
A100E84 CB10602
RPS5 RGA6
H12
sN2189 A1NG2
R12348IA
W11 BG2
CB10449 At3_010
sR12386 JLP040
CB10179 JLP042
K02
Tpi
A100E81
BRAS060 sR5795
sR6755
Y04 RGA37
A09 At2_014
sN0866 sORB29
CB10092
CB10139
RGA9

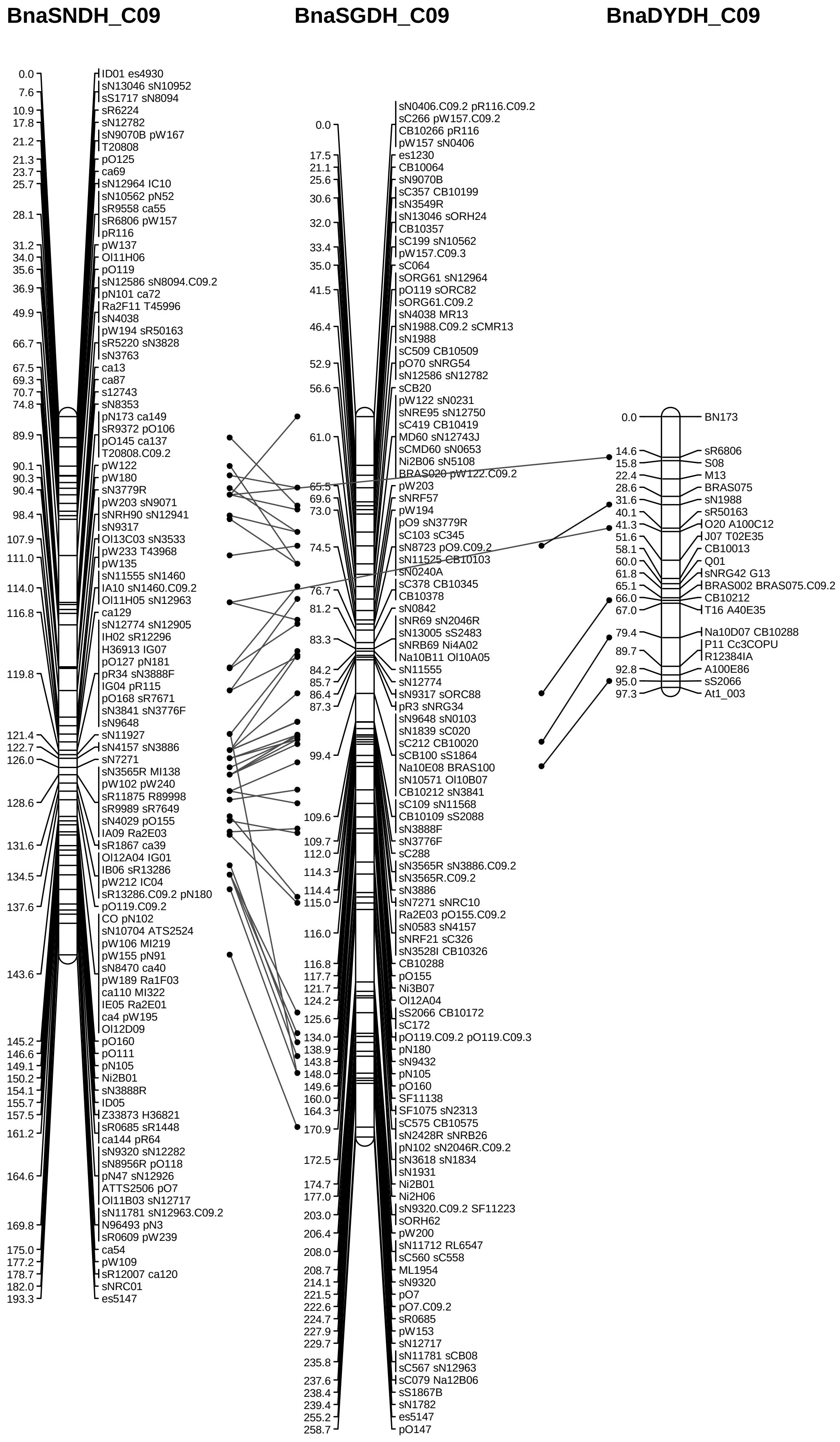

BnaSNDH_C09
BnaSGDH_C09
BnaDYDH_C09
