# Supplementary figures and images for: Integration of linkage maps for the Amphidiploid Brassica napus and comparative mapping with Arabidopsis and Brassica rapa
Source: BMC Genomics. 2011 Feb 9;12:101. doi: 10.1186/1471-2164-12-101 (PMC3042011; doi:10.1186/1471-2164-12-101)

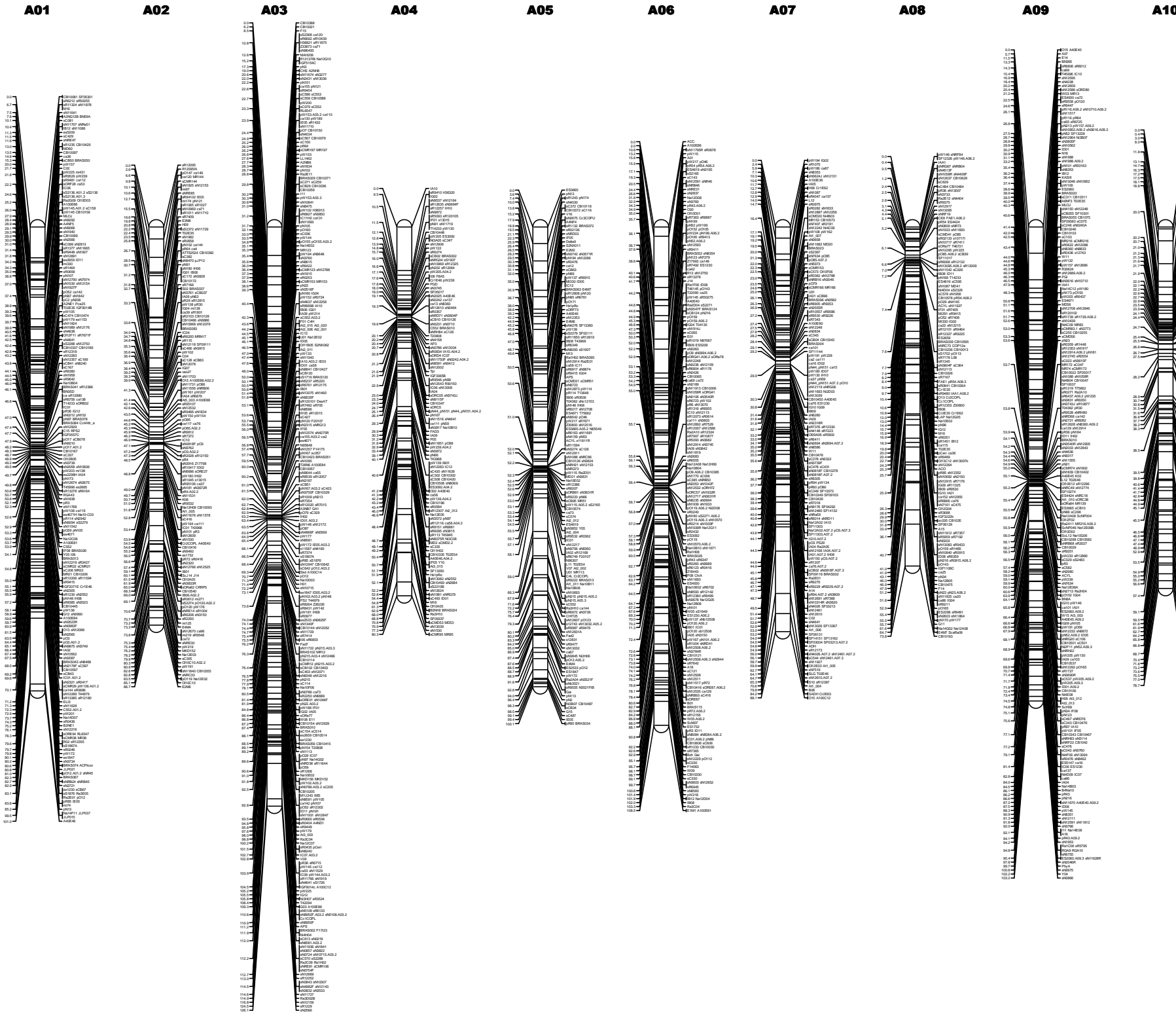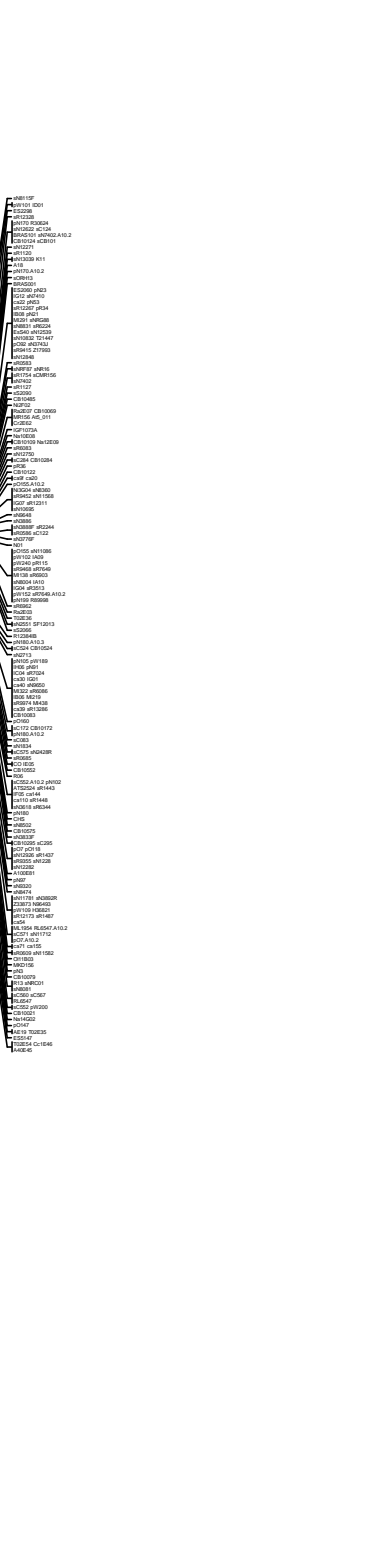

C01

C02

C03

C04

C05

C06

C07

C08

C09

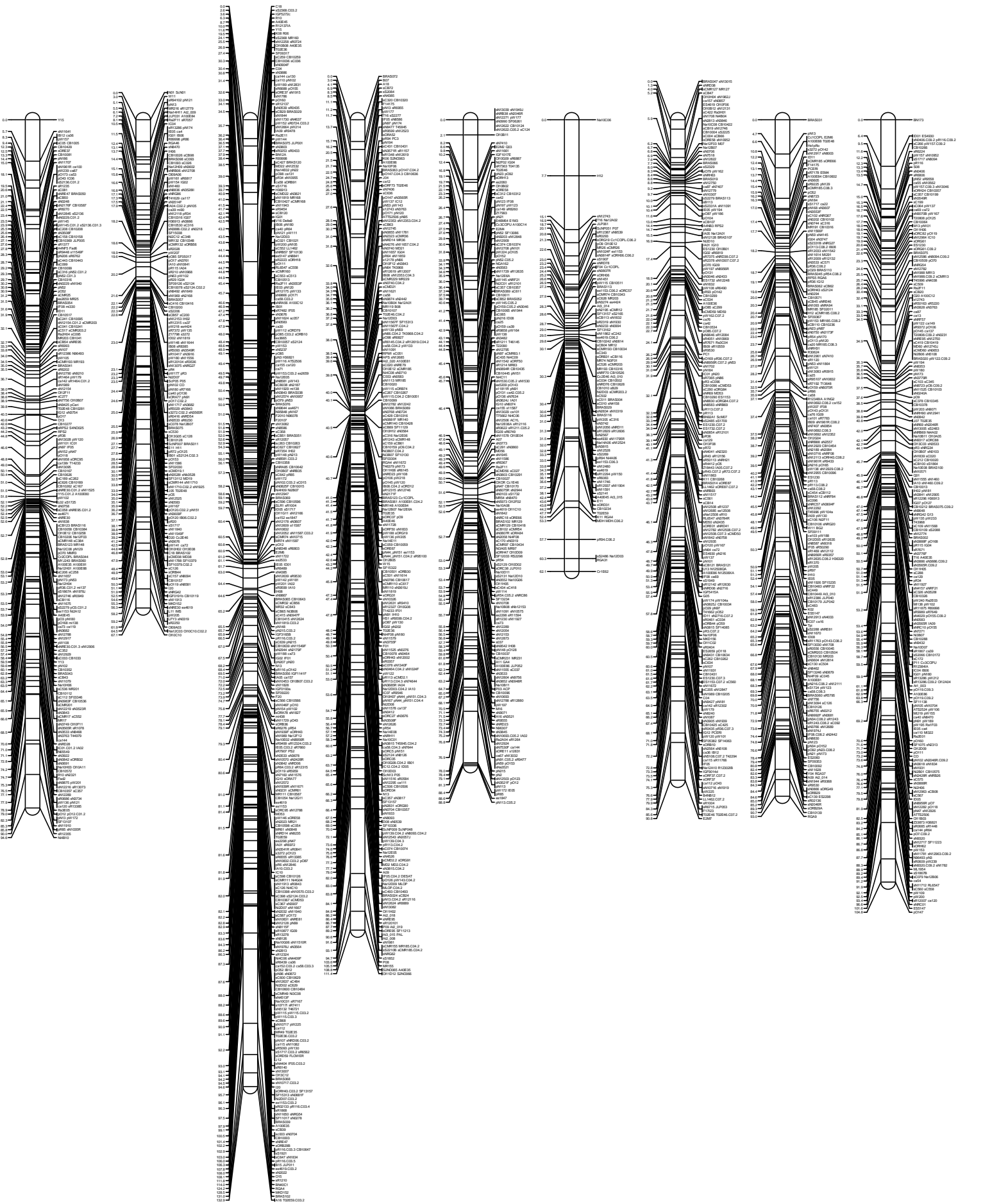

Supplement: Additional file 2 — The BnaWAIT_01_2010a integrated map (by JoinMap) generated by MapChart 2.1. [file 1471-2164-12-101-S2.PDF]
