# Supplementary material for: Integration of linkage maps for the Amphidiploid Brassica napus and comparative mapping with Arabidopsis and Brassica rapa
Source: BMC Genomics. 2011 Feb 9;12:101. doi: 10.1186/1471-2164-12-101 (PMC3042011; doi:10.1186/1471-2164-12-101)
Supplement: Additional file 4 — Dot-plots between the BnaWAIT map and all three population-specific maps for the remainder of all 19 LGs. The marker order of the vertical axis is from the BnaWAIT_01_2010a integrated map, and three marker orders of the horizontal axis are for the three population-specific maps. [file 1471-2164-12-101-S4.PDF]

Additional File 4. Dot-plots between the BnaWAIT map and all three population-specific maps for the remainder of all 19 LGs. The marker order of the vertical axis is from the BnaWAIT_01_2010a integrated map, and three marker orders of the horizontal axis are for the three population-specific maps.

(a)

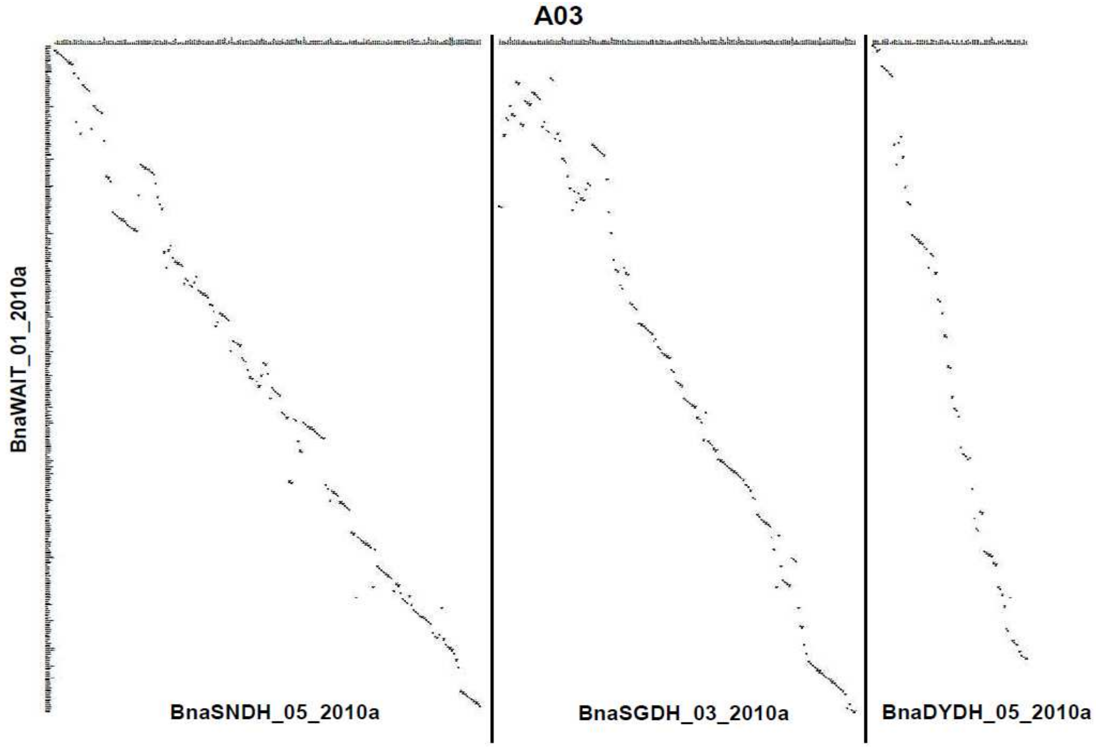

(b)

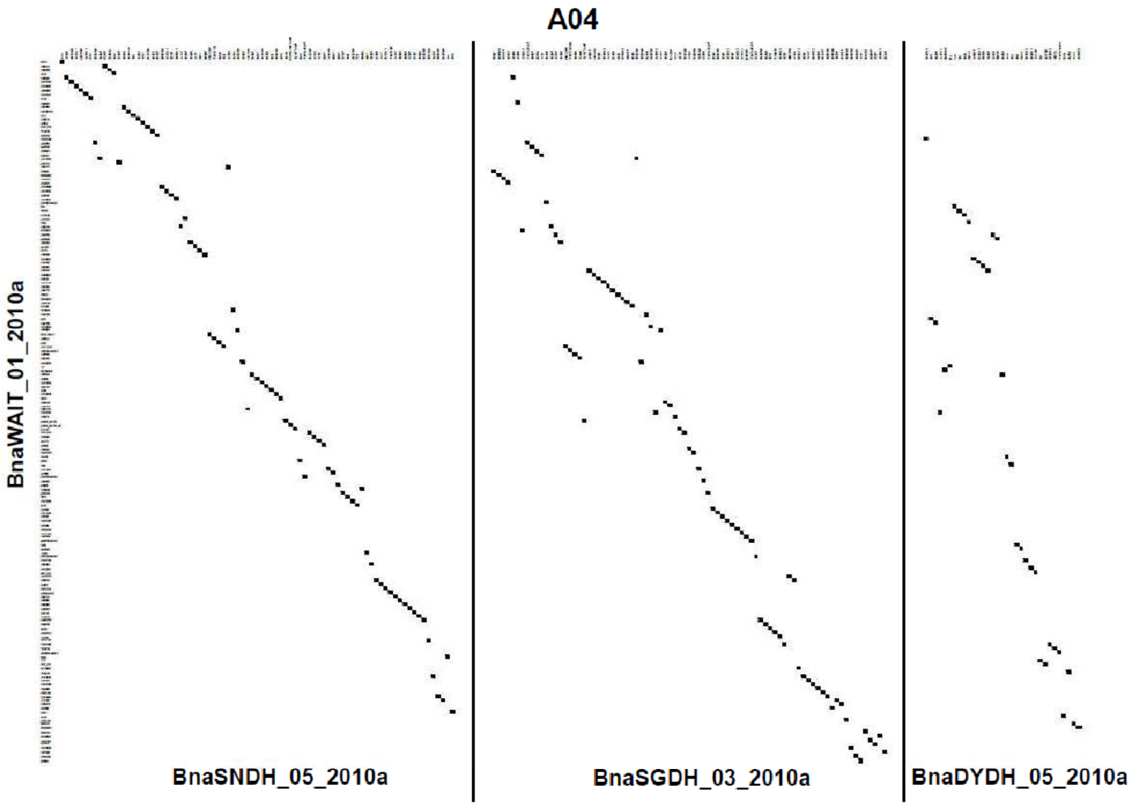
A04
BnaWAIT_01_2010a
BnaSNDH_05_2010a
BnaSGDH_03_2010a
BnaDYDH_05_2010a

(c)

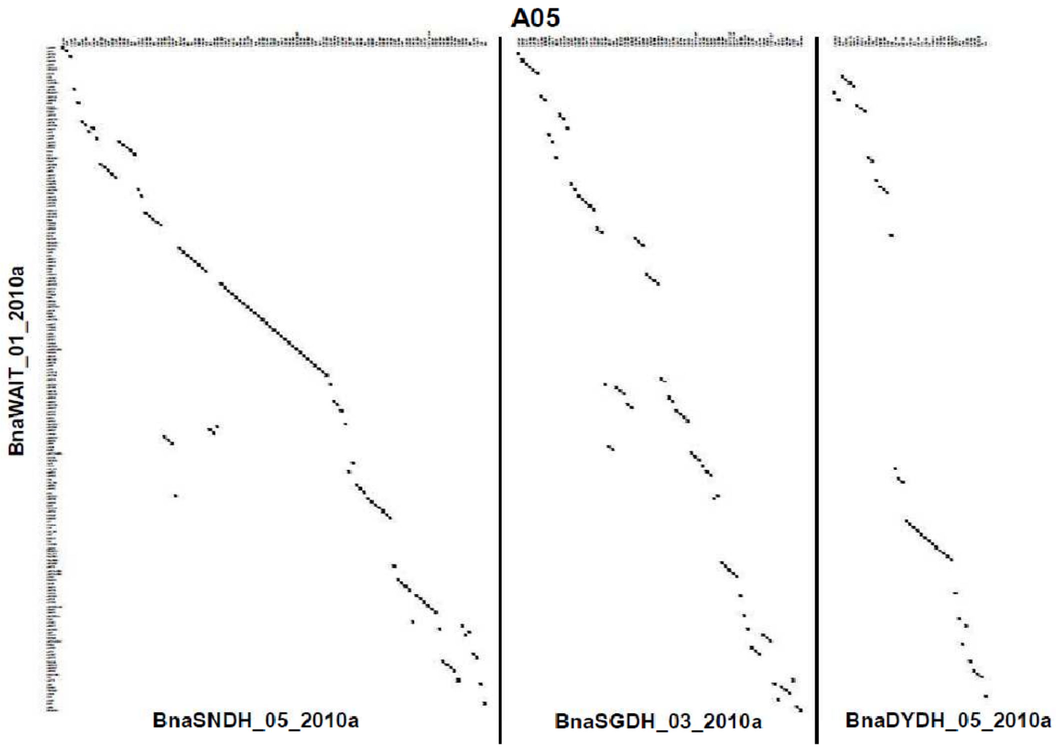
A05
BnaWAIT_01_2010a
BnaSNDH_05_2010a
BnaSGDH_03_2010a
BnaDYDH_05_2010a

(d)

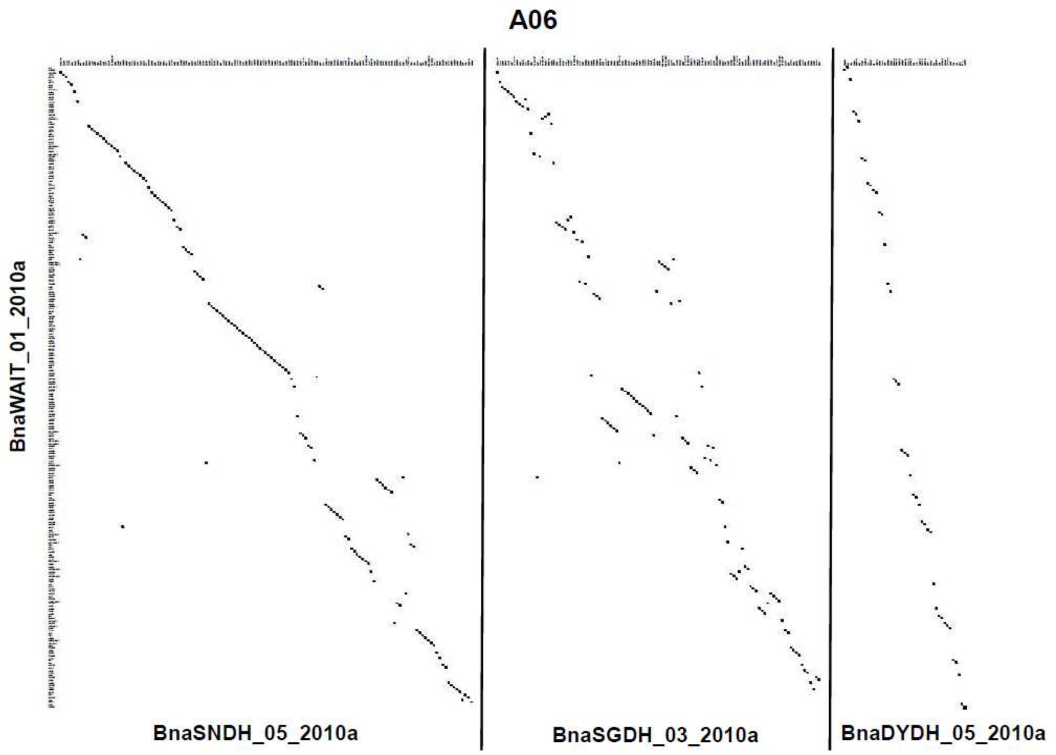
A06
BnaWAIT_01_2010a
BnaSNDH_05_2010a
BnaSGDH_03_2010a
BnaDYDH_05_2010a

(e)

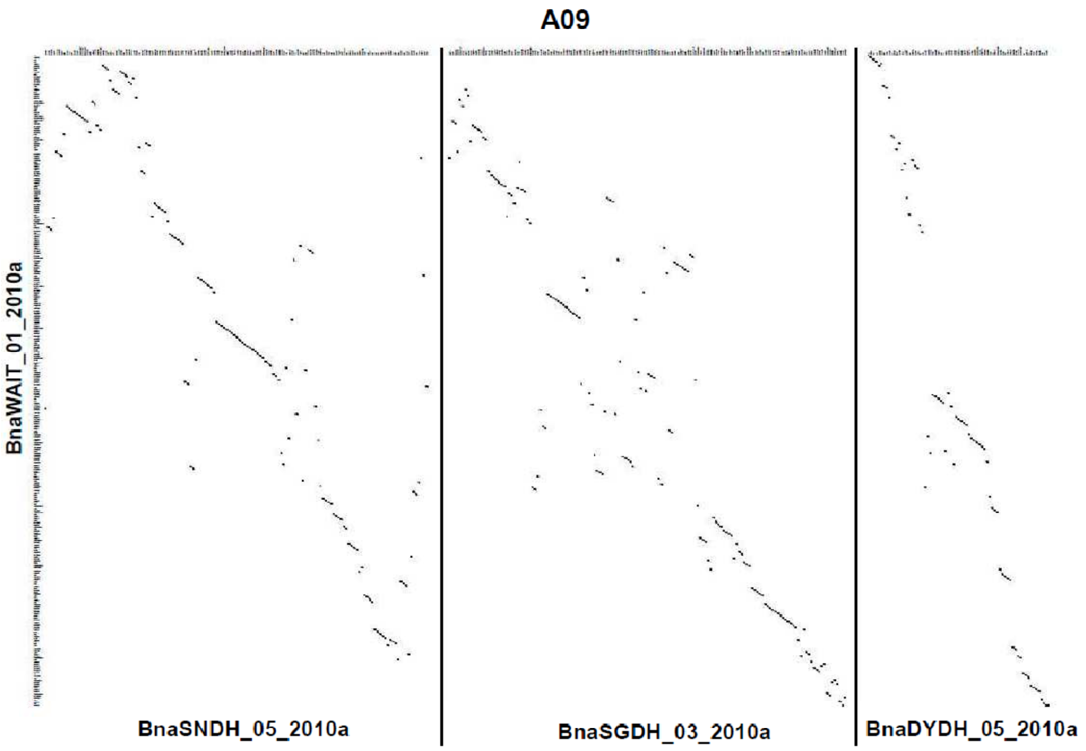
A09
BnaWAIT_01_2010a
BnaSNDH_05_2010a
BnaSGDH_03_2010a
BnaDYDH_05_2010a

(f)

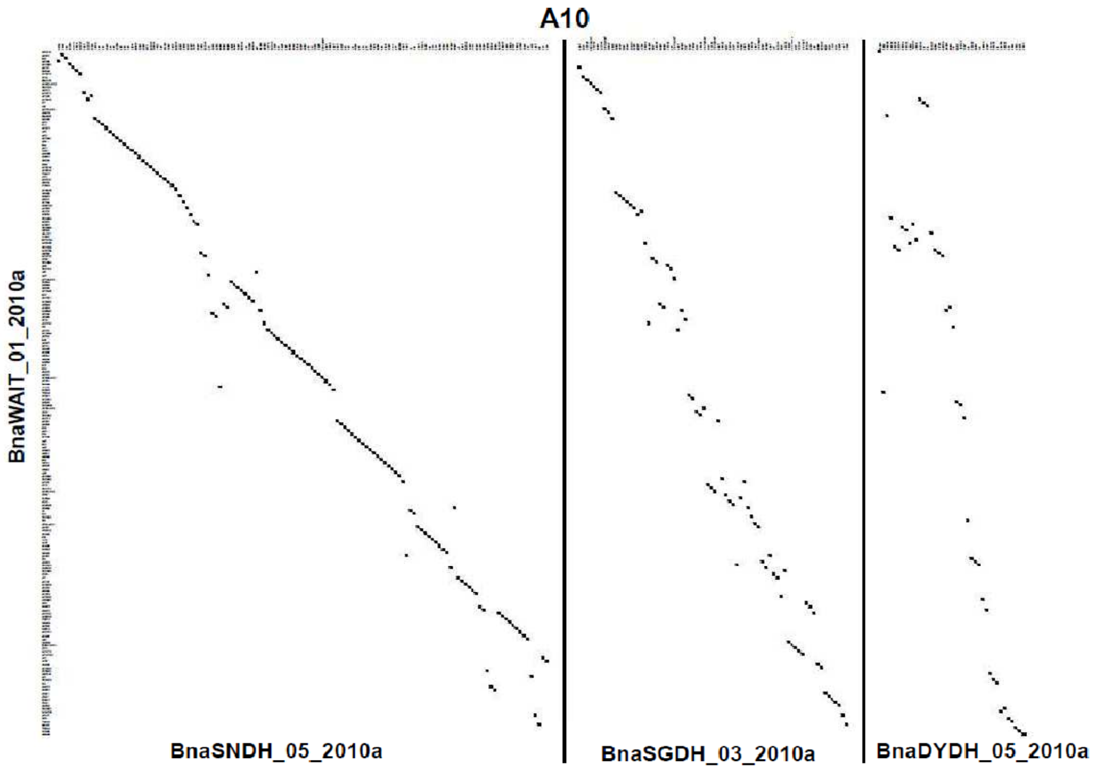
A10
BnaWAIT_01_2010a
BnaSNDH_05_2010a
BnaSGDH_03_2010a
BnaDYDH_05_2010a

(g)

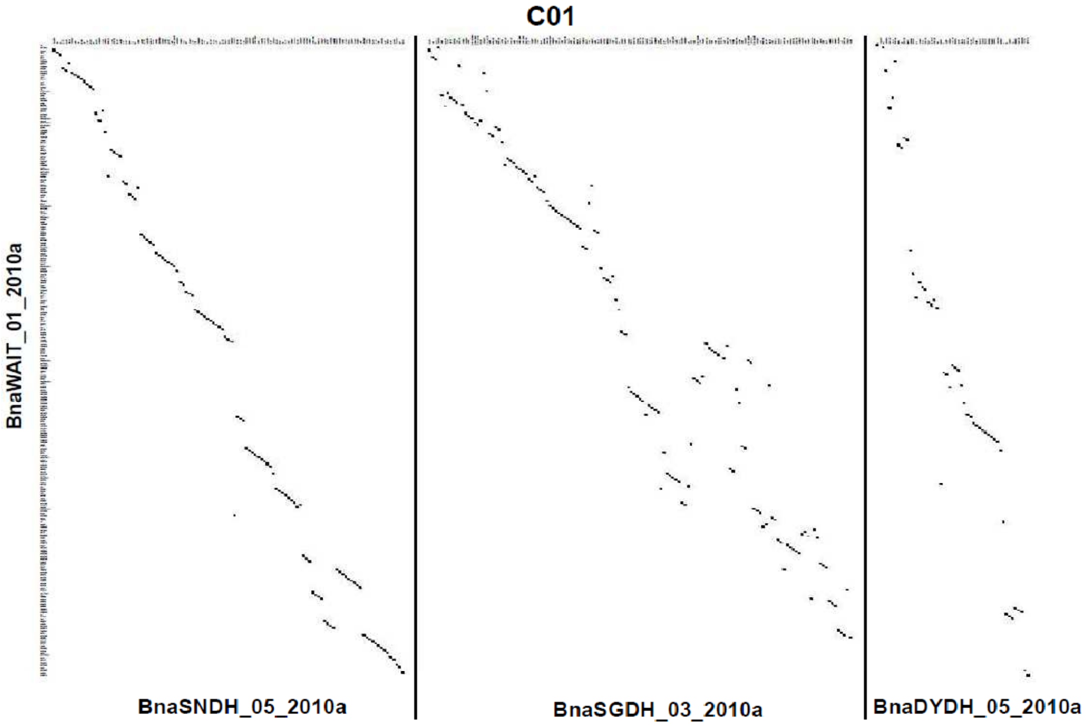
C01
BnaWAIT_01_2010a
BnaSNDH_05_2010a
BnaSGDH_03_2010a
BnaDYDH_05_2010a

(h)

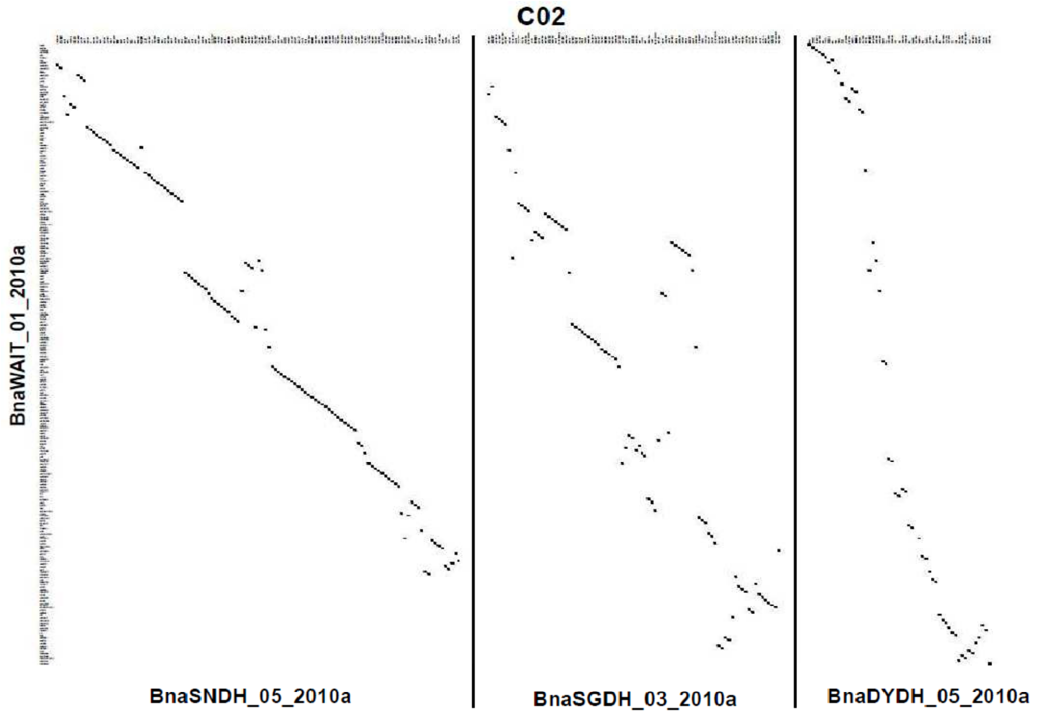
C02
BnaWAIT_01_2010a
BnaSNDH_05_2010a
BnaSGDH_03_2010a
BnaDYDH_05_2010a

(i)

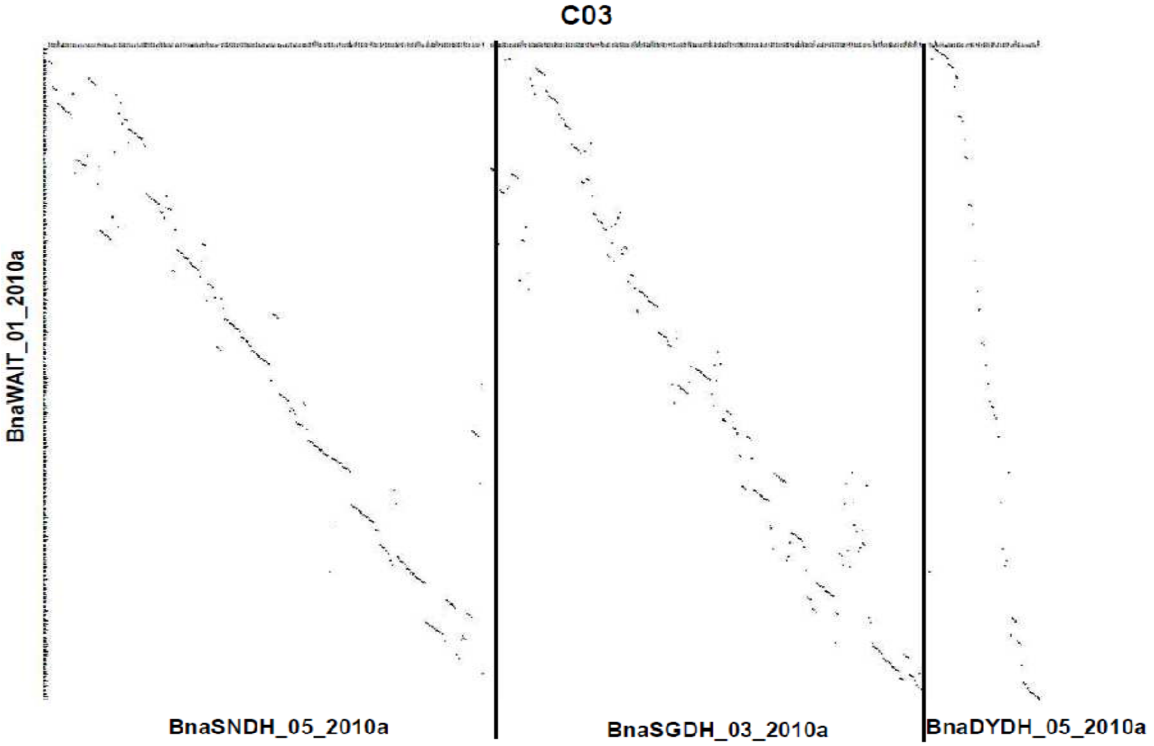
C03
BnaWAIT_01_2010a
BnaSNDH_05_2010a
BnaSGDH_03_2010a
BnaDYDH_05_2010a

(j)

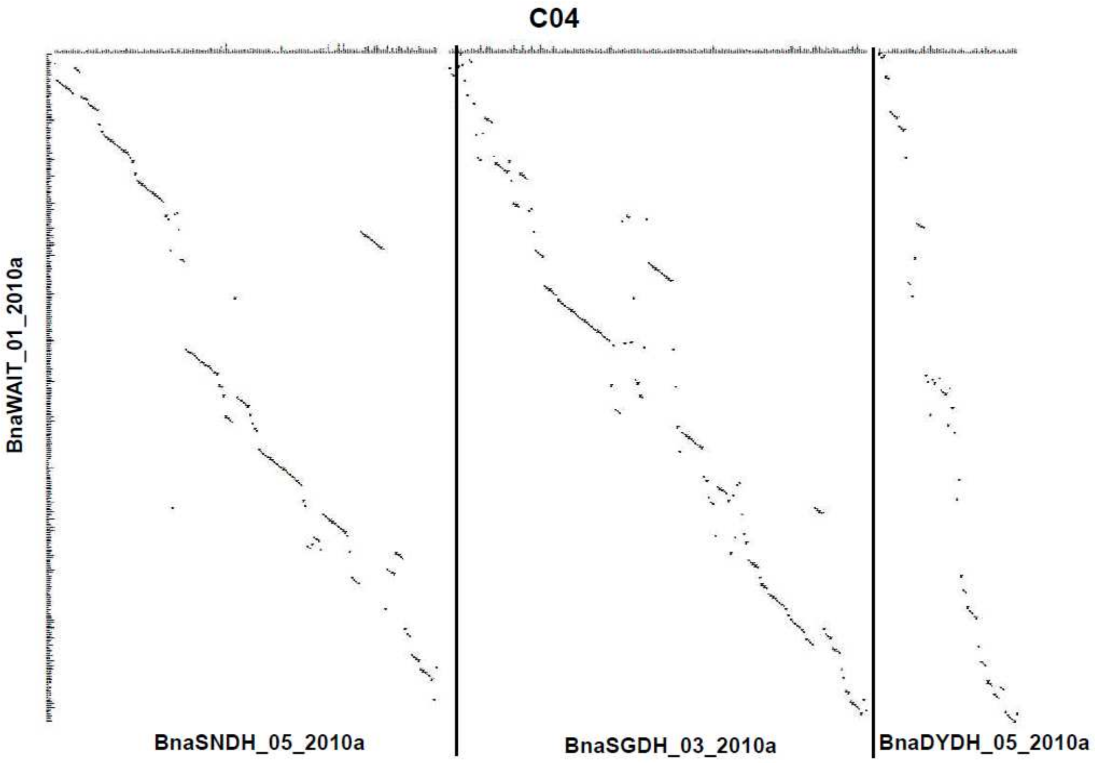
C04
BnaWAIT_01_2010a
BnaSNDH_05_2010a
BnaSGDH_03_2010a
BnaDYDH_05_2010a

(k)

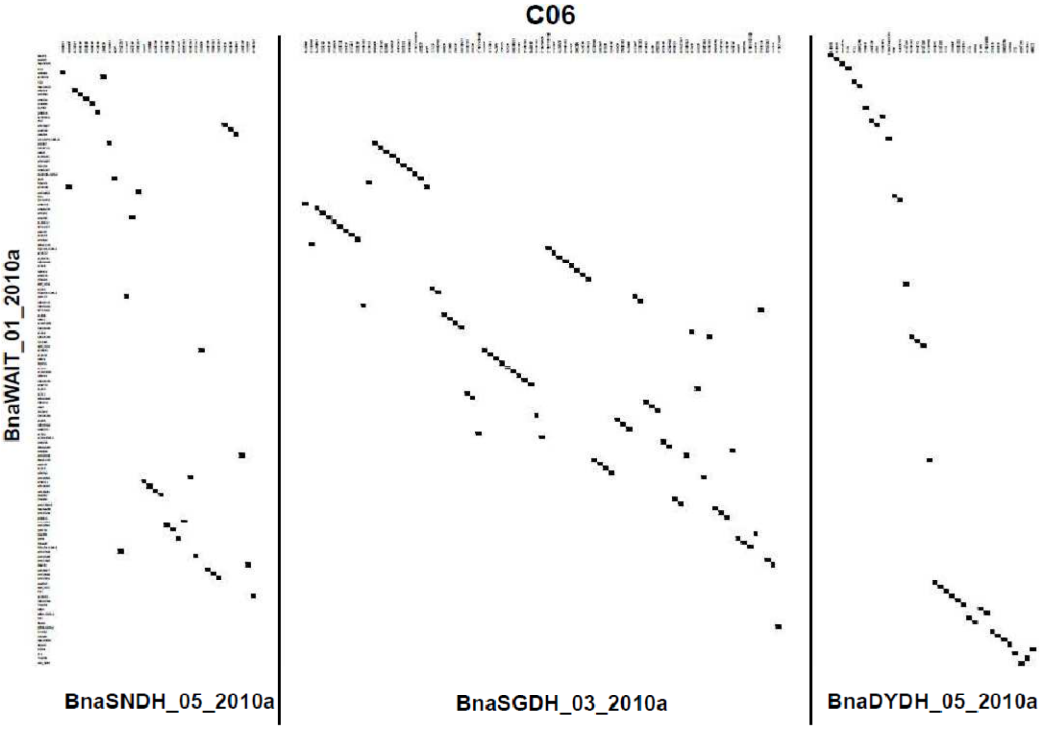
C06
BnaWAIT_01_2010a
BnaSNDH_05_2010a
BnaSGDH_03_2010a
BnaDYDH_05_2010a

(L)

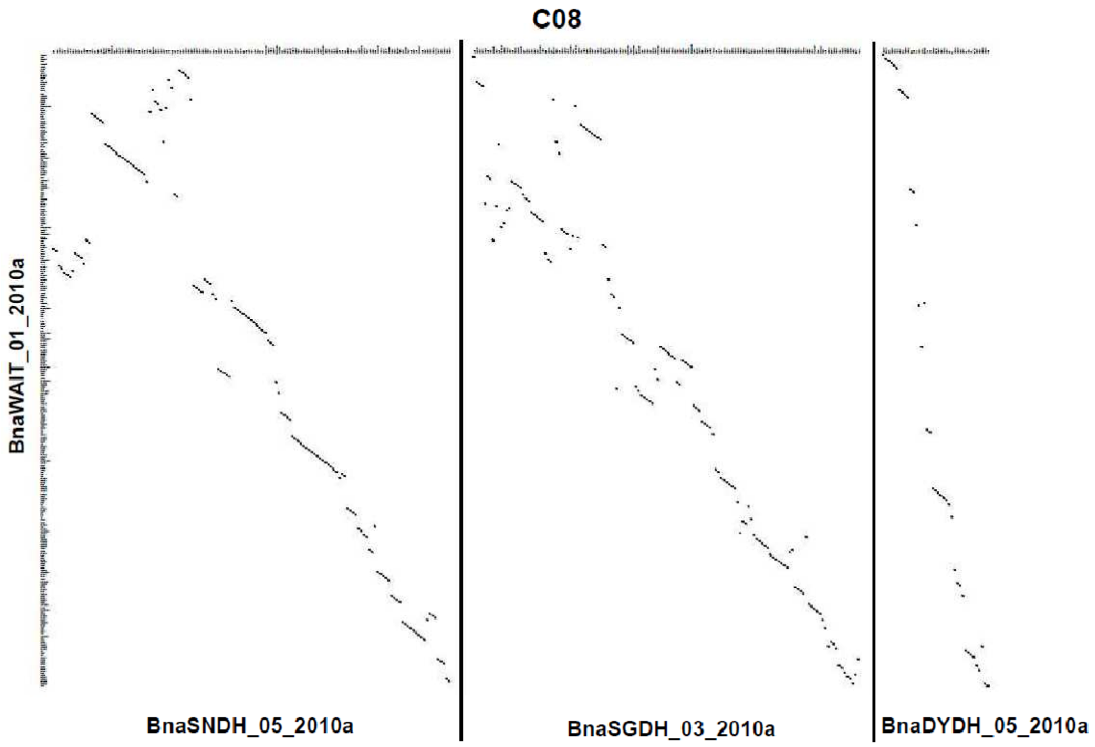
C08
BnaWAIT_01_2010a
BnaSNDH_05_2010a
BnaSGDH_03_2010a
BnaDYDH_05_2010a

(m)

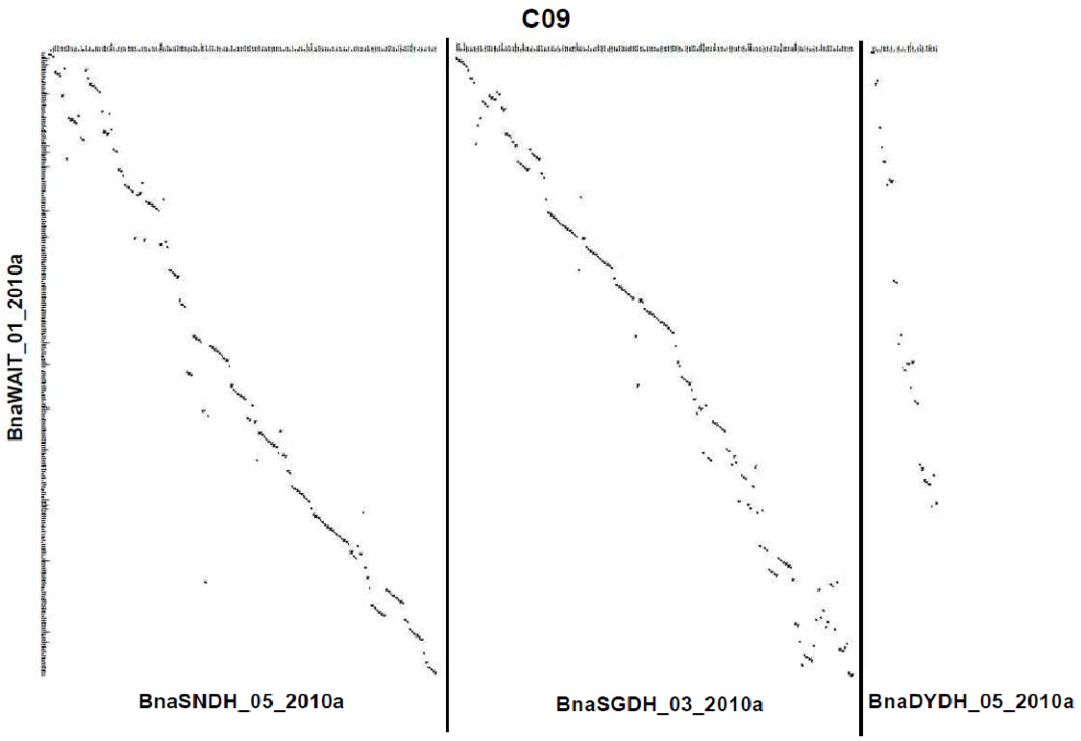
C09
BnaWAIT_01_2010a
BnaSNDH_05_2010a
BnaSGDH_03_2010a
BnaDYDH_05_2010a
