# Supplementary material for: Integration of linkage maps for the Amphidiploid Brassica napus and comparative mapping with Arabidopsis and Brassica rapa
Source: BMC Genomics. 2011 Feb 9;12:101. doi: 10.1186/1471-2164-12-101 (PMC3042011; doi:10.1186/1471-2164-12-101)
Supplement: Additional file 6 — Number of different sets of canonical marker assays using in the B. napus mapping, number of marker assays that show homology with Arabidopsis and number of homology regions in Arabidopsis with similarity to Brassica canonical marker assays. [file 1471-2164-12-101-S6.PDF]

Additional File 6. Number of different sets of canonical marker assays developed, number of marker assays that show homology with *Arabidopsis* and number of homology regions in *Arabidopsis* with similarity to *Brassica* canonical marker assays.

**Maker type**	**Marker set**	**Number**	**Number with hits in *Arabidopsis***	**Number of homology regions in *Arabidopsis***
RFLP	Osborn_lab_RFLPs_2	482	235	296
SSR	BBSRC_SSRs	858	99	140
SSR	Celera_SSRs	361	150	280
SSR	AAFC_SSRs	3257	311	440
