# Supplementary material for: Integration of linkage maps for the Amphidiploid Brassica napus and comparative mapping with Arabidopsis and Brassica rapa
Source: BMC Genomics. 2011 Feb 9;12:101. doi: 10.1186/1471-2164-12-101 (PMC3042011; doi:10.1186/1471-2164-12-101)
Supplement: Additional file 7 — Summary of conserved collinearity blocks between the B. napus integrated map BnaWAIT_01_2010a and the Arabidopsis genome sequence. [file 1471-2164-12-101-S7.PDF]

Additional File 7. Summary of conserved collinearity blocks between the *B. napus* integrated map BnaWAIT_01_2010a and the *Arabidopsis* genome sequence.

		B. napus **(BnaWAIT_01_2010a)**				***Arabidopsis thaliana*** **(TAIR9)**					**Corresponding blocks identified by**	
**Block**	**LG**	**Start (cM)**	**End (cM)**	**length (cM)**	**Number of supporting markers**	**Chromosome**	**Start (Mb)**	**End (Mb)**	**length(Mb)**	**Spearman's correlation®**	**Parkin *et al.* 2005**	**Schranz *et al.* 2005**
[2]***BnaWAIT_A_01***	**A01**	0.0	29.9	29.9	17	4	14.2	18.5	4.3	-0.92****	C4B	U
[2]***BnaWAIT_A_02***		30.0	43.8	13.8	17	4	10.2	12.7	2.5	0.72***	C4B	U
[2]***BnaWAIT_A_03***		44.5	47.0	3.5	6	4	8.0	9.7	1.7	-0.47	C4B'	T, U
BnaWAIT_A_04		52.6	55.5	2.9	7	3	7.4	8.3	0.9	0.41	C3A	F
BnaWAIT_A_05		57.2	83.8	26.6	27	3	0.4	6.5	6.1	-0.74	C3A	F
BnaWAIT_A_06	**A02**	6.6	15.5	8.9	6	5	0.5	2.7	2.2	0.79*	C5A	R
BnaWAIT_A_07		22.1	31.2	9.1	16	5	4.4	6.9	2.5	0.94****	C5A	R
BnaWAIT_A_08		32.7	34.8	2.1	11	5	23.6	24.4	0.8	-0.30	C5E	W
BnaWAIT_A_09		41.3	49.0	7.7	18	1	24.2	30.3	6.1	0.57*	C1E	E
BnaWAIT_A_10		69.1	73.8	4.7	5	5	8.6	9.8	1.2	-0.57	C5B	Q
BnaWAIT_A_11		75.4	85.1	9.7	6	5	25.5	26.9	1.4	-0.17	C5F	X
BnaWAIT_A_12	**A03**	0.0	31.7	31.7	29	5	2.0	5.7	3.7	0.77****	C5A	R
BnaWAIT_A_13		31.2	40.4	6.9	10	5	21.7	24.3	2.6	0.49	C5E	W
BnaWAIT_A_14		40.4	56.5	16.1	12	2	13.0	16.6	3.6	0.71**	C2C	J
BnaWAIT_A_15		60.2	65.4	5.2	8	4	0.2	2.7	2.5	0.50	C4A	O
BnaWAIT_A_16		66.1	77.8	11.7	25	3	0.4	6.4	6.0	0.59**	C3A	F
BnaWAIT_A_17		80.5	88.1	7.6	4	3	7.6	10.6	3.0	0.95*	C3A	F
BnaWAIT_A_18		88.1	111.9	23.8	25	4	9.1	16.9	7.8	0.89****	C4B	U
BnaWAIT_A_19	**A04**	10.5	12.8	2.3	8	3	19.8	21.3	1.5	0.10	C3D	N
BnaWAIT_A_20		20.8	54.4	33.6	31	2	8.2	19.1	10.9	0.86****	C2B, C2C	H, I, J
BnaWAIT_A_21	**A05**	29.2	30.8	1.6	4	2	18.0	18.7	0.7	-0.74	C2C	J
BnaWAIT_A_22		35.0	36.7	1.7	5	2	15.2	16.3	1.1	0.63	C2C	J
BnaWAIT_A_23		45.2	50.5	5.3	5	2	13.3	14.9	1.6	-0.87*	C2C	J
BnaWAIT_A_24		50.5	51.0	0.5	5	1	11.4	13.6	2.2	-0.35	C1B	B
BnaWAIT_A_25		50.5	51.0	0.5	6	1	17.7	19.2	1.5	0.65	C1C	C
BnaWAIT_A_26		51.0	100.1	49.1	30	3	0.4	8.0	7.6	-0.92****	C3A	F
BnaWAIT_A_27	**A06**	13.2	20.5	7.3	8	1	18.1	19.5	1.4	-0.33	C1C	C
BnaWAIT_A_28		36.1	38.6	2.5	11	1	3.1	4.4	1.3	0.71**	C1A	A
BnaWAIT_A_29		44.2	55.0	10.8	19	1	5.2	7.3	2.1	0.78***	C1A, C1B	A, B
[1]**BnaWAIT_A_30**		54.9	56.4	1.5	5	3	15.5	18.4	2.9	-0.95**	C3C	M
BnaWAIT_A_31		55.7	60.0	4.3	13	5	24.1	25.9	1.8	-0.22	C5F	X
BnaWAIT_A_32		67.3	72.8	5.5	8	5	9.0	9.9	0.9	-0.42	C5B	Q
BnaWAIT_A_33		90.8	103.2	12.5	12	5	17.2	19.3	2.1	-0.88****	C5D	V
BnaWAIT_A_34	**A07**	0.0	16.4	16.4	7	2	5.7	8.9	3.2	-0.87**	C2A	H
BnaWAIT_A_35		17.1	26.1	11.0	10	1	7.5	10.9	3.4	-0.22	C1B	B
BnaWAIT_A_36		26.9	29.9	3.0	5	2	11.5	12.4	0.9	-0.36	C2B	I
BnaWAIT_A_37		29.9	34.0	3.1	7	3	18.5	22.1	3.6	0.49	C3D	M
BnaWAIT_A_38		29.9	43.6	13.7	21	1	26.8	30.2	3.4	-0.63**	C1E	E

BnaWAIT_A_39		44.4	60.0	15.6	17	1	25.1	28.3	3.2	0.66**	C1E	E
BnaWAIT_A_40	**A08**	7.2	10.6	3.4	8	1	17.1	19.0	1.9	0.41	C1C	C
BnaWAIT_A_41		7.1	7.4	0.3	11	4	7.5	10.1	2.6	0.32	C4B	T, U
BnaWAIT_A_42		18.9	25.9	7.0	4	4	16.8	18.0	1.2	-0.21	C4B	U
BnaWAIT_A_43		25.7	43.1	17.4	13	1	5.4	8.3	2.9	-0.44	C1A, C1B	A, B
BnaWAIT_A_44		51.2	55.5	4.3	7	1	0.5	3.2	2.7	-0.68*	C1A	A
BnaWAIT_A_45	**A09**	41.4	48.3	6.9	9	1	21.4	24.0	2.6	-0.45	C1D	D
BnaWAIT_A_46		53.7	59.0	5.3	11	1	10.6	12.1	1.5	-0.50	C1B	B
BnaWAIT_A_47		53.9	56.5	2.6	4	5	17.7	19.0	1.3	0.63	C5D	V
BnaWAIT_A_48		59.9	66.6	6.7	6	1	8.3	9.3	1.0	-0.17	C1B	B
BnaWAIT_A_49		66.4	81.4	15.0	22	3	19.5	23.2	3.7	0.85****	C3D	N
BnaWAIT_A_50		81.4	92.1	10.7	6	2	9.0	10.6	1.6	-0.45	C2B	I
BnaWAIT_A_51	**A10**	0.4	11.8	11.4	4	1	0.0	1.4	1.4	0.26	C1A	A
BnaWAIT_A_52		17.5	20.0	2.5	6	1	1.4	2.2	0.8	0.65	C1A	A
BnaWAIT_A_53		21.9	28.2	6.3	20	5	21.0	24.3	3.3	0.80****	C5E	W
BnaWAIT_A_54		28.2	71.8	43.6	42	5	1.9	7.3	5.4	-0.91****	C5A	R
[2]***BnaWAIT_C_01***	**C01**	8.3	30.2	21.9	20	4	14.2	18.5	4.3	-0.70***	C4B	U
[2]***BnaWAIT_C_02***		30.9	44.8	13.9	16	4	9.9	13.6	3.7	0.85****	C4B	U
[2]***BnaWAIT_C_03***		46.6	49.0	3.4	6	4	8.0	9.7	1.7	-0.56	C4B'	T, U
BnaWAIT_C_04		52.4	55.8	3.4	4	3	7.4	8.3	0.9	0.32	C3A	F
BnaWAIT_C_05		63.5	85.8	22.3	21	3	0.4	6.4	6.0	-0.84****	C3A	F
BnaWAIT_C_06	**C02**	5.1	13.7	8.6	10	5	4.3	7.6	3.3	0.56	C5A	R
BnaWAIT_C_07		16.6	20.2	3.6	12	5	20.5	24.3	3.8	0.90****	C5E	W
BnaWAIT_C_08		16.6	23.0	6.4	12	1	24.2	30.2	6.0	0.78***	C1E	E
BnaWAIT_C_09		25.0	34.0	9.0	5	3	9.5	10.0	0.5	0.22	C3B	L
BnaWAIT_C_10		59.1	67.1	8.0	4	5	24.9	26.1	1.2	0.95*	C5F	X
BnaWAIT_C_11	**C03**	31.4	33.0	1.6	6	5	4.5	5.5	1.0	0.65	C5A	R
[2]***BnaWAIT_C_12***		32.6	36.4	3.8	6	5	22.5	24.1	1.6	0.62	C5E	W
[2]***BnaWAIT_C_13***		39.0	41.0	2.0	4	5	20.7	22.2	1.5	-0.32	C5E	W
BnaWAIT_C_14		42.4	44.5	2.1	8	5	4.3	6.7	2.4	-0.41	C5A	R
BnaWAIT_C_15		44.4	49.7	5.3	15	2	14.5	17.6	3.1	0.57*	C2C	J
[1]**BnaWAIT_C_16**		48.6	49.5	0.9	8	5	0.7	2.8	2.1	0.61	C5A	R
BnaWAIT_C_17		55.1	59.2	4.1	5	4	0.1	0.9	0.8	0.11	C4A	O
BnaWAIT_C_18		60.4	67.0	6.6	18	3	2.2	6.4	4.2	0.38	C3A	F
[1]**BnaWAIT_C_19**		75.8	81.8	6.0	7	5	25.0	26.0	1.0	-0.71*	C5F	X
BnaWAIT_C_20		81.6	84.3	2.7	6	3	16.4	18.4	2.0	0.43	C3C	M
[2]***BnaWAIT_C_21***		87.3	97.5	10.2	5	4	10.0	11.8	1.8	0.90*	C4B	U
[2]***BnaWAIT_C_22***		87.3	108.0	20.7	12	4	15.1	18.0	2.9	-0.44	C4B	U
BnaWAIT_C_23	**C04**	11.1	40.0	29.9	26	2	13.0	19.7	6.7	-0.83****	C2C	J
BnaWAIT_C_24		40.5	42.6	2.1	5	1	23.0	23.8	0.8	0.71	C1D	D
BnaWAIT_C_25		40.7	47.8	7.1	7	3	19.5	21.7	2.2	-0.73	C3D	N
BnaWAIT_C_26		50.4	77.3	26.9	12	2	9.1	17.0	7.9	0.44*	C2B, C2C	I,J
BnaWAIT_C_27	**C05**	8.2	19.7	11.5	8	1	2.0	2.5	0.5	0.29	C1A	A
BnaWAIT_C_28		26.2	31.8	5.6	9	1	1.8	4.8	3.0	-0.62	C1A	A
BnaWAIT_C_29		39.7	53.3	13.6	37	1	3.1	11.6	8.5	0.87****	C1A, C1B	A, B
BnaWAIT_C_30		54.8	88.8	34.0	24	3	0.4	7.6	7.2	-0.88****	C3A	F
BnaWAIT_C_31	**C06**	26.9	29.8	8.3	5	1	18.7	20.7	2.0	0.40	C1C	C
[1]**BnaWAIT_C_32**		30.3	32.0	1.7	6	3	20.1	22.8	2.7	-0.14	C3D	N
BnaWAIT_C_33	**C07**	5.9	14.7	8.8	7	2	7.2	8.9	1.7	0.62	C2A	H

BnaWAIT_C_34		17.4	22.7	5.3	11	1	7.1	9.2	2.1	-0.65*	C1B	B
BnaWAIT_C_35		24.1	32.1	8.0	10	5	17.8	19.6	1.8	0.82*	C5D	V
BnaWAIT_C_36		41.1	42.5	1.4	10	3	9.3	10.4	1.1	0.44	C3B	L
BnaWAIT_C_37		47.8	51.3	3.5	8	5	8.0	9.8	1.8	-0.16	C5B	Q
[1]**BnaWAIT_C_38**		53.4	57.8	4.4	4	3	18.9	19.4	0.5	-0.25	C3D	N
[2]***BnaWAIT_C_39***		57.8	68.1	10.3	15	4	9.1	13.6	4.5	0.96****	C4B	U
[2]***BnaWAIT_C_40***		72.2	75.3	3.2	8	4	14.8	18.0	3.2	-0.87**	C4B	U
BnaWAIT_C_41	**C08**	18.7	26.7	8.0	6	1	1.0	2.0	1.0	0.40	C1A	A
BnaWAIT_C_42		28.2	44.2	16.0	14	1	5.4	9.4	4.0	0.42	C1A, C1B	A, B
BnaWAIT_C_43		45.5	62.8	17.3	20	3	18.5	23.2	4.7	0.75***	C3D	N
BnaWAIT_C_44		65.2	78.4	13.2	16	1	1.8	6.7	4.9	-0.89****	C1A	A
BnaWAIT_C_45	**C09**	6.4	14.7	8.3	6	4	0.1	1.4	1.3	0.60	C4A	O
BnaWAIT_C_46		16.7	19.1	2.4	7	5	7.8	9.9	2.1	-0.46	C5B	Q
BnaWAIT_C_47		34.9	38.8	3.9	8	1	21.5	24.0	2.5	0.46	C1D	D
BnaWAIT_C_48		45.7	55.4	9.7	21	5	21.1	24.5	3.4	0.72***	C5E	W
BnaWAIT_C_49		58.7	104.8	46.1	44	5	0.5	6.7	6.2	-0.88****	C5A	R

[1] New blocks indentified in the BnaWAIT map in relation to Parkin et al. (2005) are highlighted in bold.

[2] Inversions identified in *Brassica* relative to *Arabidopsis* are indicated in *Italic*.

® Spearman's rank correlation (*r*) indicates the order conservation and orientation for each block of *B. napus* relative to *Arabidopsis*. Significance is shown as: * < 0.05; ** < 0.01; *** < 0.001; **** < 0.0001.
