# Supplementary material for: Integration of linkage maps for the Amphidiploid Brassica napus and comparative mapping with Arabidopsis and Brassica rapa
Source: BMC Genomics. 2011 Feb 9;12:101. doi: 10.1186/1471-2164-12-101 (PMC3042011; doi:10.1186/1471-2164-12-101)
Supplement: Additional file 8 — Description of mapping populations and genetic markers used in the map integration study, which are maintained in CropStoreDB. Corresponding references are also shown if available. [file 1471-2164-12-101-S8.PDF]

Additional File 8. Description of mapping populations and genetic markers used in the map integration study, which are maintained in CropStoreDB. Corresponding references are also shown if available.

Population original name	Registry population name*	Genetic marker description$	Marker prefix	Reference	Registry genetic map name**	Number of lines in the merged matrix (n)
N-fo-61-9	BnaSNDH	RFLPs from Osborn_lab_RFLPs and Lydiate_lab_RFLPs sets, plus some 'BBSRC' SSRs	pN, pO, pR, pW, Na, Ni, Ol, Ra	Lowe *et al.* 2004	BnaSNDH_02_2004a	35
		RFLPs from Osborn_lab_RFLPs and Lydiate_lab_RFLPs sets	pN, pO, pR, pW, CA, es, IA, IB, IC, ID, IE, IF, IG, IH, mi, T, F	Parkin *et al.* 2005	BnaSNDH_03_2005a	
		'AAFC' SSRs, 'Celera' SSRs, in addition to RFLPs from Osborn_lab_RFLPs	pN, pO, pR, pW, sN, sNR, sOR, sR, sS	Lydiate *et al.* unpublished	Not applicable	
SG	BnaSGDH	RFLPs from Osborn_lab_RFLPs and Lydiate_lab_RFLPs sets, in addition to 'Celera' SSRs, 'AAFC' SSRs and 'BBSRC' SSRs	pN, pO, pR, pW, sN, sNR, sOR, sR, sS, BRAS, CB, MD, MR, Na, Ni, Ol, Ra	Parkin *et al.* unpublished	Not applicable	65
Darmor × Yudal	BnaDYDH	a mixed sets of isozyme, RAPD, RFLP and AFLP markers	Described in Chèvre et al. (1995) and Foisset et al. (1996), E*M*	Lombard and Delourme 2001	BnaDYDH_01_2001a	152
		markers in common with BnaDYDH_01_2001a, plus 'AAFC', 'BBSRC' and 'Celera' SSRs, and some SNP/InDel markers	Same as above, At, pN, pW, sN, sNR, sOR, sR, sS, BRAS, CB, Na, Ol, Ra, IGF	Delourme *et al.* 2008	BnaDYDH_03_2008a	

* Population registry names available online (http://www.brassica.info/resource/plants/mapping-populations.php)

$ Sequence tagged genetic markers are described online from http://www.brassica.info/resource/markers.php.

** Map registry names available online (http://www.brassica.info/resource/maps/published-data.php)
