# Supplementary material for: Integration of linkage maps for the Amphidiploid Brassica napus and comparative mapping with Arabidopsis and Brassica rapa
Source: BMC Genomics. 2011 Feb 9;12:101. doi: 10.1186/1471-2164-12-101 (PMC3042011; doi:10.1186/1471-2164-12-101)

Additional File 9. Diagram of database interaction facilitating the map integration process and establishing links between genetic maps to DNA sequence information (e.g., TAIR9 genome or *B. rapa* BACs) via sequence-tagged marker sequences. CropStoreDB is used to manage data relating to *Brassica* genetics, including populations, genetic maps, genetic markers and their positions. SeqStoreDB is used to manage all publicly available *Brassica* sequences together with sequence data from private sources. AlignStoreDB is used to manage all the homology alignments between query *Brassica* sequences and target genomic or BAC sequences.

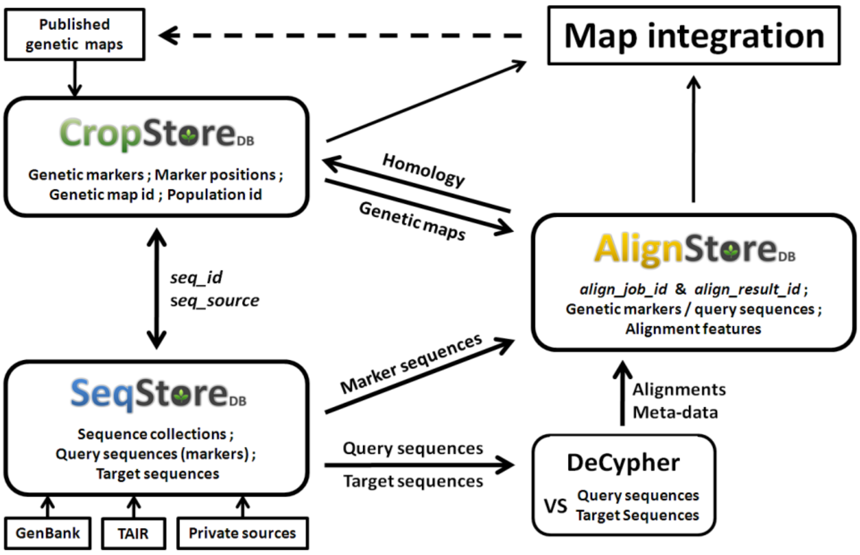

Supplement: Additional file 9 — Diagram of database interaction facilitating the map integration process and establishing links between genetic maps to DNA sequence information (e.g., TAIR9 genome or B. rapa BACs) via sequence-tagged marker sequences. CropStoreDB is used to manage data relating to Brassica genetics, including populations, genetic maps, genetic markers and their positions. SeqStoreDB is used to manage all publicly available Brassica sequences together with sequence data from private sources. AlignStoreDB is used to manage all the homology alignments between query Brassica sequences and target genomic or BAC sequences. [file 1471-2164-12-101-S9.PDF]
